# Pathogenicity and impact of HLA class I alleles in aplastic anemia patients of different ethnicities

**DOI:** 10.1172/jci.insight.163040

**Published:** 2022-11-22

**Authors:** Timothy S. Olson, Benjamin F. Frost, Jamie L. Duke, Marian Dribus, Hongbo M. Xie, Zachary D. Prudowsky, Elissa Furutani, Jonas Gudera, Yash B. Shah, Deborah Ferriola, Amalia Dinou, Ioanna Pagkrati, Soyoung Kim, Yixi Xu, Meilun He, Shannon Zheng, Sally Nijim, Ping Lin, Chong Xu, Taizo A. Nakano, Joseph H. Oved, Beatriz M. Carreno, Yung-Tsi Bolon, Shahinaz M. Gadalla, Steven G.E. Marsh, Sophie Paczesny, Stephanie J. Lee, Dimitrios S. Monos, Akiko Shimamura, Alison A. Bertuch, Loren Gragert, Stephen R. Spellman, Daria V. Babushok

**Affiliations:** 1 Comprehensive Bone Marrow Failure Center and; 2 Division of Oncology, Department of Pediatrics, Children’s Hospital of Philadelphia, Philadelphia, Pennsylvania, USA.; 3 Division of Hematology/Oncology, Department of Medicine, University of Pennsylvania, Philadelphia, Pennsylvania, USA.; 4 Department of Pathology and Laboratory Medicine, Children’s Hospital of Philadelphia, Philadelphia, Pennsylvania, USA.; 5 Department of Pathology and Laboratory Medicine, Tulane University School of Medicine, New Orleans, Louisiana, USA.; 6 Department of Biomedical and Health Informatics, Children’s Hospital of Philadelphia, Philadelphia, Pennsylvania, USA.; 7 Department of Pediatrics, Division of Hematology/Oncology, Baylor College of Medicine, Houston, Texas, USA.; 8 Texas Children’s Cancer and Hematology Center, Houston, Texas, USA.; 9 Dana Farber/Boston Children’s Cancer and Blood Disorders Center, Harvard Medical School, Boston, Massachusetts, USA.; 10 Department of Pediatrics, Dr. von Hauner Children’s Hospital, LMU Klinikum Munich, Munich, Germany.; 11 Sidney Kimmel Medical College, Thomas Jefferson University, Philadelphia, Pennsylvania, USA.; 12 Center for International Blood and Marrow Transplant Research and; 13 Division of Biostatistics, Medical College of Wisconsin, Milwaukee, Wisconsin, USA.; 14 Center for International Blood and Marrow Transplant Research, National Marrow Donor Program/Be The Match, Minneapolis, Minneapolis, USA.; 15 Department of Pathology and Laboratory Medicine, Hospital of the University of Pennsylvania, Philadelphia, Pennsylvania, USA.; 16 Parker Institute for Cancer Immunotherapy and Center for Cellular Immunotherapies, University of Pennsylvania, Philadelphia, Pennsylvania, USA.; 17 Center for Cancer and Blood Disorders, Children’s Hospital Colorado, Aurora, Colorado, USA.; 18 Department of Pediatric Transplant and Cell Therapy, Memorial Sloan Kettering Cancer Center, New York, New York, USA.; 19 Clinical Genetics Branch, Division of Cancer Epidemiology and Genetics, National Cancer Institute, NIH, Rockville, Maryland, USA.; 20 Anthony Nolan Research Institute and University College London Cancer Institute, Royal Free Campus, London, United Kingdom.; 21 Department of Microbiology and Immunology, Medical University of South Carolina, Charleston, South Carolina, USA.; 22 Clinical Research Division, Fred Hutchinson Cancer Research Center, Seattle, Washington, USA.; 23 Department of Pathology and Laboratory Medicine, Perelman School of Medicine, University of Pennsylvania, Philadelphia, Pennsylvania, USA.

**Keywords:** Autoimmunity, Hematology, Autoimmune diseases, Clonal selection, Hematopoietic stem cells

## Abstract

Acquired aplastic anemia (AA) is caused by autoreactive T cell–mediated destruction of early hematopoietic cells. Somatic loss of human leukocyte antigen (HLA) class I alleles was identified as a mechanism of immune escape in surviving hematopoietic cells of some patients with AA. However, pathogenicity, structural characteristics, and clinical impact of specific HLA alleles in AA remain poorly understood. Here, we evaluated somatic HLA loss in 505 patients with AA from 2 multi-institutional cohorts. Using a combination of HLA mutation frequencies, peptide-binding structures, and association with AA in an independent cohort of 6,323 patients from the National Marrow Donor Program, we identified 19 AA risk alleles and 12 non-risk alleles and established a potentially novel AA HLA pathogenicity stratification. Our results define pathogenicity for the majority of common *HLA-A/B* alleles across diverse populations. Our study demonstrates that HLA alleles confer different risks of developing AA, but once AA develops, specific alleles are not associated with response to immunosuppression or transplant outcomes. However, higher pathogenicity alleles, particularly HLA-B*14:02, are associated with higher rates of clonal evolution in adult patients with AA. Our study provides insights into the immune pathogenesis of AA, opening the door to future autoantigen identification and improved understanding of clonal evolution in AA.

## Graphical abstract

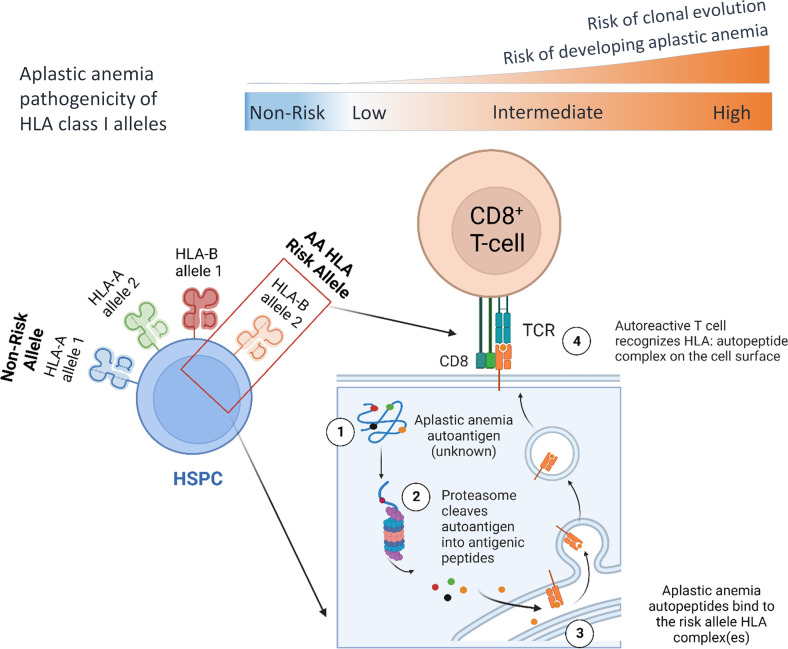

## Introduction

Acquired aplastic anemia (AA) is an autoimmune bone marrow failure disorder caused by T lymphocyte–mediated attack on hematopoietic stem and progenitor cells (HSPCs) (1). Antigenic target(s) of the autoimmune attack remain unknown, and triggers and specific mechanisms of autoimmunity in AA remain poorly understood. Most patients with AA treated with immunosuppressive therapy (IST) develop clonal hematopoiesis, with somatic mutations in surviving hematopoietic cells (2–6). Among the most commonly mutated genes in patients with AA are the human leukocyte antigen (*HLA*) class I genes.

In 2011, Katagiri et al. reported acquired copy number–neutral loss of heterozygosity of chromosome arm 6p (6pCN-LOH) involving the major histocompatibility complex region as a recurrent abnormality in AA and were the first to propose that 6pCN-LOH clones in AA arise due to hematopoietic cells escaping autoimmune attack through HLA class I allele loss (7). The subsequent discovery of inactivating somatic mutations in specific *HLA* class I alleles unambiguously identified *HLA* class I gene inactivation as a key mechanism of clonal evolution in AA that is distinct and noncooperative with genetic mutations and cytogenetic abnormalities that drive transformation to myelodysplastic syndrome (MDS) (2, 4, 8–11). Somatic inactivation of HLA alleles without any other mutations was sufficient for clonal expansion in AA, indicating that it was the loss of targeted alleles that created the survival advantage of *HLA* allele–lacking hematopoietic cells (10, 12–14). The targeted alleles have been presumed to be responsible for AA autoantigen presentation in the affected patients; henceforth these will be referred to as “risk alleles.” Next-generation sequencing (NGS) studies of somatic HLA mutations in AA identified several such *HLA* class I risk alleles, most notably HLA-B*14:02 and HLA-B*40:02, which were also significantly enriched in patients with AA (7, 9–11, 15, 16).

However, despite progress, many questions fundamental for our understanding of the role of HLA class I antigen presentation in AA remain. What determines whether a given HLA allele is able to mediate autoantigen presentation in AA, and are there alleles that cannot? Do AA risk alleles share peptide-binding motifs, and could these be used to infer the properties of AA autoantigen(s)? Given the tremendous diversity of HLA alleles across racial and ethnic groups, is AA in patients of different ethnicities mediated through the same alleles and autoantigen(s)? Finally, while there has been a growing body of literature on the clinical predictive value of having somatic clones with HLA allele loss (7, 11, 15–19), the significance of the patients’ HLA class I repertoire for development of AA and the impact that specific risk alleles have on the patients’ clinical course still remain poorly understood.

In our previous bi-institutional study, we found that the presence of 4 AA HLA risk alleles, particularly HLA-B*14:02, was associated with a higher risk of clonal evolution (10), a finding subsequently confirmed in a larger NIH cohort (15). Our previous study had insufficient patient numbers to systematically evaluate the structural characteristics and clinical significance of HLA alleles in AA. We now present a comprehensive analysis of the significance of HLA class I alleles in AA pathogenesis. Here, we analyzed 505 patients with AA from 2 diverse multi-institutional cohorts (North American Pediatric Aplastic Anemia Consortium, NAPAAC; and Center for International Blood and Marrow Transplant Research, CIBMTR) for HLA loss, identifying 19 distinct AA HLA risk alleles and 12 non-risk (protective) alleles. Comparison of HLA allele mutation frequencies, protein structures, and association with AA in an independent cohort of 6,323 patients from the National Marrow Donor Program (NMDP) allowed us to stratify the full spectrum of pathogenicity of HLA alleles. We then defined the impact of higher pathogenicity alleles (HPA) on outcomes of patients treated with IST and on immunologic complications and outcomes of patients receiving allogeneic hematopoietic stem cell transplant (alloHSCT).

## Results

### Somatic HLA loss in patients with AA.

**Figure 1:**
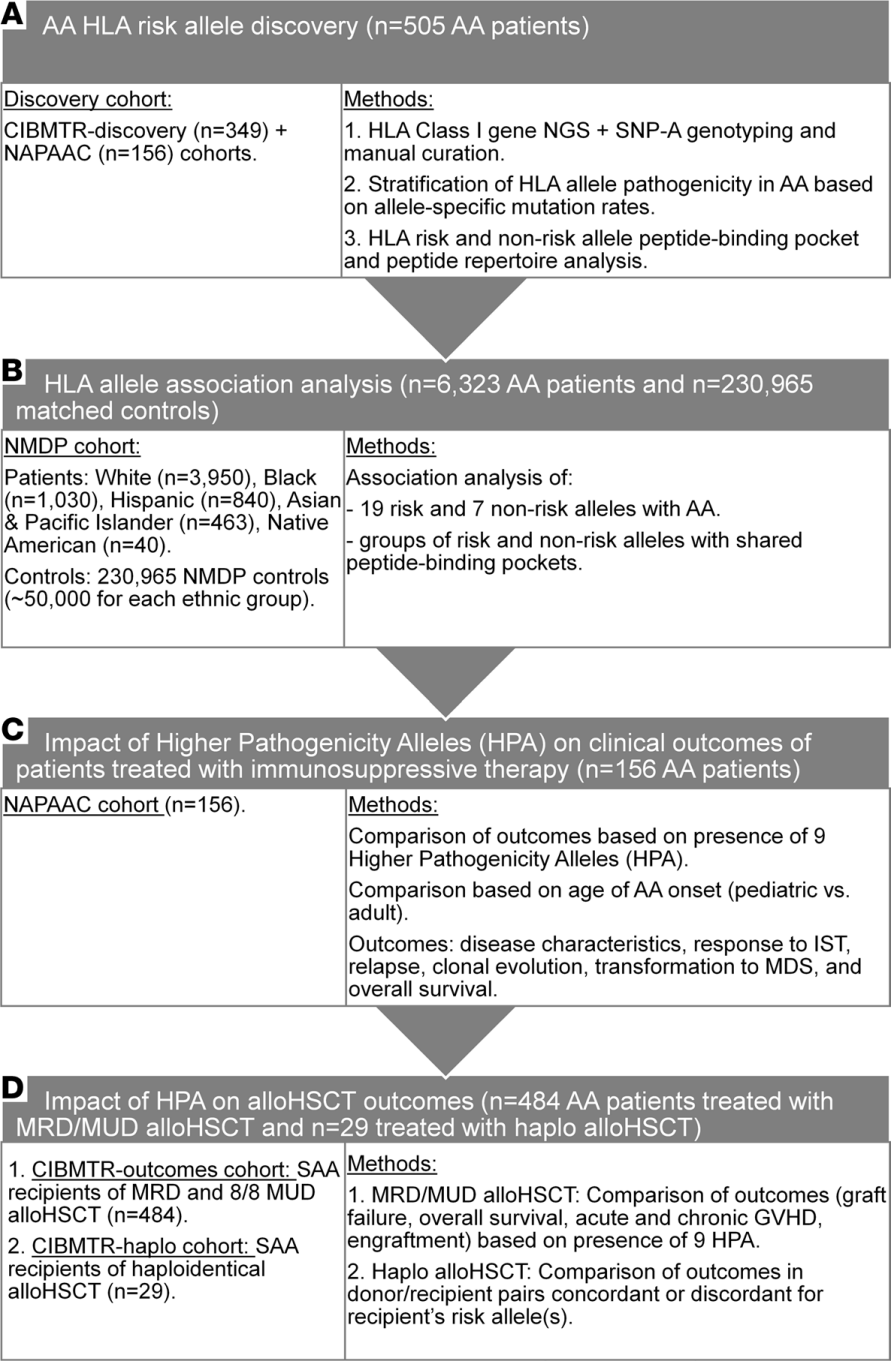
Flowchart summarizing patient cohorts and methods used in this study. The flowchart summarizes the study progression and lists the patient cohorts that were used for each analysis. (**A**) The analysis of somatic HLA loss and discovery of HLA risk alleles were performed in the 505-patient discovery cohort, which was composed of 156 patients from North American Pediatric Aplastic Anemia Consortium (NAPAAC cohort) and 349 patients enrolled in the Center of International Blood and Marrow Transplant Research (CIBMTR-discovery cohort). HLA mutation data generated from the discovery cohort were used for HLA pathogenicity stratification, which led to the identification of HLA risk and non-risk alleles. (**B**) HLA risk and non-risk alleles identified by studies in **A** were then evaluated for association with AA using an independent cohort of 6,323 patients with AA and 230,965 matched controls enrolled in the National Marrow Donor Program (NMDP). (**C**) Analysis of clinical outcomes following immunosuppressive therapy (IST) was performed in the 156-patient NAPAAC cohort, which included both pediatric and adult patients. (**D**) Analysis of clinical outcomes following allogeneic hematopoietic stem cell transplant (alloHSCT) was performed in an independent cohort (CIBMTR-outcomes cohort) of 484 AA patients who received matched related or unrelated donor (MRD/MUD) alloHSCT and were enrolled in the CIBMTR. Outcomes of haploidentical transplant were evaluated in a separate cohort of 29 patients with AA who underwent haploidentical transplant (CIBMTR-haplo). GVHD, graft-versus-host disease; SAA, severe aplastic anemia.

We used a combination of targeted NGS of *HLA* class I genes and single nucleotide polymorphism array (SNP-A) genotyping in 505 AA patients from 2 multi-institutional consortia, NAPAAC (*n* = 156 patients) and CIBMTR-discovery (*n* = 349 patients) (Figure 1 and Supplemental Table 1; supplemental material available online with this article; https://doi.org/10.1172/jci.insight.163040DS1).

**Figure 2:**
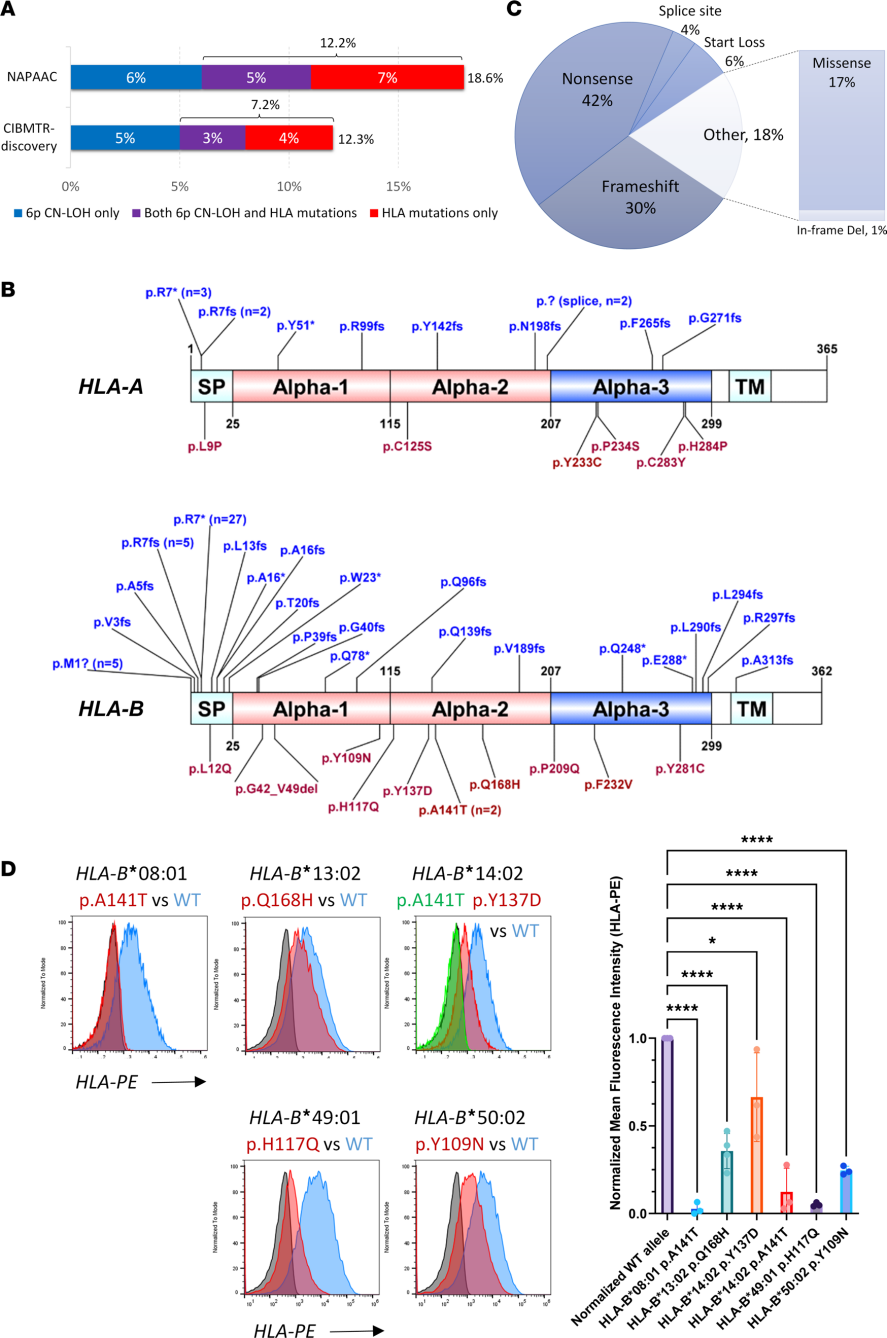
Somatic HLA loss in patients with AA. (**A**) Bar chart showing the frequency of somatic HLA loss in patients with AA in the discovery cohort (CIBMTR-discovery and NAPAAC). Somatic HLA loss was detected as somatic mutations (red and purple bars) and/or acquired 6pCN-LOH (purple and blue bars) in 18.6% of NAPAAC cohort and 12.3% of CIBMTR-discovery cohort. (**B**) A schematic showing 86 somatic HLA mutations identified in the discovery cohort. The domains of HLA molecules include signal peptide (SP), the alpha-1 and alpha-2 domains forming the HLA peptide-binding pocket, and alpha-3 and transmembrane (TM) domains. Loss-of-function mutations due to loss of start (p.M1?), frameshift (fs), splice site (p.? splice), and nonsense variants (*) are shown in blue above the respective genes. Missense and in-frame deletion variants are shown in red below. The numbers refer to the amino acids in the full HLA protein. (**C**) A pie chart showing the breakdown of identified somatic HLA mutations by type. (**D**) Representative flow histograms showing surface HLA expression of mutant HLA alleles. Mutant (red and green) and wild-type (WT, blue) alleles were transfected into cell lines lacking endogenous HLA expression (untransfected, shown in dark gray). *n* = 3–4 replicates per allele, as shown. On the right is the summary analysis showing mean ± standard deviation for percentage of surface HLA expression normalized to the corresponding WT alleles. *P < 0.05, ****P < 0.0001, using ordinary 1-way ANOVA (nonparametric or mixed) in GraphPad Prism.

Somatic HLA loss was present in 18.6% (29 of 156) of NAPAAC patients, including 11.5% (18 of 156) with 6pCN-LOH and 12.2% (19 of 156) with HLA mutations, of whom 5.1% (8 of 156) of NAPAAC patients carried both alterations (Figure 2A). Because the CIBMTR-discovery cohort excluded patients with the 2 most commonly mutated HLA risk alleles, HLA-B*14:02 and HLA-B*40:02, to increase the likelihood of identifying less common risk alleles present in underrepresented racial and ethnic groups, CIBMTR-discovery patients had a lower rate of somatic HLA loss of 12.3% (43 of 349). A total of 30 of 349 CIBMTR-discovery patients (8.6%) had 6pCN-LOH, and 24 of 335 evaluable patients (7.2%) had HLA mutations, of whom 3.3% (11 of 335 evaluable patients) carried both alterations. Patients with somatic alterations had a median of 1 HLA-mutant clone each (range 1–7, with one to four 6pCN-LOH clones and 1 to 5 HLA mutations) (Supplemental Table 2) and did not differ in age (median 15.7 vs. 17.6 years) or prevalence of paroxysmal nocturnal hemoglobinuria (PNH) clones (both 25.1%) from patients without HLA loss.

We identified 86 somatic mutations targeting *HLA-A* and *HLA-B* in 43 patients; there were no mutations in HLA-C (Supplemental Table 2). A total of 44 mutations involved hotspot residues (Figure 2B); 37 involved the previously reported mutation hotspot in *HLA-A/B* exon 1 (10, 15, 16) (c.19C>T, p.R7* in 30 patients and c.16delC, p.R7fs in 7 patients); 5 altered the start codon (c.1A>G/T, p.M1?) in alleles lacking an alternative start at position 4 (Supplemental Figure 1); and 2 changed a highly conserved residue (c.421G>A, p.A141T).

Of the mutations, 81% (70 of 86) were predicted to cause loss of HLA expression through a premature stop or frameshift, splice, or start codon mutations. A total of 15 (17%) were missense mutations, and 1 was an in-frame deletion (Figure 2C). Of these, 2 altered conserved residues in signal peptide (Supplemental Figure 1) and were predicted to disrupt HLA trafficking, and 7 altered alpha-3 domain involved in binding CD8 (20). A total of 7 mutations targeted alpha-1 and alpha-2 domains forming the peptide-binding pocket: of these, 1 was an 8–amino acid deletion predicted to severely disrupt HLA structure, 4 (A141T on 2 alleles, Y137D, and H117Q) abrogated surface HLA expression, another (Y109N) significantly reduced HLA expression, and 1 (Q168H) preserved partial HLA expression with the potential to alter peptide binding (Figure 2D).

### A total of 19 AA HLA risk alleles belong to 6 HLA supertypes.

**Figure 3:**
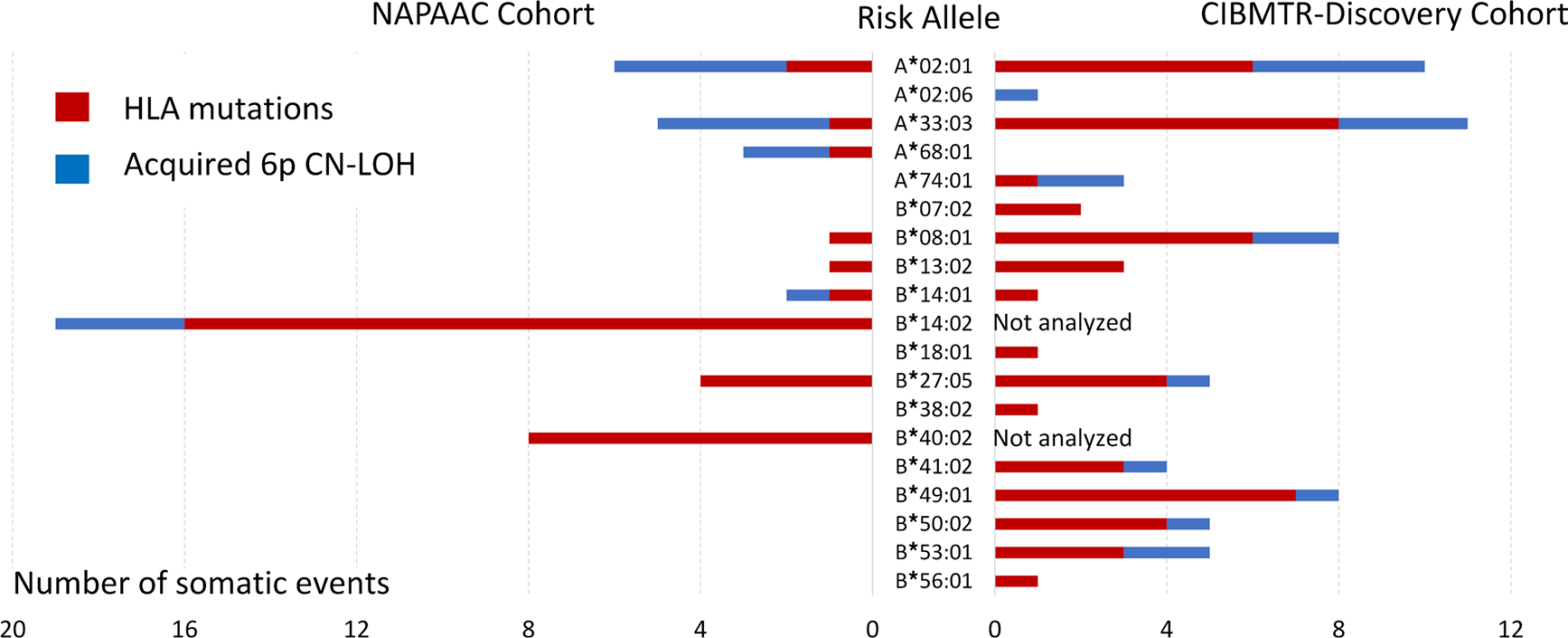
The number of clones with HLA loss identified in the discovery cohort for 19 HLA risk alleles. Risk alleles are listed along the *y* axis. Mutations (red bars) and 6pCN-LOH events (blue bars) for NAPAAC cohort are shown on the left and CIBMTR (discovery) on the right.

Somatic mutations targeted 18 distinct HLA class I risk alleles (Figure 3). In patients who had more than 1 clonally expanded population with somatic HLA loss, all alterations (somatic mutations and 6pCN-LOH) led to the loss of the same *HLA* allele, supporting the hypothesis that in each affected patient, a single targeted risk allele was mediating autoantigen presentation and was responsible for the autoimmune attack. Of 29 patients with 6pCN-LOH but no mutations, we were able to unambiguously determine the deleted HLA haplotype in 23 (Supplemental Table 2). In 16 of these 23 patients, the deleted haplotype contained at least 1 known risk allele. In 1 patient, the involved region included only the *HLA-A* locus, allowing us to identify 1 additional risk allele (HLA-A*02:06), thus bringing the total identified risk alleles to 19. Approximately 90% of the discovery cohort patients carried 1 or more of the 19 identified risk alleles: 34.7% had 1, 41% — 2, 10.7% — 3, and 1.2% — 4 risk alleles.

**Figure 4:**
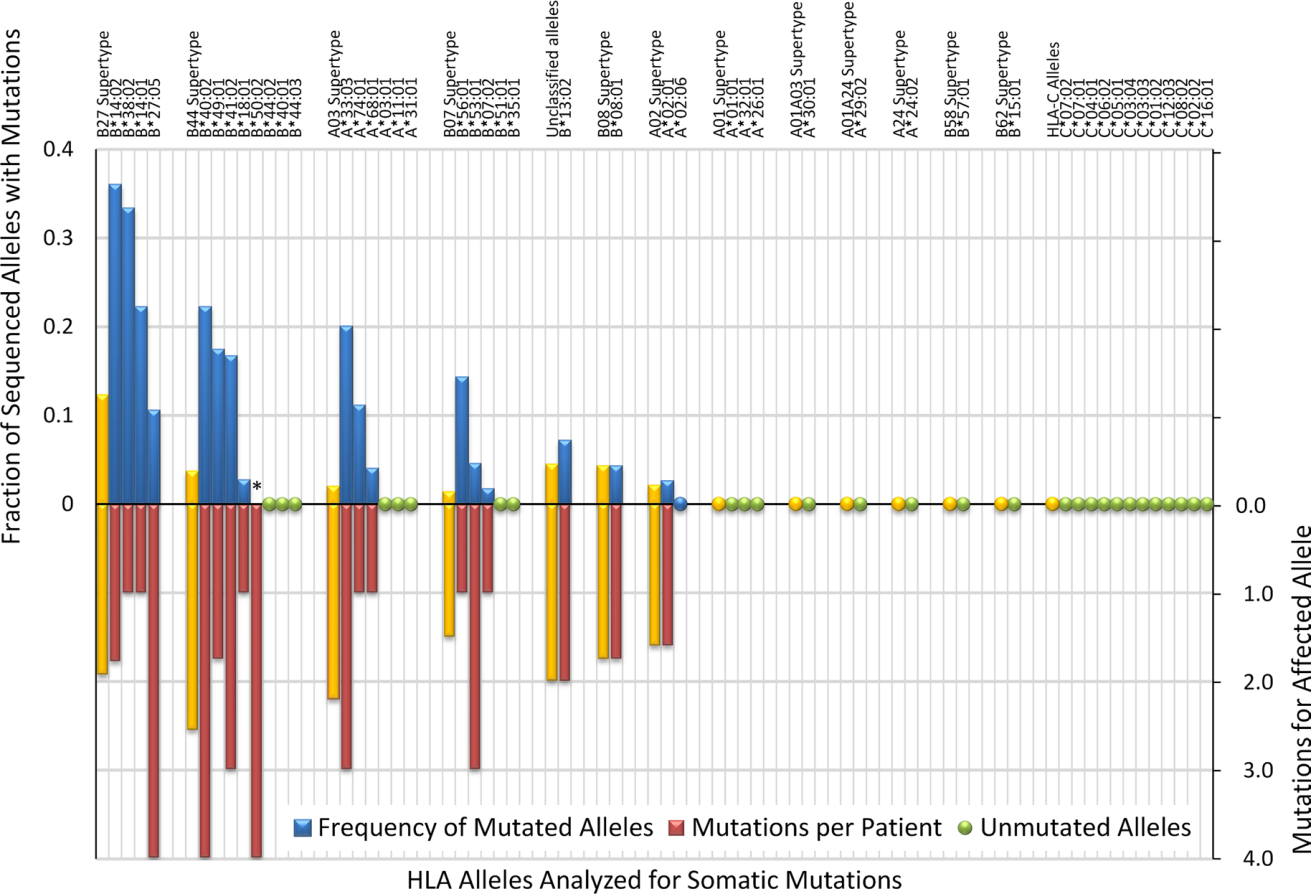
Frequency of mutations per allele. Alleles are grouped by HLA supertype, with combined mutation frequency for each supertype shown in yellow bars. Mutation frequency is shown as blue bars above and mutations per patient as red bars below *x* axis. *HLA-B*50:02 was analyzed in a single patient, and mutation frequency could not be determined. Alleles with at least 20 analyzed patients without mutations are shown by green dots.

Sixteen of the 19 identified AA HLA risk alleles belonged to 6 structurally related groups with shared peptide-binding specificities called HLA supertypes (21): B44 (*n* = 4 alleles), B27 (*n* = 3), A03 (*n* = 3), B07 (*n* = 3), A02 (*n* = 2), and B08 (*n* = 1) (Figure 4). Of 3 risk alleles with no assigned supertype, HLA-B*38:02 had features of B27 and HLA-B*49:01 of B44 supertype (21).

### HLA allele pathogenicity stratification rating.

We reasoned that because the selective pressure on HSPCs to lose HLA alleles may reflect the role these alleles play in mediating autoimmune attack in patients with AA, the prevalence of somatic allele loss may indicate how frequently a given allele is responsible for autoimmune recognition in AA. We thus systematically compared mutation frequencies for 161 distinct HLA class I alleles (45 *HLA-A*, 75 *HLA-B*, and 40 *HLA-C*) in the discovery cohort. Individual alleles were present in a median of 4 patients (range 1 to 193), with 37 alleles (11 *HLA-A*, 14 *HLA-B*, and 12 *HLA-C*) present in at least 20 patients.

**Figure 5:**
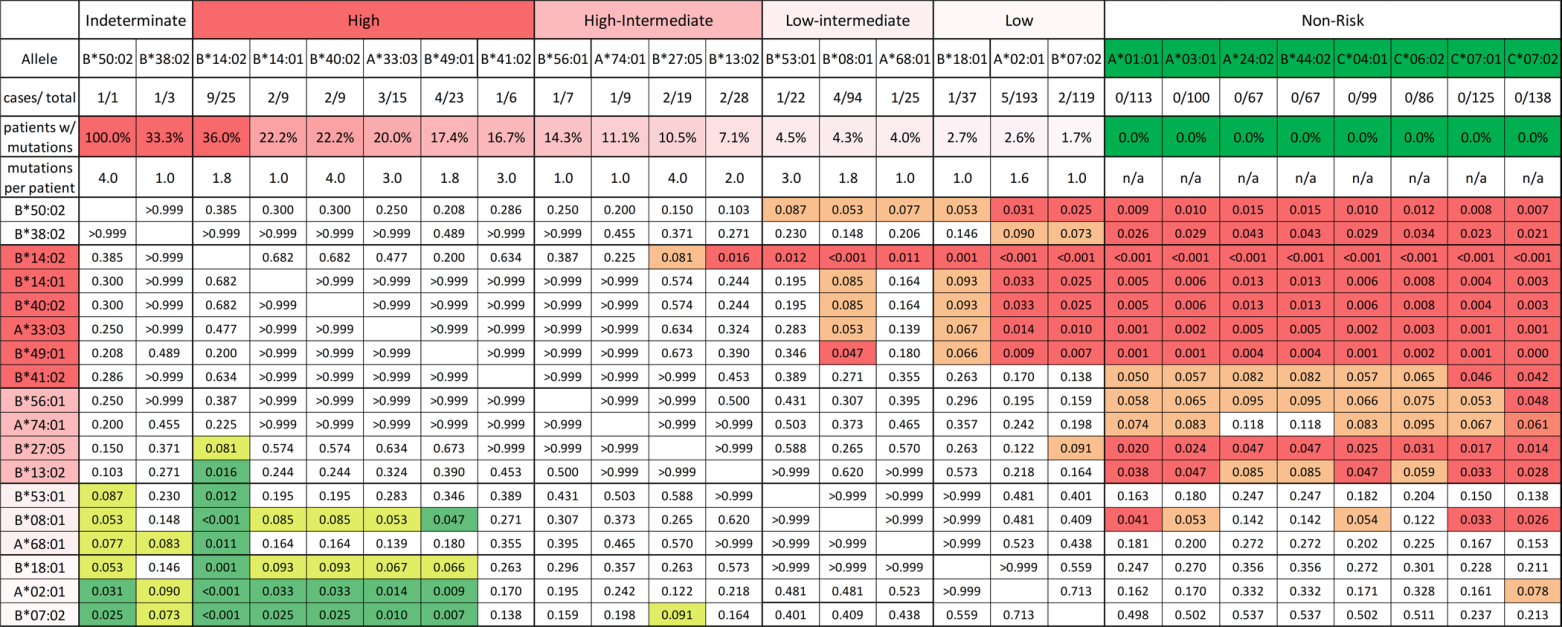
Pathogenicity stratification of 18 HLA class I risk alleles with identified somatic mutations and other alleles analyzed in at least 20 patients. Shown is a pairwise comparison matrix of relative mutation frequencies per individual HLA alleles HLA alleles are listed along the *x* axis, grouped by pathogenicity ranking. High-risk, high-intermediate, low-intermediate, and low-risk alleles are shown in decreasing intensity of red and non-risk alleles in green. B*38:02 and B*50:02 were insufficiently prevalent in the cohort for analysis — these were listed as indeterminate. The number of patient cases with mutations, total patients with listed allele, percentage mutation frequency, and median number of mutations per patient are listed in the header rows below each allele. Pairwise comparison of mutation frequencies is shown as a correlation matrix. Comparisons were performed by Fisher’s exact tests, with *P* value listed for each pair of alleles. *P* values < 0.05 are shaded in dark red for comparison OR > 1 or dark green for OR < 1. Trends with *P* ≥ 0.05 and *P* < 0.1 are shown in light red for OR > 1 and light green for OR < 1.

Prevalence of mutations in patients carrying a given allele ranged from 36% (9 of 25 allele-carrying patients) for HLA-B*14:02 to 1.7% (2 of 119) for HLA-B*07:02 (*P* < 0.001) (Figures 4 and 5). Alleles from B27 supertype were mutated significantly more frequently (12%, 14 of 114 allele-carrying patients) than alleles from other supertypes (B08 supertype — 4.3%, B44 — 3.6%, A02 — 2.1%, A03 — 2.0%, B07 — 1.3%, and 0% for A01, A01A03, A01A24, A24, B58, or B62 supertypes; *P* < 0.05). HLA alleles were targeted by mutations at significantly different frequencies, allowing us to cluster the alleles into 5 different pathogenicity risk groups, defined by differences in mutation frequency: high (>15%), high-intermediate (>5%–15%), low-intermediate (>3%–5%), low (≤3%), and non-risk (0%) (Figure 5).

High-risk alleles (HLA-B*14:02, HLA-B*14:01, HLA-B*40:02, HLA-A*33:03, HLA-B*49:01, and HLA-B*41:02) were mutated in 16.7% to 36% of high-risk allele carriers, with a significantly higher mutation frequency than low-risk alleles (HLA-B*18:01, HLA-A*02:01, and HLA-B*07:02), which were mutated in only 1.7% to 2.7% of allele carriers. High-intermediate alleles (HLA-B*56:01, HLA-A*74:01, HLA-B*27:05, and HLA-B*13:02) were mutated in 7.1% to 14.3% of carriers, while low-intermediate alleles (HLA-B*53:01, HLA-B*08:01, and HLA-A*68:01) were mutated in 4.0% to 4.5% of allele carriers. A total of 8 very common alleles (HLA-A*01:01, HLA-A*03:01, HLA-A*24:02, HLA-B*44:02, and *HLA-*C alleles HLA-C*04:01, HLA-C*06:02, HLA-C*07:01, HLA-C*07:02), none of which were mutated in our cohort, were statistically significantly less likely to be mutated than high and high-intermediate risk alleles; these were classified as non-risk (Figure 5). Twenty additional alleles without mutations were analyzed in 20 or more patients, providing sufficient statistical power to conclude that their pathogenicity is at most low risk; these are listed as low or non-risk in Supplemental Figure 3. High-risk alleles had an average of 2.4 mutations/patient (range 1–4) while low-risk alleles had an average of 1 mutation/patient (range 1–1.6) (Figure 5).

A total of 38 patients in the discovery cohort carried 2 or more risk alleles and had HLA mutations, allowing us to infer the single risk allele mediating autoimmune recognition in each patient. Consistent with their pathogenicity rating, high-risk alleles were inferred to mediate autoimmune recognition in 92.3% of patients (12 of 13), high-intermediate in 83.3% of patients, and low-intermediate in 38.5% of patients (Supplemental Table 3). In contrast, low-risk alleles were nearly always uninvolved and were inferred to mediate autoimmune recognition in only 8.7% (2 of 23) of patients.

### Structural characteristics of AA risk and non-risk HLA alleles.

**Figure 6:**
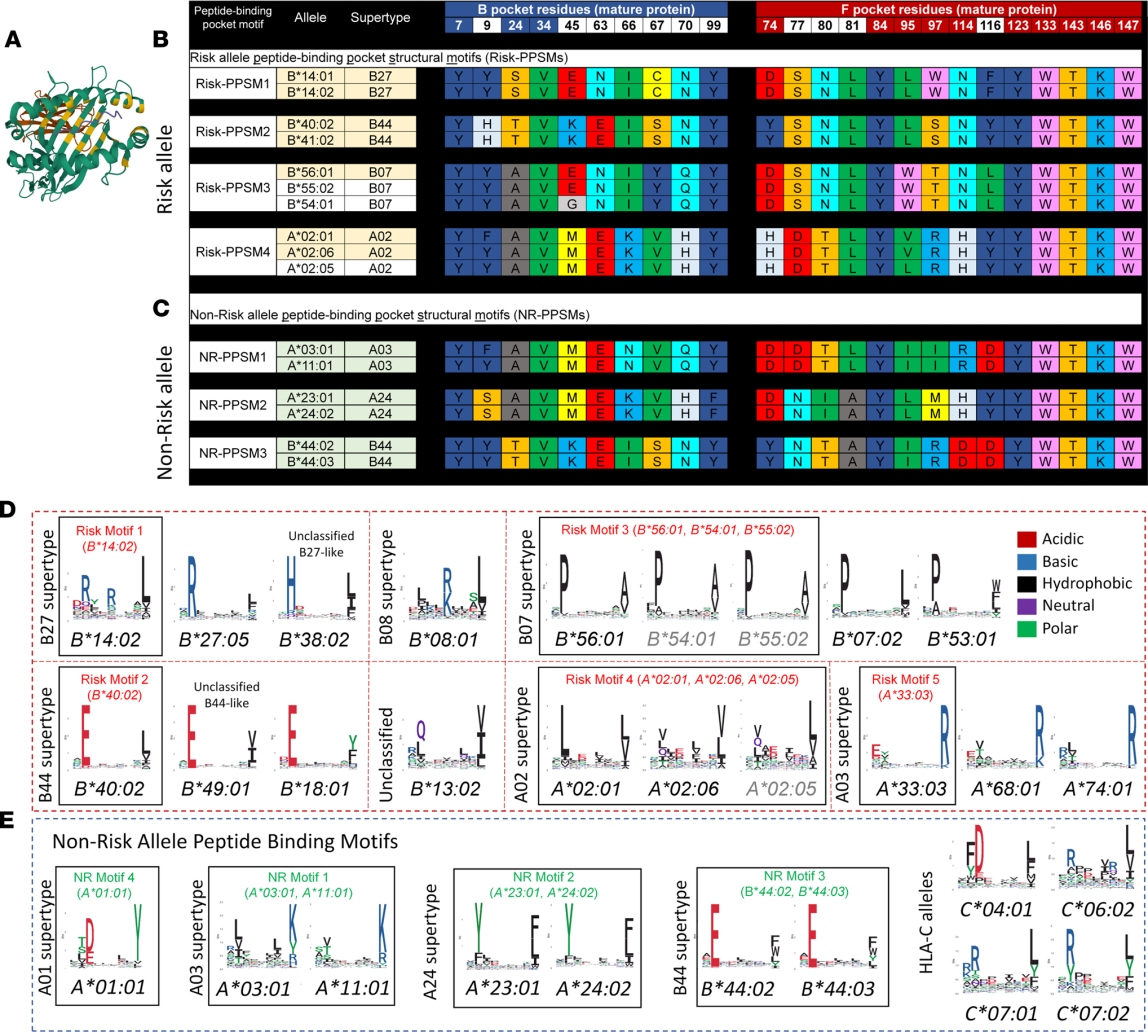
Comparisons of peptide-binding pocket residues and peptide repertoire motifs for AA risk and non-risk HLA alleles. (**A**) A high-resolution crystal structure of HLA-B*14:02 (Protein Data Bank 3BVN; ref. 50), highlighting the location of previously established key residues (shown in yellow, listed in **B**) within the peptide-binding groove, which determines peptide-binding specificity for each allele. (**B** and **C**) Alignment of key residues in the HLA-binding groove showing peptide-binding pocket structure for groups of AA risk (**B**) and non-risk (**C**) alleles. Amino acids in alignment are listed using a single-letter amino acid code and colored using the “Rasmol/shapely” color scheme according to amino acid properties: D, E — bright red; C, M — bright yellow; K, R — medium blue; S, T — orange; F, Y — dark blue; Q, N — cyan, G — light gray; L, V, I — green; A — dark gray; W — pink; H — pale blue. Three risk alleles (HLA-B*55:02, HLA-B*54:01, and HLA-A*02:05), previously reported by other groups (11, 13, 15, 16, that share peptide-binding pocket structures with our identified risk alleles are included in the analysis. (**D** and **E**) Shown are the logo plots of 9–amino acid HLA class I peptides plotted based on experimentally obtained immunopeptidome data from HLA class I monoallelic cell lines (23). Logo plots for identified risk alleles with available immunopeptidome data are shown in **D** and for non-risk alleles in **E**. Alleles are grouped by HLA supertype assignment and peptide-binding pocket identify. Risk and non-risk (NR) supermotifs characterizing groups of alleles based on peptide-binding pocket identity are labeled. Of note, non-risk alleles HLA-B*44:02 and HLA-B*44:03 share the B44 supermotif with HLA-B*40:02 but have distinct requirements for aromatic and polar residues at the C-terminus.

The repertoire of peptides capable of binding to an HLA molecule is determined by critical amino acids that comprise the HLA peptide–binding groove and make contact with the anchor residues of the peptide (22) (Figure 6A). To define structural elements of AA HLA risk alleles, we aligned previously defined critical residues of the HLA-binding pockets (22) in AA risk and non-risk alleles.

Strikingly, several risk alleles had identical or nearly identical peptide-binding pocket structural motifs (PPSMs), indicating that they likely bind the same (or highly similar) AA autoantigen(s) (Figure 6B). We identified 4 distinct risk PPSMs: risk-PPSM1 shared by HLA-B*14:02 and HLA-B*14:01; risk-PPSM2 by HLA-B*40:02 and HLA-B*41:02; risk-PPSM3 by HLA-B*56:01 and 2 previously reported risk alleles, HLA-B*55:02 and HLA-B*54:01 (13, 16); and risk-PPSM4 by HLA-A*02:01, HLA-A*02:06, and a previously reported risk allele, HLA-A*02:05 (15). Alleles with shared PPSMs had similar AA pathogenicity (e.g., risk-PPSM1 and risk-PPSM2 allele pairs are high-risk, Figure 5).

Non-risk alleles also formed groups with distinct pocket residues, suggesting that certain peptide-binding structures are less suitable to binding AA autoantigens. Four non-risk (NR) PPSMs were identified: NR-PPSM1 shared by HLA-A*03:01 and HLA-A*11:01; NR-PPSM2 by HLA-A*24:02 and HLA-A*23:01 (a less common allele in discovery cohort with no mutations in 11 analyzed patients), and NR-PPSM3 by HLA-B*44:02 and HLA-B*44:03 (Figure 6C), bringing the number of non-risk alleles to 7.

The identified peptide-binding pocket structures corresponded to distinct peptide repertoires, which can be represented by peptide motifs (peptide anchor residues necessary for binding a given allele) (Figure 6, D and E). Using experimentally determined peptidome data available for 16 risk alleles (23), we identified 5 distinct peptide motifs, likely corresponding to distinct AA autopeptides originating from a larger shared protein (or similar proteins) (Figure 6D). As expected, peptide repertoires of risk alleles differed from those of non-risk alleles (Figure 6E).

### HLA risk alleles predispose to AA while non-risk alleles do not mediate AA.

**Figure 7:**
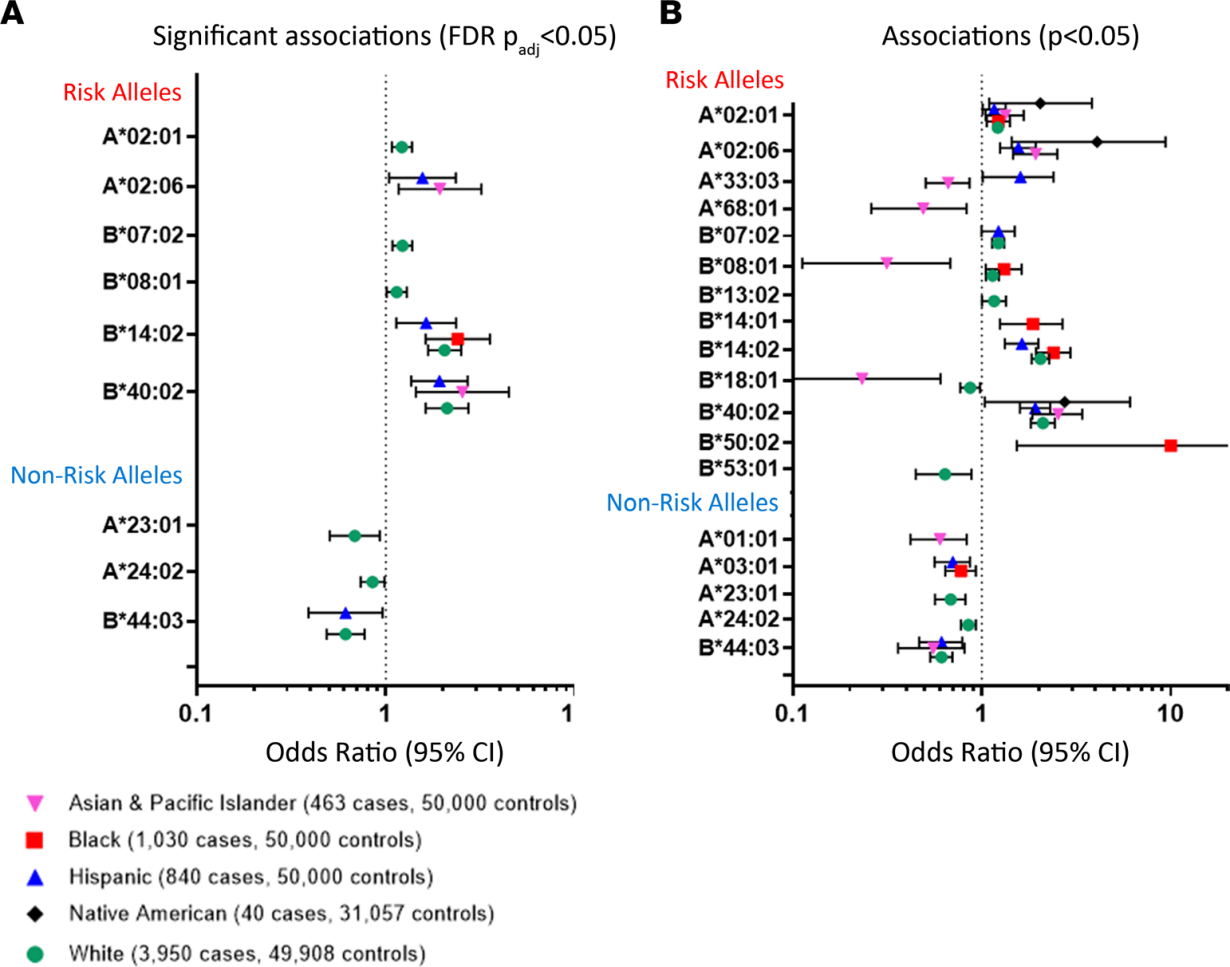
Association analysis of risk and non-risk HLA alleles with AA in the NMDP cohort. Shown are statistically significant associations of between risk and non-risk HLA-A and -B alleles in different racial and ethnic groups, using the higher stringency multiple-testing adjustment FDR *P*_adj_ < 0.05 (**A**) and unadjusted *P* < 0.05 (**B**). ORs with 95% CI are shown for individual alleles, color-coded for different ethnicities (Asian & Pacific Islander — pink downward triangles; Black — red squares; Hispanic — blue upward triangles; Native American — black diamonds; White — green circles). All details for association study analysis for the risk and non-risk HLA alleles for each of the ethnic groups, including the numbers of patients with AA and controls with each allele; the frequencies of alleles in patients and controls; the ORs; the 95% CIs; and the *P* values, including *P* values adjusted for multiple testing, are included in Supplemental Table 5.

To test the hypothesis that HLA risk alleles may predispose to AA at higher rates than non-risk alleles, we performed an association analysis in 6,323 patients with AA (3,979 White, 1,030 Black, 841 Hispanic, 463 Asian and Pacific Islander, and 40 Native American) and 230,965 NMDP healthy donor controls, matched for racial and ethnic group (31,057 to 50,000 per ethnic group, based on availability, detailed in Figure 7 and Supplemental Table 4).

After adjusting for the FDR due to multiple allele testing (24), 6 risk alleles (HLA-A*02:01, HLA-A*02:06, HLA-B*07:02, HLA-B*08:01, HLA-B*14:02, and HLA-B*40:02) were significantly overrepresented in patients with AA compared with controls in at least 1 racial or ethnic group (*P*_adjusted_ _FDR_ < 0.05) (Figure 7A and Supplemental Table 5, A–E). To explore potential associations with rare HLA alleles and to evaluate for HLA associations in less well represented racial and ethnic groups, we also reviewed the raw analysis without FDR adjustment, as has been done in all previous AA HLA association studies (7, 10, 15, 25–28). Using unadjusted analysis, 10 of 19 identified AA HLA risk alleles were significantly enriched in AA (Figure 7B). The association with AA was strongest for high-risk alleles, with ORs for significant associations ranging in different ethnic groups from 1.35 to 2.15 for HLA-B*14:01, 1.83 to 2.17 for HLA-B*14:02, and 1.77 to 2.70 for HLA-B*40:02, while low-risk alleles had OR 1.25 for HLA-A*02:01 and 1.19 to 1.29 for HLA-B*07:02 (Figure 7A and Supplemental Table 5, A–E, for ORs with 95% CIs for each allele in different ethnicities).

In contrast to risk alleles, 5 of 7 non-risk alleles (HLA-A*01:01, HLA-A*03:01, HLA-A*23:01, HLA-A*24:02, HLA-B*44:03) were significantly underrepresented in AA in at least 1 ethnic group, consistent with their protective role in AA (Figure 7 and Supplemental Table 5). Of these, HLA-A*03:01 and HLA-B*44:03 were protective in multiple ethnicities with approximately 20% to 30% (OR range 0.69–0.79) and approximately 45% to 55% (OR range 0.45–0.63) AA risk reduction, respectively.

Association analysis further clarified the pathogenicity of 10 *HLA-A/B* alleles, which we estimated as being either low-risk or non-risk based on lack of HLA mutations in the discovery cohort (Supplemental Figure 3, and Supplemental Table 6, A–E). Five alleles (HLA-A*26:01, *HLA-A* *29:02, HLA-B*15:01, HLA-B*25:01, HLA-B*57:01) were negatively associated with AA, of which HLA-A*26:01, HLA-A*29:02, and HLA-B*57:01 were 20% to 30% protective in several ethnicities — these 5 alleles were reclassified as non-risk. In contrast, HLA-A*32:01 was enriched in White (OR 1.15), and HLA-B*40:01 in Asian (OR 1.36), patients. Rare mutations in HLA-B*40:01 were previously reported, consistent with it being a low-risk allele (15, 16).

### Population-based drivers of AA.

We determined AA pathogenicity for the common *HLA-A* and *HLA-B* alleles accounting for approximately 85% of White, 75% Native American, 71% Hispanic, 62% Asian, and 60% Black NMDP populations. The remaining *HLA-A* and *HLA-B* alleles (~15% alleles in White to 38%–40% in Asian and Black NMDP populations) were rare and insufficiently represented in the discovery cohort to assess pathogenicity. When analyzed as a group, the remaining HLA-A alleles were negatively associated with AA in Native American and White people, and the remaining HLA-B alleles were negatively associated with AA in Black, Hispanic, and White populations, indicating that the majority of AA risk carried by HLA class I alleles in Black, Hispanic, Native American, and White populations was captured by analysis of our diverse discovery cohort (Supplemental Table 7).

**Figure 8:**
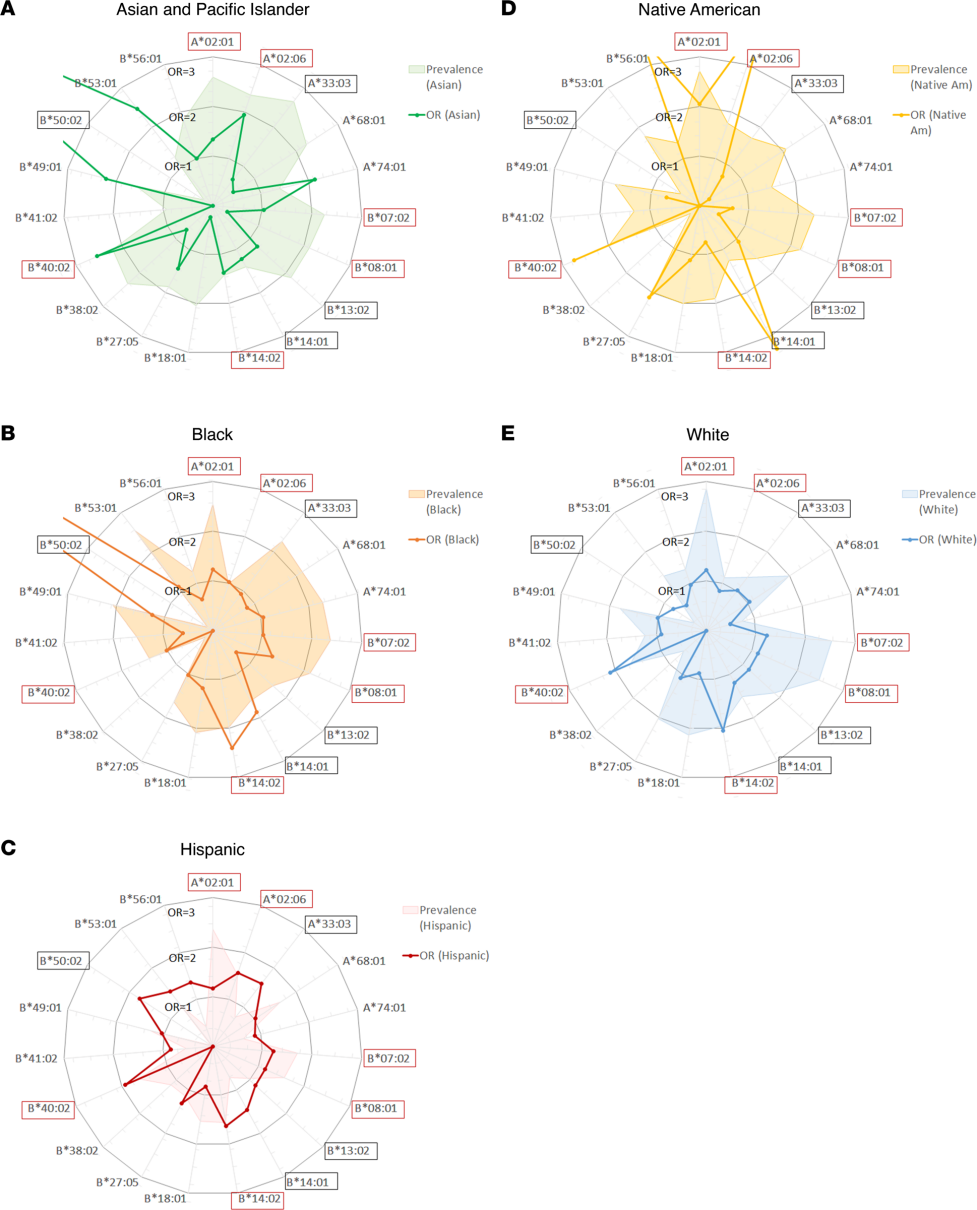
Risk allele contributions to AA and their relative prevalence across populations. Radar plots showing the OR for association with AA (line plot) and population prevalence (shaded area plot) for 19 HLA class I risk alleles in Asian and Pacific Islander (**A**), Black (**B**), Hispanic (**C**), Native American (**D**), and White (**E**) populations. Alleles are listed around the perimeter of the circle in alphanumeric order, highlighted by red outline for FDR *P*_adj_ < 0.05 from Figure 7A and black outline for *P* < 0.05 in Figure 7B. The line plots show the relative contributions of individual alleles as reflected by the OR of the association with AA; circular grid lines mark OR intervals of 1 (with the inner circle, where OR = 1, indicates the threshold outside of which there is a positive association with AA). Several rare alleles had OR > 3, which were allowed to be off scale to enable a clear depiction of smaller but significant associations. The relative prevalence of alleles is shown by shaded area plots; these are depicted schematically using a different (logarithmic) scale to facilitate visualization of both very common and very rare alleles.

**Figure 9:**
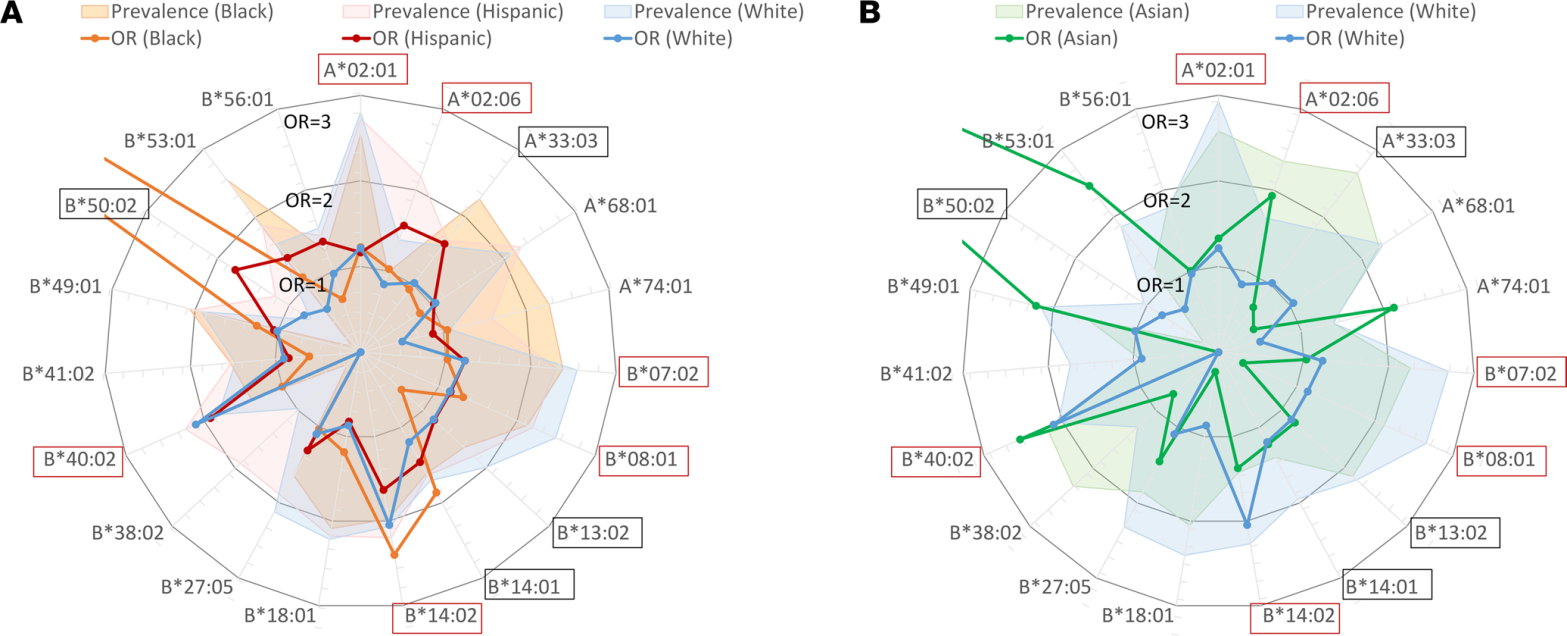
Comparisons of risk allele contributions to AA and their relative prevalence across populations. (**A**) Black, Hispanic, and White. (**B**) Asian and White. Alleles are listed around the perimeter of the circle in alphanumeric order, highlighted by red outline for FDR *P*_adj_ < 0.05 from Figure 7A and black outline for *P* value < 0.05 in Figure 7B. The line plots show the relative contributions of individual alleles as reflected by the OR of the association with AA; circular grid lines mark OR intervals of 1 (with the inner circle, where OR = 1, indicating the threshold outside of which there is a positive association with AA). Several rare alleles had OR > 3, which were allowed to be off scale to enable a clear depiction of smaller but significant associations. The relative prevalence of alleles is shown by shaded area plots; these are depicted schematically using a different (logarithmic) scale to facilitate visualization of both very common and very rare alleles.

A comparison of AA risk allele associations across racial and ethnic groups demonstrated a contribution to AA risk from a common set of alleles (HLA-A*02:01, HLA-B*07:02, HLA-B*08:01, HLA-B*14:01, HLA-B*14:02, and HLA-B*40:02) (Figure 8 and Figure 9) but also identified alleles contributing disproportionately in specific populations. In Asian patients, AA was primarily driven by HLA-B*40:02, with strong contributions from HLA-A*02:06, HLA-A*74:01, and HLA-B*53:01, but virtually no contribution from HLA-B*14:02. Black patients had higher contributions from HLA-B*14:01 and HLA-B*49:01, and HLA-B*50:02, but virtually no contribution from HLA-B*40:02. Contributions to AA were similar in the White and Hispanic AA populations, but HLA-A*02:06, HLA-A*33:03, HLA-B*50:02, and HLA-B*53:01 contributed more in Hispanic patients.

### HPA may predispose to MDS-associated clonal evolution in adult-onset AA.

In our 2017 study of HLA risk alleles in 66 patients with AA, we reported a more complicated disease course with higher rates of MDS (10). We now added 90 new patients to more than double our previous cohort and used this expanded (NAPAAC) cohort to determine the impact of HLA risk alleles on clinical outcomes of patients treated with IST. Of 156 patients, 121 had pediatric-onset AA and 23 adult-onset AA; for another 12 patients, age at AA onset was unknown (Supplemental Tables 8 and 9). Among patients, 85.2% had severe or very severe AA, and 78.5% of the cohort received frontline IST with antithymocyte globulin and cyclosporine. Median duration of follow-up, censored for transplantation, was 5.6 years.

Because all patients with AA patients have HLA class I alleles mediating autoimmune recognition, albeit of varying pathogenicities, we focused on the role of HPA, stringently defined as high- or high-intermediate risk alleles (Figure 5 and Supplemental Methods). Nine alleles (HLA-A*33:03, HLA-B*13:02, HLA-B*14:01, HLA-B*14:02, HLA-B*27:05, HLA-B*40:02, HLA-B*41:02, HLA-B*49:01, HLA-B*56:01) were categorized as HPA. Patients without HPA were analyzed in the group with lower pathogenicity alleles (LPA).

Patients with HPA (*n* = 62) did not differ from patients with LPA (*n* = 94) in age at AA onset, severity, marrow cellularity, responses to IST at 6 months, risk of relapse, second-line therapies, death, or duration of follow-up (Supplemental Tables 8 and 9). However, in agreement with our earlier findings (10), patients with HPA were more likely to develop MDS (10.2% vs. 1.4%, OR 8.07, *P* = 0.039), had more 6pCN-LOH clones (19.4% vs. 6.4%, OR 3.52, *P* = 0.020), and had a non–statistically robust trend toward more cytogenetic abnormalities (15.9% vs. 4.8%, OR 3.67, *P* = 0.089) (Table 1 and Table 2). Of 6 patients who developed post-AA MDS, 5 had HPA.

The effect of HPA on clonal evolution depended on age of AA onset. Pediatric-onset patients with HPA developed 6pCN-LOH clones at higher rates than those with LPA (21.3% vs. 6.8% OR 3.73, *P* = 0.024). In contrast, rates of MDS-associated mutations, cytogenetic abnormalities, or MDS transformation did not significantly differ in pediatric-onset patients by presence or absence of HPA (although the power to detect differences was limited by low rate of MDS-associated clonal evolution in pediatric-onset AA, ref. 29). In contrast, adult-onset patients with HPA were significantly more likely to develop karyotypic abnormalities (50% vs. 0%, OR 19, *P* = 0.033) than adult-onset patients with LPA and had a non–statistically robust trend toward more MDS-associated mutations (70% vs. 27.3%, OR 6.22, *P* = 0.086) and MDS transformation (33.3% vs. 0%, OR 12.18, *P* = 0.093).

Age-related differences were strongly associated with presence of HPA. Adult-onset HPA patients had more MDS-associated mutations (70% vs. 6.3%, OR 35, *P* < 0.001), cytogenetic abnormalities (50% vs. 5.9%, OR 16, *P* = 0.004), and MDS transformation (33.3% vs. 2.7%, OR 18, *P* = 0.010) compared with pediatric-onset HPA patients. However, no significant age-related differences in MDS-associated evolution were seen in patients with LPA.

At an individual allele level, adult-onset patients with HLA-B*14:02 had more MDS transformation (4 of 6, 66.7%) compared with adult-onset patients lacking HLA-B*14:02 (0 of 17, 0%, *P* = 0.002). Pediatric-onset patients with HLA-B*14:02 did not differ in MDS rate from patients lacking HLA-B*14:02 (1 of 10, 10% vs. 1 of 87, 1.1%, *P* = 0.197) (albeit with the limitation of statistical power given the low rate of pediatric MDS). Other alleles in the HPA group were insufficiently represented in the NAPAAC cohort and did not allow for statistical power to analyze contributions of individual alleles other than HLA-B*14:02.

### HLA risk alleles do not affect AA alloHSCT outcomes.

Graft failure is a feared complication of alloHSCT in AA that occurs in up to 21% of AA alloHSCT recipients and in many cases is thought to be mediated by an immunologic rejection of the HLA-matched graft by the patient’s T cells (30). The higher frequency of immunologic graft rejection after alloHSCT in AA versus other diseases is thought to be due to the preexisting T cell clone that targets HSPCs via HLA allele-restricted immunity and that was responsible for driving initiation of AA. We thus sought to determine the effect of HPA on frequency of graft failure and other relevant alloHSCT outcomes in an independent CIBMTR-outcomes cohort of 484 patients with AA who received an HLA-matched bone marrow graft (MRD or 8/8 MUD) (Supplemental Figure 4). Within this cohort, 34.3% (*n* = 166) had HPA, 79% of patients previously had IST, 84% received alloHSCT from MUD, 89% received bone marrow grafts, 98% of patients received nonmyeloablative regimens, and 70% received methotrexate and calcineurin inhibitor–based GVHD prophylaxis. HPA and LPA groups were well balanced, with the exception of more recent transplants in the HPA group (Supplemental Table 10).

In the whole cohort, 1-year incidence of graft failure was 8.6% and 1-year overall survival (OS) was 86.9%. After adjusting for significant covariates, there was no significant association between HPA carrier status and patient outcomes (Table 3 and Supplemental Table 11). HPA patients had similar incidence of graft failure, OS, acute or chronic GVHD, and neutrophil and platelet engraftment. Consistent with prior studies, OS was worse in older patients, those with poor performance status, and patients receiving unrelated donor grafts (31–33).

To assess whether presence of shared risk alleles between haploidentical donors and patients adversely affects outcomes of haploidentical alloHSCT — e.g., by allowing putative AA autoantigen(s) in the haploidentical graft to be presented by a shared risk allele and potentiating graft rejection — we compared outcomes of 29 patients who received haploidentical grafts from donors matched (HPA-concordant, *n* = 15) or mismatched (HPA-discordant, *n* = 14) for HPA of the recipient (CIBMTR-haplo cohort). There were no significant differences in 1- and 2-year graft failure rates or platelet and neutrophil engraftment based on donor risk allele status (Supplemental Tables 12 and 13). Similarly, no differences in outcomes were seen when all 19 risk alleles were considered (Supplemental Tables 14 and 15).

## Discussion

Our results, based on several large independent cohorts of AA patients, provide a comprehensive analysis of the spectrum of HLA class I contributions to AA pathogenesis. Using a combination of frequency of HLA mutations, peptide-binding specificities, and association with AA, we identified 19 AA risk alleles. Additionally, we identified 12 non-risk (protective) alleles and established a pathogenicity stratification for HLA class I alleles in AA. Our results define AA pathogenicity for *HLA-A* and *HLA-B* alleles for >60%–85% of individuals in diverse populations and demonstrate HLA drivers of AA in different races and ethnicities. Our study demonstrates that while individual *HLA-A* and *HLA-B* alleles convey distinct risks of developing AA, once AA occurs, allele-specific patterns of autoimmune recognition do not appear to significantly alter immunologic outcomes following IST or alloHSCT. However, in the non-alloHSCT setting, HPA, such as HLA-B*14:02, increase clonal evolution. In pediatric-onset AA, HPA increase the risk of somatic HLA loss, whereas in adult-onset AA, HPA are also associated with an increased risk of MDS-associated clonal evolution.

Our findings shed light on autoimmunity in AA by showing that there are several groups of AA risk alleles characterized by distinct peptide-binding motifs, with each group likely presenting a distinct autopeptide or its close variations — presumably derived from shared AA autoantigen(s). High-risk alleles differ in their peptide repertoires from lower risk alleles, suggesting that differences in allele pathogenicity in AA are due to autopeptide specificity. We speculate that this could occur if certain autopeptides contribute less to thymic negative selection of autoreactive T cells or are similar to viral epitopes causing cross-reactivity. While HPA increase the risk of developing AA, our results suggest that once a break in immune tolerance occurs and autoimmunity ensues, AA severity and responses to IST and alloHSCT do not differ based on the type of risk allele responsible for AA autoantigen presentation.

Importantly, our study shows that patients with HPA have an increased risk of clonal evolution, which is particularly high in adult-onset HPA patients. The significantly higher incidence (Table 1 and Table 2) of MDS-associated abnormalities in adult compared with pediatric patients could be explained by preexisting age-related clonal hematopoiesis in adults (34, 35). Additionally, we speculate that HPA may have a more insidious role in AA by sustaining occult autoimmune attack on HSPCs over time and may contribute to a longer “prodromal” period of occult autoimmune attack preceding AA diagnosis in some adult-onset patients (36). We also speculate that ethnicity-based differences in AA risk alleles and allele groups (e.g., HLA-B*14:02, an allele most closely linked to adverse clonal evolution, which is nearly absent in East Asia) likely contribute to population-based differences in AA incidence and may also contribute to differences in rates of post-AA secondary MDS.

Our study has limitations. Our HLA NGS analysis can reliably identify somatic mutations down to approximately 2% variant allele fraction. While we may have missed very tiny clones, we increased the specificity of detecting pathogenic mutations in true risk alleles by focusing on more significant clonal expansions of HLA-lacking cells. While we could not estimate AA pathogenicity for rare HLA alleles, our study included a diverse cohort of 505 patients with AA, allowing us to estimate pathogenicity for alleles in >60% of Asian and Black, >70% of Hispanic and Native American, and >85% of White North Americans. Our outcomes analysis may have failed to detect small differences in relapse, graft failure, and pediatric MDS due to insufficient cohort size. However, we evaluated 2 large independent multi-institutional cohorts, performing the first analysis to our knowledge of the role of HPA in alloHSCT outcomes in AA. Finally, while the number of patients with MDS-associated evolution was small, particularly among those with adult-onset AA, the effects of HPA and HLA-B*14:02 were statistically robust, were in agreement with our previous observations (10), and were supported by recent findings for HLA-B*14:02 from an independent cohort of patients from the NIH (15).

In conclusion, our study provides insights into the immune pathogenesis of AA, which will inform future efforts of autoantigen identification in AA and could be adapted to other autoimmune diseases. Increased MDS-associated clonal evolution in adult-onset AA patients with HPA should be confirmed in larger studies, but our results suggest that this group of patients may benefit from surveillance for long-term MDS-associated clonal evolution after IST.

## Methods

Methods are summarized in Figure 1, with details provided in Supplemental Methods.

### Patients with AA

AA was diagnosed using standard criteria (37–40). Severity of AA was defined according to Camitta Criteria (41). The HLA risk allele discovery cohort was assembled from 2 independent cohorts of patients with AA (Figure 1 and Supplemental Table 1). The 156 patients in the NAPAAC cohort included 98 AA patients of any AA severity from the Penn-CHOP Bone Marrow Failure Syndrome cohort (66 of whom were included in our previous study, ref. 10) and 58 patients recruited from other NAPAAC centers. Because the 156-patient NAPAAC cohort was used for both risk allele discovery and subsequent clinical outcomes analysis, it included consecutively enrolled patients. The second (CIBMTR-discovery) cohort consisted of 349 patients with AA participating in the CIBMTR research database. Patients for the CIBMTR-discovery cohort were selected to maximize the probability of identifying previously undiscovered risk alleles by analyzing patients who did not carry the 2 already known, most common AA risk alleles, HLA-B*14:02 and HLA-B*40:02 (9, 10). This preselection of patients for the CIBMTR-discovery cohort did not bias the subsequent HLA association or alloHSCT outcomes analysis, because those analyses were performed in separate, independent cohorts of patients (see Figure 1). The alloHSCT outcomes analysis was performed using an independent CIBMTR-outcomes cohort of 484 CIBMTR AA patients who received an MRD or 8/8 MUD alloHSCT between 1988 and 2018. A separate cohort of NMDP patients, identified based on patient donor search activity of the NMDP registry, were used for HLA association analyses. In accordance with the American Academy of Pediatrics, pediatric-onset AA was defined as the diagnosis of AA up to the age of 21 years (42).

### Somatic HLA loss

Somatic HLA loss was identified in the discovery cohort using a combination of targeted NGS of the *HLA-A*, -*B*, and -*C* genes to identify somatic mutations and SNP-A genotyping to identify acquired 6pCN-LOH, performed on peripheral blood or bone marrow DNA, as described (10, 43). Every HLA variant was manually curated in Integrative Genomics Viewer; most variants were additionally independently verified using Twin (Omixon) software (10, 43, 44). Due to limited quantities of hematopoietic cell DNA for some patients, 17 patients were only analyzed by HLA NGS but not SNP-A; in these patients, acquired 6pCN-LOH was analyzed using read depth imbalance across the sequenced *HLA-A*, -*B*, and -*C* alleles. A total of 14 patients were only analyzed for 6pCN-LOH by SNP-A but did not have sufficient DNA quantity for HLA NGS.

### Peptide-binding analyses

The amino acid residues comprising the peptide-binding pockets of HLA-A/B proteins, which determine HLA peptide repertoires, were previously established by crystallographic analyses (21, 45–48). The residues considered to form the “B pocket” were 7, 9, 24, 34, 45, 63, 66, 67, 70, and 99, and those for the “F pocket” were 74, 77, 80, 81, 84, 95, 97, 114, 116, 123, 133, 143, 146, and 147 (21). The peptide-binding motifs for AA HLA risk and non-risk alleles were based on previously published, experimentally obtained immunopeptidomes from HLA class I monoallelic cell lines (23). Peptides eluted from cell lines expressing a single HLA class I allele were downloaded for each allele of interest (23), except HLA-B*14:01, HLA-B*41:02, and HLA-B*50:02, for which immunopeptidome data were not available. Logo plots were then generated using the ggseqlogo R package (49).

### HLA pathogenicity rating

Relative pathogenicity of specific HLA class I alleles in AA was determined by the mutation frequency in analyzed AA patients carrying those alleles. Mutation frequencies were compared by Fisher’s exact tests.

### Statistics

#### HLA association.

Association analysis of HLA class I alleles with AA was performed in 6,323 patients with AA who searched the NMDP registry and presumed healthy controls (*n* = 31,057 to 50,000 per racial and ethnic group, as shown in Figure 1 and Supplemental Table 4), randomly selected from the unrelated donor registry after matching for sex, age, and ethnicity. The ORs and 95% CIs were calculated for each HLA variant within each ethnic group. Multiple-testing adjustment was performed using the FDR method with the adjusted *P* value at <5% FDR threshold considered significant (24).

#### Clinical outcomes.

The effect of HPA on IST outcomes was evaluated in a retrospective 156-patient NAPAAC cohort by comparing outcomes of patients with and without HPA using Fisher’s exact tests. The effect of HPA on transplant outcomes was evaluated in the CIBMTR-outcomes cohort using multivariate Cox proportional hazards analysis adjusting for age, Karnofsky performance status, donor type, and conditioning.

For all analyses, 2-tailed *P* values less than 0.05 were considered significant.

### Study approval

Patients were recruited with Institutional Review Board approval of respective NAPAAC institutions. CIBMTR/NMDP studies were approved by NMDP IRB overseeing CIBMTR research protocols.

## Author contributions

TSO and DVB conceptualized the study and obtained study funding. TSO oversaw human participant regulatory aspects, established multi-institutional NAPAAC study collaboration, and reviewed and contributed primary clinical outcomes data for Children’s Hospital of Philadelphia (CHOP) patients and due to these contributions is listed first as the shared co–first author with BFF. BFF analyzed the HLA NGS and SNP-A data and performed computational analyses of peptide-binding repertoires. JLD, IP, and HMX performed bioinformatic analysis of HLA NGS. MD and LG performed HLA association analysis in the NMDP data set. YBS performed clinical outcomes analysis in patients treated with IST (NAPAAC). ZDP, JG, EF, TAN, AS, and AAB contributed invaluable patient samples and AA expertise and reviewed and contributed primary clinical data for the NAPAAC sites. DF and AD performed HLA NGS sequencing of all samples. SK, YX, and MH performed statistical analysis of alloHSCT outcomes in CIBMTR patients. SZ, SN, PL, and CX performed experiments to determine functional effects of missense HLA variants. JHO contributed samples for HLA NGS and analyzed MDS-associated mutations for Penn-CHOP patients. BMC contributed invaluable expertise to HLA structural and peptide repertoire analysis. DSM established and oversaw HLA NGS at CHOP Immunogenetics Laboratory. SMG, SGEM, SP, SJL, YB and SRS provided invaluable samples from the CIBMTR cohort, assisted with regulatory aspects, and provided valuable guidance on design and analysis of CIBMTR patient outcomes. DVB oversaw all aspects of study, analyzed and interpreted all study data, and wrote the manuscript. All authors contributed to manuscript revisions and agree with the final version of the manuscript. TSO is listed before BFF, because TSO contributed to the study concept, funding, and patient recruitment.

## Supplementary Material

Supplemental data

## Figures and Tables

**Table 3:**
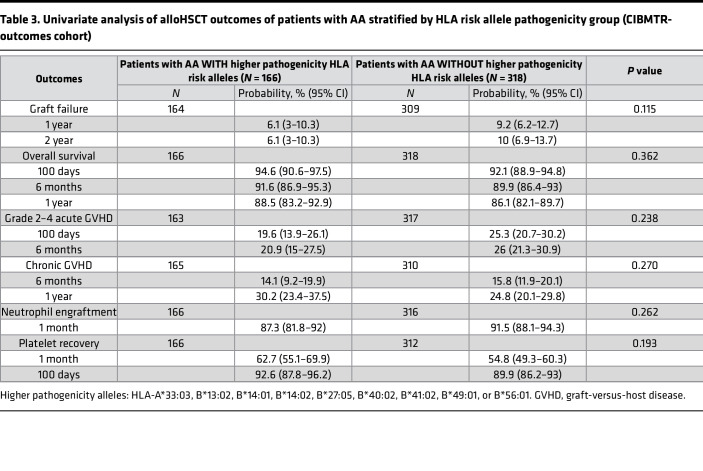
Univariate analysis of alloHSCT outcomes of patients with AA stratified by HLA risk allele pathogenicity group (CIBMTR-outcomes cohort)

**Table 2:**
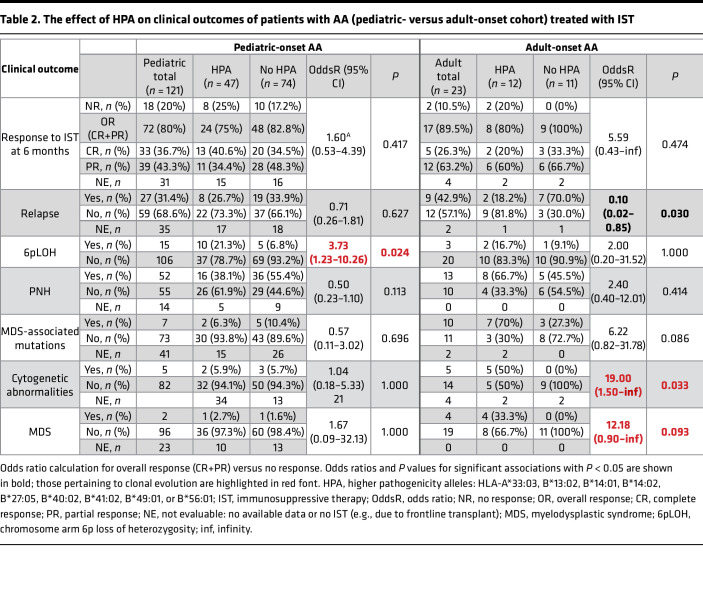
The effect of HPA on clinical outcomes of patients with AA (pediatric- versus adult-onset cohort) treated with IST

**Table 1:**
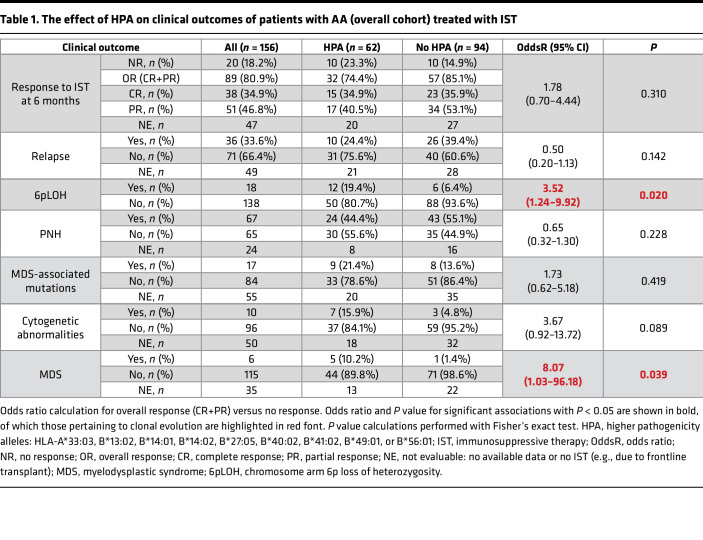
The effect of HPA on clinical outcomes of patients with AA (overall cohort) treated with IST

## Version 1 10/11/2022

In-Press Preview

## Version 2 11/22/2022

Electronic publication
