# Supplementary material for: Pathogenicity and impact of HLA class I alleles in aplastic anemia patients of different ethnicities
Source: JCI Insight. 2022 Nov 22;7(22):e163040. doi: 10.1172/jci.insight.163040 (PMC9746824; doi:10.1172/jci.insight.163040)
Supplement: Supplemental data [file jciinsight-7-163040-s223.pdf]

Supplemental Information For:

Pathogenicity and Clinical Impact of HLA Class I Alleles in Acquired Aplastic Anemia Patients of Different Ethnicities

Authors: Timothy S. Olson[1,2]*, Benjamin F. Frost[2,3]*, Jamie L. Duke[4], Marian Dribus[5], Hongbo M. Xie[6], Zachary D. Prudowsky[7,8], Elissa Furutani[9], Jonas Gudera[9,10], Yash B. Shah[1,2,11], Deborah Ferriola[4], Amalia Dinou[4], Ioanna Pagkrati[4], Soyoung Kim[12,13], Eric Xu[12], Meilun He[14], Shannon Zheng[3], Sally Nijim[3], Ping Lin[3], Chong Xu[15,16], Taizo A. Nakano[17], Joseph H. Oved[1,18], Beatriz M. Carreno[15,16], Yung-Tsi Bolon[14], Shahinaz M. Gadalla[19], Steven G.E. Marsh[20], Sophie Paczesny[21], Stephanie J Lee[12,22], Dimitrios S. Monos[4,23], Akiko Shimamura[9], Alison A. Bertuch[7,8], Loren Gragert[5], Stephen R Spellman[14], Daria V. Babushok[1,3]

Affiliations:
[1] Comprehensive Bone Marrow Failure Center, Children's Hospital of Philadelphia, Philadelphia, PA, USA;
[2] Division of Oncology, Department of Pediatrics, Children's Hospital of Philadelphia, Philadelphia, PA, USA;
[3] Division of Hematology-Oncology, Department of Medicine, University of Pennsylvania, Philadelphia, PA, USA;
[4] Department of Pathology and Laboratory Medicine, Children's Hospital of Philadelphia, Philadelphia, PA, USA;
[5] Department of Pathology and Laboratory Medicine, Tulane University School of Medicine, New Orleans, LA, USA;
[6] Department of Biomedical and Health Informatics, Children's Hospital of Philadelphia, Philadelphia, PA, USA;
[7] Department of Pediatrics, Division of Hematology/Oncology, Baylor College of Medicine, Houston, TX, USA;
[8] Texas Children's Cancer and Hematology Centers, Houston, TX, USA;
[9] Dana Farber/Boston Children’s Cancer and Blood Disorders Center, Harvard Medical School, Boston, MA, USA;
[10] Department of Pediatrics, Dr. von Hauner Children’s Hospital, LMU Klinikum Munich, Munich, Germany;
[11] Sidney Kimmel Medical College, Thomas Jefferson University, Philadelphia, PA, USA;
[12] Center for International Blood and Marrow Transplant Research, Medical College of Wisconsin, Milwaukee, WI, USA;
[13] Division of Biostatistics, Medical College of Wisconsin, Milwaukee, WI, USA;
[14] Center for International Blood and Marrow Transplant Research, National Marrow Donor Program/Be The Match, Minneapolis, MN, USA;
[15] Department of Pathology and Laboratory Medicine, Hospital of the University of Pennsylvania, Philadelphia, PA, USA;
[16] Parker Institute for Cancer Immunotherapy and Center for Cellular Immunotherapies, University of Pennsylvania, Philadelphia, PA, USA;
[17] Center for Cancer and Blood Disorders, Children’s Hospital Colorado, Aurora, CO, USA;
[18] Department of Pediatric Transplant and Cell Therapy, Memorial Sloan Kettering Cancer Center, New York, NY, USA;
[19] Division of Cancer Epidemiology and Genetics, National Institutes of Health, National Cancer Institute, Clinical Genetics Branch, Rockville, MD, USA;
[20] Anthony Nolan Research Institute and University College London Cancer Institute, Royal Free Campus, London, United Kingdom;
[21] Department of Microbiology and Immunology, Medical University of South Carolina, Charleston, SC, USA;
[22] Clinical Research Division, Fred Hutchinson Cancer Research Center, Seattle, WA, USA;
[23] Department of Pathology and Laboratory Medicine, Perelman Schools of Medicine, University of Pennsylvania, Philadelphia, PA, USA;

* these authors contributed equally to this work

Corresponding Author:
Daria Babushok, M.D., Ph.D.
BRB II/III Room 808
421 Curie Blvd
Philadelphia, PA 19104, USA
Email: daria.babushok@pennmedicine.upenn.edu
Phone: 215-573-0667, Fax: 215-615-5888

Table of Contents

Supplemental Methods 5

AA HLA risk allele discovery patients (NAPAAC and CIBMTR-discovery cohorts) 5

Identification of somatic HLA alterations 6

PNH flow cytometry 6

Generation of constructs containing HLA missense mutations. 7

Generation of monoallelic hematopoietic cell lines 8

Peptide-binding pocket analysis for AA risk and non-risk HLA alleles 9

Peptide-binding motifs of AA HLA risk and non-risk alleles 9

HLA allele association analysis (NMDP-association cohort) 10

AA pathogenicity stratification of HLA class I alleles 10

Higher Pathogenicity Alleles (HPA) for clinical outcomes analyses 11

Analysis of clinical outcomes in immunosuppressive therapy (IST)-treated patients (NAPAAC cohort) 11

CIBMTR alloHSCT Outcomes Analysis (CIBMTR-outcomes and CIBMTR-haplo cohorts) 12

Supplemental Figures 14

Supplemental Figure S1. Signal peptide sequence alignment for HLA-A and B genes 14

Supplemental Figure S2. DNA chromatographs for constructs with missense HLA mutations ... 15

Supplemental Figure S3. The complete pathogenicity stratification of 18 HLA class I risk alleles with identified somatic mutations and other alleles analyzed in ≥20 patients. 16

Supplemental Figure S4. The AA patient cohort for CIBMTR all HSCT clinical outcomes analysis (CIBMTR-outcomes cohort) 17

Supplemental Tables 18

Supplemental Table S1. Demographics of AA HLA risk allele discovery cohort 18

Supplemental Table S2. Acquired genetic alterations in HLA class I genes in the Discovery Cohort 19

Supplemental Table S3: Rates of Pathogenic vs. Bystander Roles for High, High-Intermediate, Low-Intermediate, and Low-Risk HLA class I Alleles. 25

Supplemental Table S3A: Individual Risk Allele Contribution to Autoimmune Recognition in Patients with AA with Multiple Risk Alleles. 25

Supplemental Table S3B Summary Statistics for Rates of Pathogenic vs. Bystander Roles for High, High-Intermediate, Low-Intermediate, and Low-Risk HLA class I Alleles in AA. 26

Supplemental Table S4. Demographic Characteristics of the NMDP-Association Cohort. 27

Supplemental Table S5: Association analysis of HLA risk and non-risk alleles with AA in NMDP-Association Populations of Different Ethnicities 28

Supplemental Table S5A. Association analysis of HLA risk and non-risk alleles with AA in NMDP-Association Cohort (White, cases n= 3,950, controls n=49,908) 28

Supplemental Table S5B. Association analysis of HLA risk and non-risk alleles with AA in NMDP-Association Cohort (Black, cases n= 1,030, controls n=50,000) 29
Supplemental Table S5C. Association analysis of HLA risk and non-risk alleles with AA in NMDP-Association Cohort (Hispanic, cases n= 840, controls n=50,000) 30
Supplemental Table S5D. Association analysis of HLA risk and non-risk alleles with AA in NMDP-Association Cohort (Asian and Pacific Islander, cases n= 463, controls n=50,000) 31
Supplemental Table S5E. Association analysis of HLA risk and non-risk alleles with AA in NMDP-Association Cohort (Native American, cases n= 40, controls n=31,057) 32
Supplemental Table S6 Association Analysis for All Evaluated HLA Class I Alleles with AA Per Population 33
Supplemental Table S6A: Association of HLA class I alleles in Asian and Pacific Islander NMDP Population 33
Supplemental Table S6B: Association of HLA class I alleles in Black NMDP Population 34
Supplemental Table S6B: Association of HLA class I alleles in Hispanic NMDP Population 36
Supplemental Table S6D: Association of HLA class I alleles in Native American NMDP Population 39
Supplemental Table S6E: Association of HLA class I alleles in White NMDP Population 39
Supplemental Table 7. Association Analysis of AA with HLA allele groups based on peptide-binding pocket structure 42
Supplemental Table 7A. Groups of AA Risk-Like HLA Class I Alleles Based on Peptide-Binding Pocket Identity Used in Group-Based Association Analysis 42
Supplemental Table 7B. Groups of Non-Risk-Like HLA Class I Alleles Based on Peptide-Binding Pocket Identity Used in Group-Based Association Analysis 43
Supplemental Table 7C. List of Other Alleles Analyzed in the Other HLA-A and Other HLA-B Allele Groups 44
Supplemental Table 7D. Association Analysis of AA with HLA allele groups in Asian and Pacific Islander and Black NMDP Populations 46
Supplemental Table 7E. Association Analysis of AA with HLA allele groups in Hispanic and Native American NMDP Populations 47
Supplemental Table 7F. Association Analysis of AA with HLA allele groups in White NMDP Population 49
Supplemental Table S8. Clinical characteristics of AA patients with and without Higher Pathogenicity Alleles (HPA). 50
Supplemental Table S9: Clinical characteristics of pediatric and adult AA patients with and without Higher Pathogenicity Alleles (HPA) 51
Supplemental Table S10. Characteristics of AA patients in CIBMTR-Outcomes Cohort 52
Supplemental Table S11: Multivariate analysis of alloHSCT outcomes of AA patients with and without Higher Pathogenicity Alleles (HPA). 55

Supplemental Table S12. AA patient characteristics for exploratory analysis of the effect of Higher Pathogenicity Allele concordance in donor-recipient pairs on outcomes of haploidentical alloSCT for AA. **57**

Supplemental Table S13: Univariate analysis of the effects of High Pathogenicity Alleles (HPA) concordance in donor-recipient pairs on outcomes after haploidentical alloSCT for AA. **60**

Supplemental Table S14: AA patient characteristics for exploratory analysis of the effect of HLA risk allele concordance in donor-recipient pairs on outcomes after haploidentical alloSCT for AA. **61**

Supplemental Table S15: Univariate analysis of the effects of HLA risk allele concordance in donor-recipient pairs on outcomes after haploidentical alloSCT for AA. **64**

Supplemental References **65**

Supplemental Methods

AA HLA risk allele discovery patients (NAPAAC and CIBMTR-discovery cohorts)

The HLA risk allele discovery population was assembled from two independent cohorts of AA patients (Figure 1, Supplemental Table S1). The 156 patients in the first ("NAPAAC") cohort included 58 AA patients recruited from NAPAAC and 98 AA patients from the Penn-CHOP Bone Marrow Failure Syndrome (BMFS) cohort (66 of whom were included in our previous study[1]), enrolled with the Institutional Review Board (IRB) approval at each participating institution with written informed consent received prior to participation. This 156-patient NA-PAAC cohort, was used for both risk allele discovery and clinical outcomes analysis, and for this reason included consecutively enrolled patients and was not pre-selected for any alleles. Samples used for mutation analysis were collected based on availability, and ranged in time of collection from diagnostic specimens to those collected following completion of IST. The diagnosis of AA was established using standard criteria, which required the exclusion of congenital bone marrow failure syndromes or other conditions mimicking AA[2,3]. Severity of AA was defined according to the Camitta Criteria[4]; patients with AA of any severity were included in the NAPAAC cohort. The second ("CIBMTR-discovery") cohort consisted of 349 patients with idiopathic AA and severe AA (SAA) without a known etiology who received an allogeneic hematopoietic stem cell transplant for AA between 2000-2018 and were registered with the CIBMTR with recipient data samples available in the biorepository. To increase the probability of patients having acquired HLA mutations, we selected patients who received immuno-suppression therapy before transplant. Because our goal was the discovery of novel HLA risk alleles, patients with the two most common AA HLA risk alleles, *HLA-B**14:02 and *HLA-B**40:02, were excluded from the CIBMTR-discovery cohort. This pre-selection of patients for the CIBMTR-Discovery cohort did not bias the subsequent HLA association or AlloHSCT outcomes analysis, because those analyses were performed in independent cohorts of patients that were not pre-selected for alleles or prior therapy. NAPAAC and CIBMTR patients were screened for potential overlap between the registries by comparing high-resolution HLA genotypes and

patient demographics: one duplicated enrollment was identified; that patient was analyzed in the NAPAAC cohort. In accordance with the American Academy of Pediatrics, pediatric-onset AA was defined as the diagnosis of AA up to the age of 21 years[5].

Identification of somatic HLA alterations

Somatic loss of HLA alleles through acquired 6p CN-LOH was identified by SNP-A genotyping of peripheral blood or bone marrow DNA as previously described[1,6]. Targeted next-generation sequencing (NGS) of the *HLA-A*, *B*, and *C* genes in the peripheral blood or bone marrow DNA was performed at >10,000X depth as previously described[7]. Briefly, sequence alignment and high-resolution HLA genotype determination were done with NGS-engine (GenDx Utrecht, Netherlands) and Twin (Omixon) at the CHOP CLIA-approved Immunogenetics Laboratory. Variant calling was done with GATK Haplotype Caller[8], with every variant manually curated in Integrative Genomics Viewer (IGV); most variants were independently verified using Twin (Omixon) software[1,6,7]. Due to limited quantities of hematopoietic cell DNA, 17 patients were only analyzed by HLA NGS; in these patients, acquired 6p CN-LOH was still able to be assessed through read depth imbalance across the sequenced HLA-A, B, and C alleles. Fourteen patients were analyzed for 6p CN-LOH by SNP-A genotyping but did not have sufficient DNA quantity for HLA NGS.

PNH flow cytometry

The presence of PNH clones was detected using multicolor flow cytometry, as previously described[9]. Briefly, PNH flow cytometry was performed as part of routine clinical care by CLIA-certified laboratories. PNH clones identified after June 2018 employed high resolution PNH flow cytometry with a sensitivity threshold of 0.05% for PNH granulocytes, with PNH granulocyte clones >0.05% considered positive, while testing performed prior to June 2018, used a sensitivity threshold of >1% granulocytes, with >1% granulocytes considered positive.

Generation of constructs containing HLA missense mutations.

Five missense mutations located in alpha-1 or alpha-2 domains were tested for effect on surface HLA expression (Figure 2E). cDNAs for HLA risk alleles were a kind gift from Dr. Catherine Wu (Harvard Medical School, Boston MA)[10] or purchased from the International Histocompatibility Working Group (IHWG) or Genscript. HLA mutations of interest were introduced by site-directed using the QuikChange II XL Site-Directed Mutagenesis kit (Agilent, Santa Clara, CA). Custom-designed oligonucleotide forward and reverse primers containing the missense mutations of interest were obtained from Integrated DNA Technologies (IDT, Coralville, IA) (sequences available upon request). For each mutated allele of interest, a sample reaction was assembled containing 5 μL of 10X reaction buffer, 10 ng of plasmid DNA, 125 ng of each custom-designed primer, 1 μL of dNTP mix, 3 μL of QuikSolution, and 1 μL of *PfuUltra* HF DNA polymerase. The sample and control reactions underwent a series of thermal cycling steps that allowed for primer annealing and mutant strand extension across the length of the plasmid. The cycling temperature profile was as follows: 1 minute at 95C; 18 cycles of 50 seconds at 95°C, 50 seconds at 60°C, 1 minute/kb of plasmid length at 68°C (6 minutes and 30 seconds for the pcDNA 3.1 plasmid-containing alleles and 5 minutes for the pCR2.1 plasmid-containing alleles), and 7 minutes at 68°C. Once the mutant strand was synthesized, 1 μL of the *Dpn* I restriction enzyme was added to digest the parental methylated un-mutagenized plasmid DNA to isolate the mutagenized plasmid. Subsequently, the mutagenized plasmid was transformed into XL10-Gold Ultracompetent cells alongside the aforementioned control plasmids used for the mutagenesis steps. Following transformation, colonies were picked and grown before extraction of the mutagenized plasmid from cells using the QIAprep Miniprep kit (Qiagen, Germantown, MD). Isolated plasmid DNA was sent for sequencing for validation of mutant constructs using the sequencing laboratories at the University of Pennsylvania (Supplemental Figure S2).

Wild-type and their respective mutant alleles were then cloned into a lentiviral vector expressing β2-microglobulin linked to the HLA allele of interest, referred to as the single-chain dimer, under the control of CMV promoter, as previously described[11]. For cloning, mutagenized and wildtype HLA alleles of interest were PCR-amplified

using the Phusion High-Fidelity Polymerase (Thermo Scientific, Waltham, MA). The 50 µL reaction mixture contained 25 µL of the 2X Phusion Master Mix, 25 moles of the forward and reverse primers, and 10 ng of the mutagenized plasmid DNA. The following 3-step cycling protocol was used: 30 seconds at 98°C; 35 cycles of 10 seconds at 98°C, 30 seconds at 58°C, 30 seconds/kb at 72°C; 10 minutes at 72°C. The PCR product was subsequently purified using the ZYMO Research (Tustin, CA) DNA Clean and Concentrator kit. In preparation for the upcoming ligation step, both the pTRE lentiviral vector and the allele inserts were cut with restriction enzymes SalI-HF and BamHI. The cut vector backbone and allele inserts were extracted from the gel using the QIAquick Gel Extraction kit (Qiagen, Germantown, MD). Ligation was performed using the Promega T4 DNA Ligase (Promega, Madison, WI), and a 3:1 insert to vector ratio was used. A 20 µL reaction containing 100 ng of vector DNA, 17 ng of insert DNA, 1 µL of Ligase 10X Buffer, and 1 µL of T4 DNA Ligase was assembled. The ligation reaction included two negative controls without inserts that respectively contained or did not include ligase to assess for a background of the undigested or re-ligated vector. The ligation reaction was incubated at room temperature for 3 hours. After purifying the ligation product using the ZYMO Research DNA Clean and Concentrator kit, each ligation product was transformed into NEB Stable competent *E. coli* cells using the cell-specific protocol. Evidence of a successful ligation was observed, with significant colony growth compared to the three relatively colony-deficient negative control plates (containing the two negative ligation controls and a negative transformation control). Cloned lentiviral constructs were validated by restriction digest as well as Sanger sequencing using custom-designed primers that were sequenced across the backbone/insert junctions.

Generation of monoallelic hematopoietic cell lines

K562 hematopoietic leukemia line was purchased from the ATCC (catalog CCL-243; ATCC, Manassas, VA). Because K562 cells express endogenous HLA under differentiating conditions or interferon-γ stimulation[12]; we used CRISPR/Cas9 technology to generate true HLA-lacking K562 line with no expression of endogenous HLA alleles. CRISPR/Cas9 plasmids (pSpCas9 BB-2A-GFP (PX458) vector) containing gRNA targeting B2M gene (B2M CRISPR guide RNA 2: GAGTAGCGCGAGCACAGCTA) was synthesized by Genscript USA Inc. 1 x10^6

K562 cells were transfected with 2 µg of gRNA-containing plasmid using the SF Cell Line 4D-Nucleofector X Kit (Lonza, cat# V4XC2012) and FF-100 program in the Amaxa 4D Nucleofector (Lonza, USA). 24 h post-transfection, single GFP-positive cells were flow-cytometrically sorted into 96 well plates, and individual clones were expanded. Single-cell clones were screened for successful B2M knockout after 24-hour pre-treatment of clones with 500 U/mL Interferon-gamma (Sigma-Aldrich 11040596001) by flow cytometry using FITC Mouse Anti-Human HLA-ABC (BD Biosciences, Clone G46-2.6 (RUO), catalog # 555552) to identify clones with loss of surface HLA for 24 hours to induce endogenous HLA expression. B2M knockout K562 cells were transduced with HLA-containing plasmids that co-expressed GFP. GFP-positive cells were then screened for surface HLA expression with PE-conjugated anti-HLA-ABC antibody (BD Biosciences, Clone G46-2.6 (RUO), catalog # 555553). Three replicate experiments analyzing surface expression were performed for each evaluated mutant allele. Statistical analysis was performed by comparing % surface expression normalized to the respective wild type alleles, using Student's t-test with Welch correction; p value <0.05 was considered significant.

Peptide-binding pocket analysis for AA risk and non-risk HLA alleles

The amino acid residues comprising the peptide-binding pockets of HLA class I A and B alleles were previously established based on crystallographic analyses[13-17]. The residues considered to form the "B pocket" were 7, 9, 24, 34, 45, 63, 66, 67, 70, and 99, and those for the "F pocket" were 74, 77, 80, 81, 84, 95, 97, 114, 116, 123, 133, 143, 146, and 147[17]. The HLA allele supertype assignment and allele-specific alignments of B and F pocket residues were as described[17], with a manual assignment of risk allele pocket similarity groups based on peptide-binding pocket identity.

Peptide-binding motifs of AA HLA risk and non-risk alleles

The peptide-binding motifs for AA HLA risk and non-risk alleles were based on experimentally obtained immunopeptidomes from HLA class I monoallelic cell lines[10]. Peptides eluted from cell lines expressing a single HLA Class I allele were downloaded for each allele of interest[10]. Immunopeptidome data were not available for *HLA-B**14:01, *B**41:02, and *B**50:02. Logoplots were then generated using the ggseqlogo R package[18].

HLA allele association analysis (NMDP-association cohort)

An association analysis of HLA Class I alleles with AA was performed using an independent validation cohort of AA patients registered with the National Marrow Donor Program (NMDP) ("NMDP association cohort"). The AA patient population was comprised of White (n=3979), Black (n= 1030), Hispanic (n=841), Asian and Pacific Islander (n=463), and Native American (n=40) AA patients, with race and ethnicity being self-reported. The control population was healthy controls (n=31,057 to 50,000 per racial/ethnic group), who were randomly selected from the unrelated donor registry after matching for sex, age, and ethnicity. HLA genotypes were determined by a combination of DNA-based methods and HLA genotype multiple imputation analysis for both cases and controls, as previously described for other HLA association studies[19,20]. Briefly, each subject's HLA typing was analyzed to determine all possible high-resolution 6-locus (A~C~B~DRB3/4/5~DRB1~DQB1) phased haplotypes. The likelihood of each possible genotype was then estimated based on published population haplotype frequencies[21]. For association analysis, five replicate associations were performed with potentially different haplotype assignments for each subject, and multiple imputation results were merged as described[19,20].

Association analysis was performed for the 19 identified risk alleles, and 7 identified non-risk alleles, and groups of alleles sharing the same peptide-binding pocket structures with the risk and non-risk alleles, based on previously published alignments[10] (Supplemental Tables S5-S7). Alleles that were not among the risk and non-risk allele-like groups were grouped as "Other HLA-A" or "Other HLA-B" alleles (Supplemental Table S7C), and analyzed separately (Supplemental Table S7). The Odds Ratios (OR) were calculated for each HLA variant. Two-tailed p-values <0.05 were considered significant. Multiple testing adjustment was performed using the False Discovery Rate (FDR) method with a 5% FDR threshold[22].

AA pathogenicity stratification of HLA class I alleles

We systematically compared mutation frequencies for 161 distinct HLA class I alleles (45 *HLA-A*, 75 *HLA-B*, and 40 *HLA-C*) from AA patients in the discovery cohort. Because a single inactivating event in a homozygous allele does not induce allele loss, the analysis was performed only for heterozygous alleles. Eighteen risk alleles

with identified somatic mutations and all HLA alleles present in 20 or more patients in the Discovery Cohort were included in this analysis. The rates of mutations per patient for each allele were compared to other alleles using two-tailed Fisher's exact tests, with a significant p-value <0.05 (Figure 3C). Five pathogenicity groups (High, High Intermediate, Low Intermediate, Low, and Non-Risk) were defined based on relative mutation frequencies and similarities and differences from other alleles (see text and Figure 3 caption for details).

Higher Pathogenicity Alleles (HPA) for clinical outcomes analyses

Higher Pathogenicity Alleles (HPA) were stringently defined as HLA alleles in the High or High-Intermediate Risk groups (Figure 2C), with additional requirements to increase the confidence of HPA group assignment. This was done because some risk alleles were analyzed in only a small number of patients. HPA alleles were thus required to either have had more than one patient with somatic mutations in the discovery cohort or were required to share one of the canonical risk peptide-binding pockets (Figure 3). Using these criteria, 9 alleles (*HLA-A*33:03*, *HLA-B*13:02*, *HLA-B*14:01*, *HLA-B*14:02*, *HLA-B*27:05*, *HLA-B*40:02*, *HLA-B*41:02*, *HLA-B*49:01*, *HLA-B*56:01)* were categorized as HPA. Patients without HPA were analyzed in the Lower Pathogenicity Allele (LPA) group.

In an analysis of haploidentical alloHSCT outcomes, outcomes of patients whose donors were matched or mis-matched for HPA or for all risk alleles were compared. For this analysis, patients who did not have any of the 19 identified risk alleles and patients whose donors were matched for some but not for all risk alleles were excluded.

Analysis of clinical outcomes in immunosuppressive therapy (IST)-treated patients (NAPAAC cohort)

Baseline characteristics (including age, AA severity, marrow cellularity, and acquired genetic alterations at diagnosis), treatment regimens, and response outcomes were obtained by clinical record review. Clinical endpoints were response to IST at 6 mo (defined as complete response (CR) if a patient met all 3 criteria of absolute neutrophil count (ANC)>1000, hemoglobin (Hgb) > 10g/dl, and platelets (Plt) $>100*10^3/\mu L$; no response (NR) if a patient continued to meet severe AA (SAA) criteria, and partial response (PR), if a patient improved over SAA criteria but did not meet CR). Relapse was defined as a clinically significant blood count decline requiring a

change of therapy; the patients were determined to have had a relapse as per the clinical documentation. Other clinical outcomes included the use of second-line therapies, clonal evolution metrics, including cytogenetic abnormalities, myelodysplastic syndrome (MDS)-associated somatic mutations, and MDS transformation, death, and last known clinical status. MDS-associated somatic mutations were identified by somatic NGS panel sequencing of hematologic-malignancy associated genes performed as a part of routine clinical care by CLIA-approved clinical laboratories as per standard of care in each respective NAPAAC institution; only mutations clinically reported as disease-associated were included in the analysis, and were analyzed as a binary outcome (present/absent). MDS transformation was determined using World Health Organization (WHO) classification criteria. Baseline characteristics and clinical outcomes were compared between patients carrying Higher Pathogenicity Alleles (HPA) and those without HPA using a two-tailed Fisher's exact test with a significant p-value <0.05. Odds ratios (ORs) were estimated using the Haldane-Anscombe correction.

CIBMTR alloHSCT Outcomes Analysis (CIBMTR-outcomes and CIBMTR-haplo cohorts)

The 484-patient cohort used for the analysis of allogeneic hematopoietic stem cell transplant (alloHSCT) outcomes included SAA patients participating in the CIBMTR (a research collaboration between the NMDP and Medical College of Wisconsin) research database, who underwent a matched sibling donor (MSD) or 8/8-matched unrelated donor (MUD) alloHSCT between 1988 and 2018 (Supplemental Figure S3). Patients were excluded if their marrow failure was caused by an inherited or other defined non-immune-mediated condition; if they received a syngeneic, haploidentical, mismatched unrelated or cord blood stem cell graft; or if they received a preparative regimen that is no longer considered standard for AA. Only patients with available high-resolution HLA typing and adequate follow-up data were included. Haploidentical alloHSCT outcomes were evaluated in a separate exploratory analysis (Supplemental Tables S12-S15). HLA typing was performed as previously described[23,24]. The National Marrow Donor Program IRB approved the study.

The primary outcome was graft failure (GF), analyzed as a single outcome combining primary and secondary GF. Primary GF was defined as failure to achieve absolute neutrophil count (ANC) of 0.5 x 109/L or a donor chimerism <5% in any compartment, including T-cell, unsorted blood, or marrow. Secondary GF was defined as initial engraftment followed by graft loss evidenced by a sustained drop in the neutrophil count to less than 0.5 x 109/L, or loss of donor chimerism to <5% in any compartment, or a second infusion within the first year after transplant in patients with documented clinical remission. Patients stably engrafted with complete or mixed chimerism were censored at 12 months. Secondary outcomes were overall survival (OS), neutrophil and platelet engraftment, and acute and chronic graft-versus-host disease (GVHD), all defined per CIBMTR definitions. Death was considered a competing risk for all outcomes. Multivariate analysis was performed with SAS (v9.4) using Cox proportional hazards model, with adjustment through stratification for patient age, Karnofsky Performance Status (KPS), donor type, and conditioning regimen. A raw two-tailed significance level of < 0.05 was used for the primary outcome and < 0.025 – for secondary outcomes.

Supplemental Figures

Supplemental Figure S1. Signal peptide sequence alignment for HLA-A and B genes

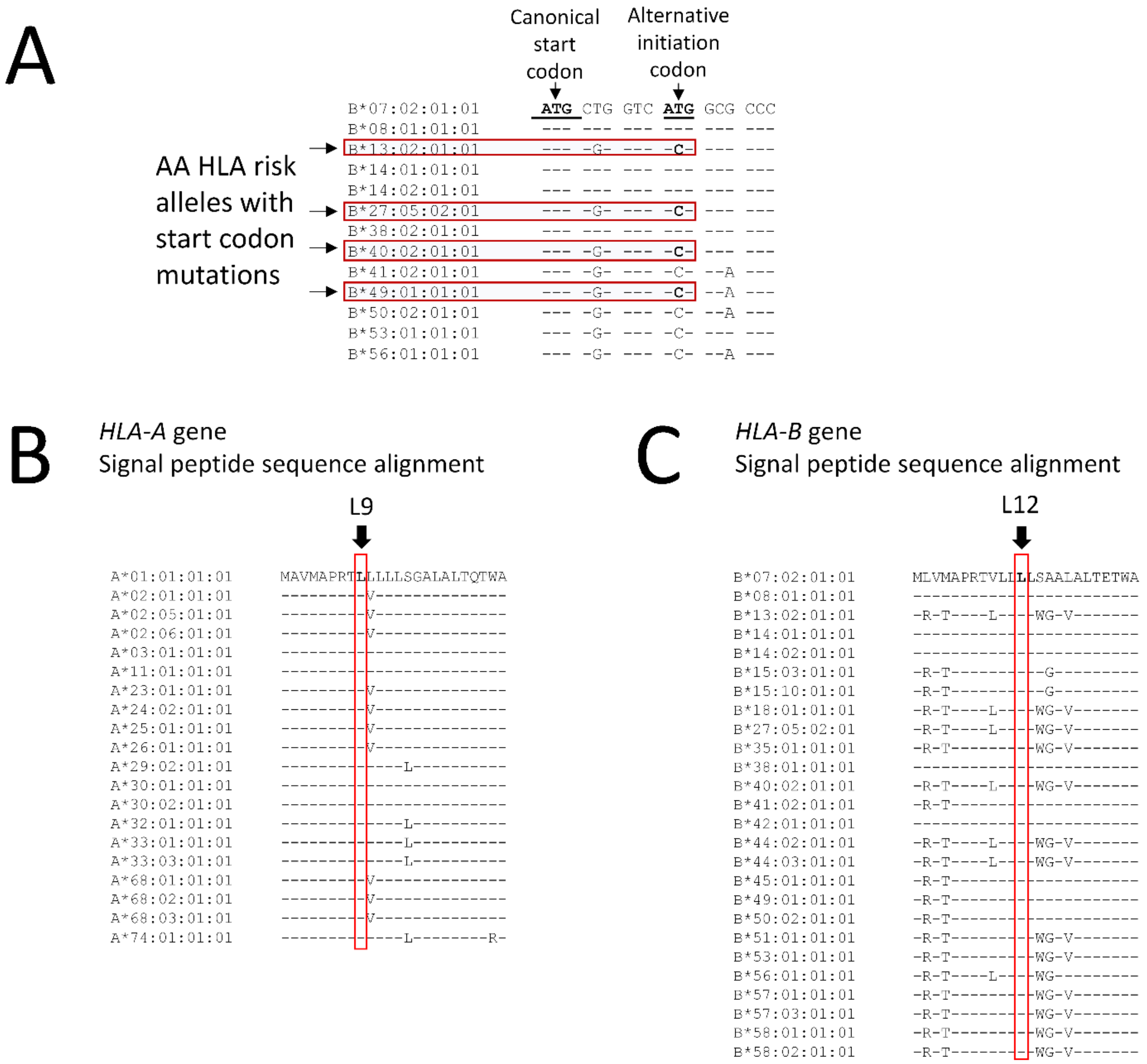

Signal peptide sequence alignment for HLA-A and B genes. A. Amino acid alignment of the N-terminal region of the identified HLA-B risk alleles. All four alleles which had mutations eliminating the start codon (arrow/red outline) have a polymorphism which eliminates the alternative start at the 4th position. B. Alignment of the N-terminal amino acids of the most common HLA-A alleles in the North American population, demonstrating a high degree of conservation of the L9 residue, which was found to be mutated in the study. C. Alignment of the N-terminal amino acids of the most common HLA-B alleles in the North American population, demonstrating a high degree of conservation of the L12 residue, which was found to be mutated in the study.

Supplemental Figure S2. DNA chromatographs for constructs with missense HLA mutations

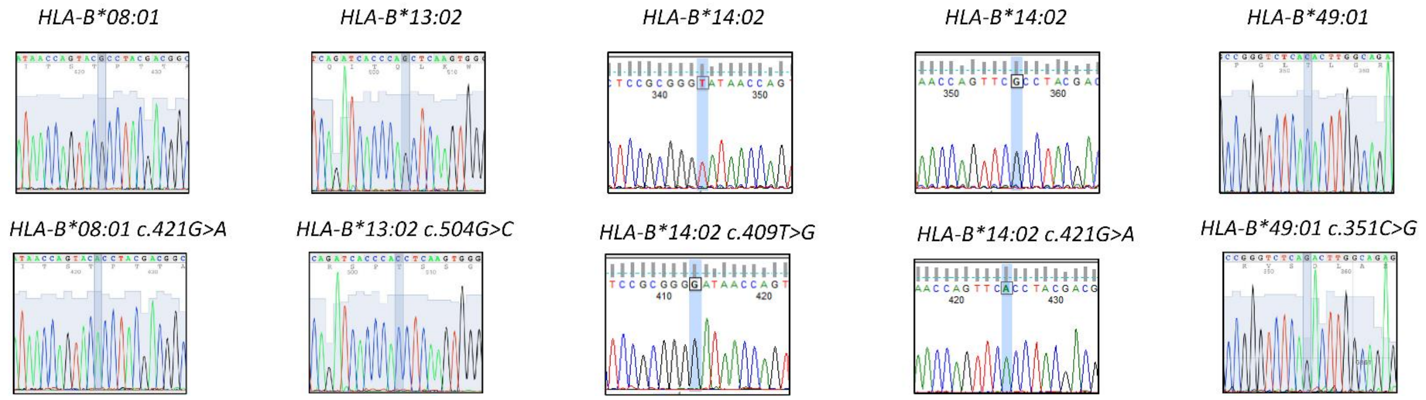

DNA chromatographs for constructs with missense HLA mutations. The chromatographs of the wild-type alleles are shown in the top row, with the corresponding sequence of alleles carrying the missense mutations shown below each wild-type allele (blue line).

Supplemental Figure S3. The complete pathogenicity stratification of 18 HLA class I risk alleles with identified somatic mutations and other alleles analyzed in ≥20 patients.

	Indeterminate		High						High-Intermediate				Low-intermediate		
Allele	B*50:02	B*38:02	B*14:02	B*14:01	B*40:02	A*33:03	B*49:01	B*41:02	B*56:01	A*74:01	B*27:05	B*13:02	B*53:01	B*08:01	A*68:01
cases/ total	1/1	1/3	9/25	2/9	2/9	3/15	4/23	1/6	1/7	1/9	2/19	2/28	1/22	4/94	1/25
patients with mutations	100.0%	33.3%	36.0%	22.2%	22.2%	20.0%	17.4%	16.7%	14.3%	11.1%	10.5%	7.1%	4.5%	4.3%	4.0%
mutations per patient	4.0	1.0	1.8	1.0	4.0	3.0	1.8	3.0	1.0	1.0	4.0	2.0	3.0	1.8	1.0
B*50:02		>0.999	0.385	0.300	0.300	0.250	0.208	0.286	0.250	0.200	0.150	0.103	0.087	0.053	0.077
B*38:02	>0.999		>0.999	>0.999	>0.999	>0.999	0.489	>0.999	>0.999	0.455	0.371	0.271	0.230	0.148	0.206
B*14:02	0.385	>0.999		0.682	0.682	0.477	0.200	0.634	0.387	0.225	0.081	0.016	0.012	<0.001	0.011
B*14:01	0.300	>0.999	0.682		>0.999	>0.999	>0.999	>0.999	>0.999	>0.999	0.574	0.244	0.195	0.085	0.164
B*40:02	0.300	>0.999	0.682	>0.999		>0.999	>0.999	>0.999	>0.999	>0.999	0.574	0.244	0.195	0.085	0.164
A*33:03	0.250	>0.999	0.477	>0.999	>0.999		>0.999	>0.999	>0.999	>0.999	0.634	0.324	0.283	0.053	0.139
B*49:01	0.208	0.489	0.200	>0.999	>0.999	>0.999		>0.999	>0.999	>0.999	0.673	0.390	0.346	0.047	0.180
B*41:02	0.286	>0.999	0.634	>0.999	>0.999	>0.999	>0.999		>0.999	>0.999	>0.999	0.453	0.389	0.271	0.355
B*56:01	0.250	>0.999	0.387	>0.999	>0.999	>0.999	>0.999	>0.999		>0.999	>0.999	0.500	0.431	0.307	0.395
A*74:01	0.200	0.455	0.225	>0.999	>0.999	>0.999	>0.999	>0.999	>0.999		>0.999	>0.999	0.503	0.373	0.465
B*27:05	0.150	0.371	0.081	0.574	0.574	0.634	0.673	>0.999	>0.999	>0.999		>0.999	0.588	0.265	0.570
B*13:02	0.103	0.271	0.016	0.244	0.244	0.324	0.390	0.453	0.500	>0.999	>0.999		>0.999	0.620	>0.999
B*53:01	0.087	0.230	0.012	0.195	0.195	0.283	0.346	0.389	0.431	0.503	0.588	>0.999		>0.999	>0.999
B*08:01	0.053	0.148	<0.001	0.085	0.085	0.053	0.047	0.271	0.307	0.373	0.265	0.620	>0.999		>0.999
A*68:01	0.077	0.083	0.011	0.164	0.164	0.139	0.180	0.355	0.395	0.465	0.570	>0.999	>0.999	>0.999	
B*18:01	0.053	0.146	0.001	0.093	0.093	0.067	0.066	0.263	0.296	0.357	0.263	0.573	>0.999	>0.999	>0.999
A*02:01	0.031	0.090	<0.001	0.033	0.033	0.014	0.009	0.170	0.195	0.242	0.122	0.218	0.481	0.481	0.523
B*07:02	0.025	0.073	<0.001	0.025	0.025	0.010	0.007	0.138	0.159	0.198	0.091	0.164	0.401	0.409	0.438

	Low			Low and Non-Risk											
Allele	B*18:01	A*02:01	B*07:02	A*11:01	A*26:01	A*29:02	A*30:01	A*31:01	A*32:01	B*15:01	B*35:01	B*40:01	B*44:03	B*51:01	B*57:01
cases/ total	1/37	5/193	2/119	0/52	0/21	0/21	0/27	0/29	0/29	0/44	0/46	0/38	0/24	0/50	0/21
patients with mutations	2.7%	2.6%	1.7%	0.0%	0.0%	0.0%	0.0%	0.0%	0.0%	0.0%	0.0%	0.0%	0.0%	0.0%	0.0%
mutations per patient	1.0	1.6	1.0	n/a	n/a	n/a	n/a	n/a	n/a	n/a	n/a	n/a	n/a	n/a	n/a
B*50:02	0.053	0.031	0.025	0.019	0.046	0.046	0.036	0.033	0.033	0.022	0.021	0.026	0.040	0.020	0.046
B*38:02	0.146	0.090	0.073	0.055	0.125	0.125	0.100	0.094	0.094	0.064	0.061	0.073	0.111	0.057	0.125
B*14:02	0.001	<0.001	<0.001	<0.001	0.002	0.002	0.001	0.000	0.000	<0.001	<0.001	<0.001	0.002	<0.001	0.002
B*14:01	0.093	0.033	0.025	0.020	0.083	0.083	0.057	0.051	0.051	0.026	0.024	0.033	0.068	0.021	0.083
B*40:02	0.093	0.033	0.025	0.020	0.083	0.083	0.057	0.051	0.051	0.026	0.024	0.033	0.068	0.021	0.083
A*33:03	0.067	0.014	0.010	0.010	0.064	0.064	0.040	0.034	0.034	0.014	0.013	0.019	0.050	0.010	0.064
B*49:01	0.066	0.009	0.007	0.007	0.109	0.109	0.038	0.033	0.033	0.012	0.010	0.017	0.050	0.008	0.109
B*41:02	0.263	0.170	0.138	0.103	0.222	0.222	0.182	0.171	0.171	0.120	0.115	0.136	0.200	0.107	0.222
B*56:01	0.296	0.195	0.159	0.119	0.250	0.250	0.206	0.194	0.194	0.137	0.132	0.156	0.226	0.123	0.250
A*74:01	0.357	0.242	0.198	0.148	0.300	0.300	0.250	0.237	0.237	0.170	0.164	0.192	0.273	0.153	0.300
B*27:05	0.263	0.122	0.091	0.069	0.219	0.219	0.165	0.152	0.152	0.088	0.082	0.107	0.189	0.073	0.219
B*13:02	0.573	0.218	0.164	0.120	0.500	0.500	0.491	0.237	0.237	0.148	0.140	0.176	0.493	0.126	0.500
B*53:01	>0.999	0.481	0.401	0.297	>0.999	>0.999	0.449	0.431	0.431	0.333	0.324	0.367	0.478	0.306	>0.999
B*08:01	>0.999	0.481	0.409	0.297	>0.999	>0.999	0.574	0.572	0.572	0.306	0.303	0.324	0.581	0.298	>0.999
A*68:01	>0.999	0.523	0.438	0.325	>0.999	>0.999	0.481	0.463	0.463	0.362	0.352	0.397	>0.999	0.333	>0.999
B*18:01		>0.999	0.559	0.416	>0.999	>0.999	>0.999	>0.999	>0.999	0.457	0.446	0.493	>0.999	0.425	>0.999
A*02:01	>0.999		0.713	0.587	>0.999	>0.999	>0.999	>0.999	>0.999	0.587	0.586	0.594	>0.999	0.586	>0.999
B*07:02	0.559	0.713		>0.999	>0.999	>0.999	>0.999	>0.999	>0.999	>0.999	>0.999	>0.999	>0.999	>0.999	>0.999

	Low and Non-Risk								Non-Risk							
Allele	C*01:02	C*02:02	C*03:03	C*03:04	C*05:01	C*08:02	C*12:03	C*16:01	A*01:01	A*03:01	A*24:02	B*44:02	C*04:01	C*06:02	C*07:01	C*07:02
cases/ total	0/35	0/29	0/38	0/57	0/61	0/34	0/35	0/23	0/113	0/100	0/67	0/67	0/99	0/86	0/125	0/138
patients with mutations	0.0%	0.0%	0.0%	0.0%	0.0%	0.0%	0.0%	0.0%	0.0%	0.0%	0.0%	0.0%	0.0%	0.0%	0.0%	0.0%
mutations per patient	n/a	n/a	n/a	n/a	n/a	n/a	n/a	n/a	n/a	n/a	n/a	n/a	n/a	n/a	n/a	n/a
B*50:02	0.028	0.033	0.026	0.017	0.016	0.029	0.028	0.042	0.009	0.010	0.015	0.015	0.010	0.012	0.008	0.007
B*38:02	0.079	0.094	0.073	0.050	0.047	0.081	0.079	0.115	0.026	0.029	0.043	0.043	0.029	0.034	0.023	0.021
B*14:02	0.000	0.000	<0.001	<0.001	<0.001	0.000	0.000	0.002	<0.001	<0.001	<0.001	<0.001	<0.001	<0.001	<0.001	<0.001
B*14:01	0.038	0.051	0.033	0.017	0.015	0.040	0.038	0.073	0.005	0.006	0.013	0.013	0.006	0.008	0.004	0.003
B*40:02	0.038	0.051	0.033	0.017	0.015	0.040	0.038	0.073	0.005	0.006	0.013	0.013	0.006	0.008	0.004	0.003
A*33:03	0.023	0.034	0.019	0.008	0.007	0.025	0.023	0.054	0.001	0.002	0.005	0.005	0.002	0.003	0.001	0.001
B*49:01	0.021	0.033	0.017	0.006	0.005	0.022	0.021	0.109	0.001	0.001	0.004	0.004	0.001	0.002	0.001	0.000
B*41:02	0.146	0.171	0.136	0.095	0.090	0.150	0.146	0.207	0.050	0.057	0.082	0.082	0.057	0.065	0.046	0.042
B*56:01	0.167	0.194	0.156	0.109	0.103	0.171	0.167	0.233	0.058	0.065	0.095	0.095	0.066	0.075	0.053	0.048
A*74:01	0.205	0.237	0.192	0.136	0.129	0.209	0.205	0.281	0.074	0.083	0.118	0.118	0.083	0.095	0.067	0.061
B*27:05	0.120	0.152	0.107	0.060	0.054	0.124	0.120	0.199	0.020	0.024	0.047	0.047	0.025	0.031	0.017	0.014
B*13:02	0.194	0.237	0.176	0.106	0.097	0.200	0.194	0.495	0.038	0.047	0.085	0.085	0.047	0.059	0.033	0.028
B*53:01	0.194	0.431	0.367	0.279	0.265	0.393	0.386	0.489	0.163	0.180	0.247	0.247	0.182	0.204	0.150	0.138
B*08:01	0.574	0.572	0.324	0.298	0.154	0.573	0.574	0.584	0.041	0.053	0.142	0.142	0.054	0.122	0.033	0.026
A*68:01	0.417	0.463	0.397	0.305	0.291	0.424	0.417	>0.999	0.181	0.200	0.272	0.272	0.202	0.225	0.167	0.153
B*18:01	>0.999	>0.999	0.493	0.394	0.378	>0.999	>0.999	>0.999	0.247	0.270	0.356	0.356	0.272	0.301	0.228	0.211
A*02:01	>0.999	>0.999	0.594	0.591	0.342	>0.999	>0.999	>0.999	0.162	0.170	0.332	0.332	0.171	0.328	0.161	0.078
B*07:02	>0.999	>0.999	>0.999	>0.999	0.549	>0.999	>0.999	>0.999	0.498	0.502	0.537	0.537	0.502	0.511	0.237	0.213

Shown is a pairwise comparison matrix of relative mutation frequencies per individual HLA alleles. HLA alleles are listed along the x-axis, grouped by pathogenicity ranking. High, High-Intermediate, Low-Intermediate, and Low risk alleles are shown in decreasing intensity of Red, and Non-Risk alleles–in green. *B*38:02 and B*50:02* were insufficiently prevalent in the cohort for analysis—these were listed as Indeterminate. Twenty additional alleles with no identified mutations were analyzed in 20+ patients, providing sufficient statistical power to conclude that their pathogenicity is at most Low Risk; these are listed as "Low or Non-Risk". The number of patient cases with mutations, total patients with listed allele, % mutation frequency, and median number of mutations per patient are listed in the header rows below each allele. Pairwise comparison of mutation frequencies is shown a correlation matrix. Comparisons were performed by Fisher's exact tests, with p-value listed for each pair of alleles. P-values<0.05 are shaded in dark red for comparison odds ratios (OR)>1 or dark green for OR<1. Trends with p values ≥0.05 and <0.1 are shown in light red for OR>1 and light green for OR<1.

Supplemental Figure S4. The AA patient cohort for CIBMTR all HSCT clinical outcomes analysis (CIBMTR-outcomes cohort)

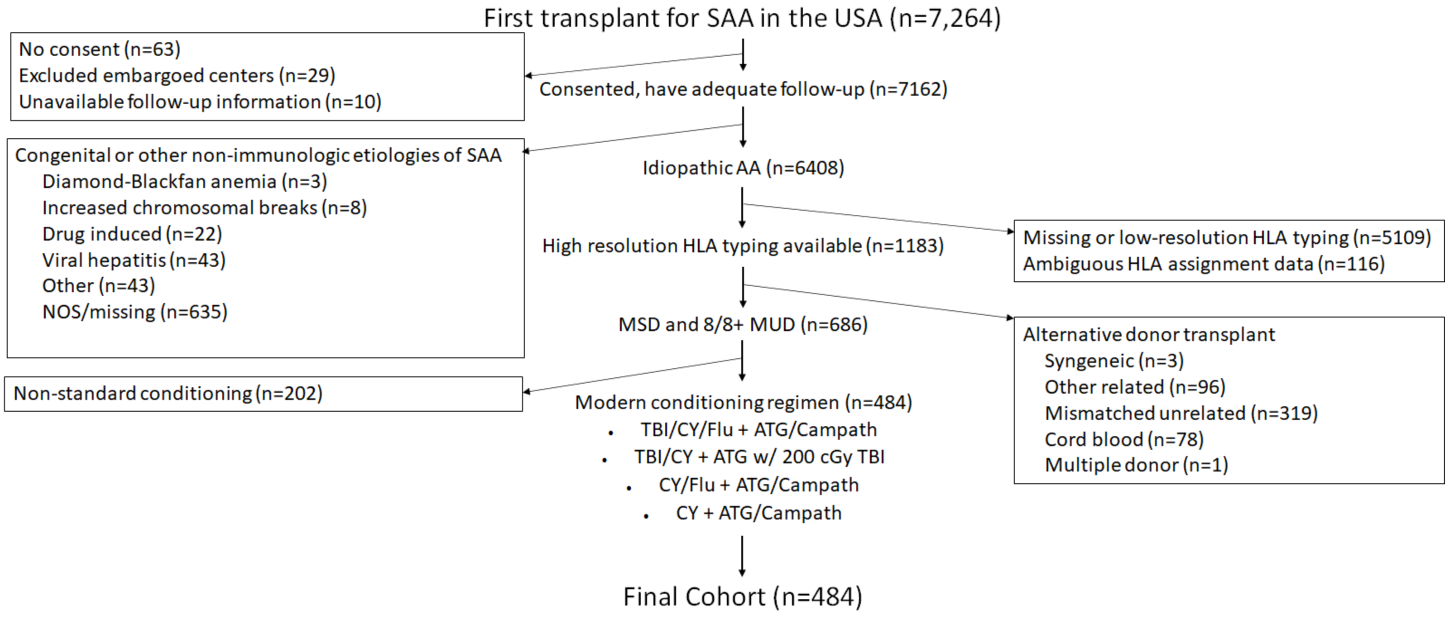

The AA patient cohort for CIBMTR alloHSCT clinical outcomes analysis. A schematic diagram showing the CIBMTR-outcomes cohort composition, with the relevant inclusion and exclusion criteria that resulted in the final 484 patient cohort.

Supplemental Tables

Supplemental Table S1. Demographics of AA HLA risk allele discovery cohort

**Category**	CIBMTR (n=349)	NAPAAC (n=156)	p-value
**Age**			
Age at diagnosis, years (median, range)	19.9 (1.4-71.0)	10.7 (1.5-65.6)	
Pediatric-onset, n (%)	195 (55.9%)	121 (84.0%)	<0.001
Adult-onset, n (%)	154 (44.1%)	23 (16.0%)	
N/A	0	12	
**Sex**			
Male, n (%)	184 (52.7%)	88 (56.4%)	0.499
Female, n (%)	165 (47.3%)	68 (43.6%)	
N/A	0	0	
**Race**			
Asian, n (%)	20 (6.0%)	6 (4.3%)	0.185
Black, n (%)	40 (11.9%)	22 (15.7%)	
White, n (%)	272 (81.0%)	107 (76.4%)	
Interracial, n (%)	0 (0%)	5 (3.8%)	
Other, n (%)	4 (1.2%)	0 (0%)	
N/A	13	16	
**Ethnicity**			
Hispanic, n (%)	32 (9.5%)	19 (13.1%)	0.260
Not Hispanic, n(%)	304 (90.5%)	126 (86.9%)	
N/A	13	11	
**PNH clone**			
Positive, n (%)	43 (20.7%)	67 (50.8%)	<0.001
Negative, n (%)	165 (79.3%)	65 (49.2%)	
N/A	141	24	
**HLA Risk Allele**			
*HLA-B**14:02, n (%)	0* (0%)	26 (16.0%)	
*HLA-B**40:02, n (%)	0* (0%)	9 (5.8%)	N/A*

N/A, not available; PNH, paroxysmal nocturnal hemoglobinuria; CIBMTR, Center for International Blood and Marrow Transplant Research; NAPAAC, North American Pediatric Aplastic Anemia Consortium. * Patients with the two most common AA HLA risk alleles *HLA-B**14:02 and *B**40:02 were excluded from the CIBMTR-discovery cohort.

Supplemental Table S2. Acquired genetic alterations in HLA class I genes in the Discovery Cohort

Patient ID	6p CN-LOH clones (n)	6p CN-LOH Region	HLA Alleles Lost through CNLOH	Somatic Mutations (n)	Mutated Allele	Type of Mutation	Mutation (coding)	Mutation (protein)	Variant Allele Frequency (%)
CCI-000008	2	6pterp21.1<br>6pterp21.2	**A*02:01**, B*07:02, C*07:02	1	HLA-A*02:01	Frameshift	c.591delG	p.N198fs	11.6
BCH-01	2	6pterp12.1<br>6pterp21.32	**A*02:01**, B*44:02, C*05:09	1	HLA-A*02:01	Missense	c.848G>A	p.C283Y	14.6
ST-00150946	0	n/a		3	HLA-A*02:01	Frameshift	c.16delC	p.R7fs	2.2
					HLA-A*02:01	Nonsense	c.19C>T	p.R7*	9.2
					HLA-A*02:01	Splice site	c.616_620+2delACGGGT	p.?	3.4
ST-00197331	1	6pterp21.32	**A*02:01**, B*08:01, C*07:01	1	HLA-A*02:01	Missense	c.26T>C	p.L9P	50.1
ST-00208002	2	6pterp21.2<br>6pterp21.33	**A*02:01**, B*44:02, C*05:01	2	HLA-A*02:01	Splice site([25])	c.373T>A	p.C125S; p.?	10.7
					HLA-A*02:01	Splice site	c.618_620+6 delGGGTACCA	p.?	5.6
CHOP 281.01*	4	6pterp22.1<br>6pterp22.1<br>6pterp21.33<br>6pterp12.1	**A*33:03**, B*44:03, C*07:06	1	HLA-A*33:03	Nonsense	c.526C>G	p.Y142fs	15.1
ST-00201018	1	6pterp21.32	**A*33:03**, B*58:01, C*03:02	3	HLA-A*33:03	Frameshift	c.294_303delCCGAGTGGAC	p.R99fs	3.3
					HLA-A*33:03	Missense	c.698A>G	p.Y233C	3.1
					HLA-A*33:03	Missense	c.700C>T	p.P234S	7.6
ST-00204077	2	6pterp12.2<br>6pterp21.32	**A*33:03**, B*15:16, C*14:02	5	HLA-A*33:03	Frameshift	c.16delC	p.R7fs	7.6
					HLA-A*33:03	Nonsense	c.19C>T	p.R7*	6.4
					HLA-A*33:03	Nonsense	c.153C>A	p.Y51*	15.1
					HLA-A*33:03	Frameshift	c.811dupG	p.G271fs	25.4

					HLA-A*33:03	Missense	c.851A>C	p.H284P	6.3
CHOP 505.01*	2	6pterp12.1<br>6pterp21.31	^^	1	HLA-A*68:01	Frameshift	c.795delC	p.F265fs	3.5
ST-00207365	2	6pterp21.32<br>6pterp21.33	**A*74:01**, B*35:01, C*07:01	1	HLA-A*74:01	Nonsense	c.19C>T	p.R7*	2.7
ST-00190922	0	n/a		1	HLA-B*07:02	Nonsense	c.19C>T	p.R7*	11.0
ST-00201926	0	n/a		1	HLA-B*07:02	Nonsense	c.19C>T	p.R7*	2.5
CHOP 580.01	0	n/a		1	HLA-B*08:01	Nonsense	c.19C>T	p.R7*	8.6
ST-00140088	0	n/a		1	HLA-B*08:01	Nonsense	c.19C>T	p.R7*	2.0
ST-00144542	0	n/a		1	HLA-B*08:01	Nonsense	c.19C>T	p.R7*	1.9
ST-00190520	2	6pterp21.2	A*23:01, **B*08:01**, C*07:01	4	HLA-B*08:01	Nonsense	c.19C>T	p.R7*	33.1
					HLA-B*08:01	Frameshift	c.36_49delGCTCTCGGCGGCCC	p.L13fs	5.3
		6pterp21.32			HLA-B*08:01	Frameshift	c.44_53delCGGCCCTGGC	p.A16*	0.2
					HLA-B*08:01	Missense	c.421G>A	p.A141T	8.7
Baylor 23158	1	6pterp21.31	A*31:01; **B*13:02**, C*06:02	1	HLA-B*13:02	Nonsense	c.19C>T	p.R7*	7.0
ST-00153763	0	n/a		3	HLA-B*13:02	Start Loss	c.1A>T	p.M1?	5.9
					HLA-B*13:02	Nonsense	c.19C>T	p.R7*	31.6
					HLA-B*13:02	Missense	c.504G>C	p.Q168H	5.8
BCH-02	1	6pterp21.33	A*02:01, **B*14:01**, C*08:02	1	HLA-B*14:01	Nonsense	c.19C>T	p.R7*	12.9
ST-00158997	0	n/a		1	HLA-B*14:01	Frameshift	c.16delC	p.R7fs	53.7
CHOP 506.01*	0	n/a		1	HLA-B*14:02	Frameshift	c.890insAT	p.R297fs	17.0
CHOP 555.01	0	n/a		2	HLA-B*14:02	Frameshift	c.16delC	p.R7fs	1.0
					HLA-B*14:02	Nonsense	c.19C>T	p.R7*	32.0
CHOP 609.1	0	n/a		4	HLA-B*14:02	Nonsense	c.19C>T	p.R7*	12.2
					HLA-B*14:02	Missense	c.35T>A	p.L12Q	5.0

					HLA-B*14:02	Missense	c.409T>G	p.Y137D	2.5
					HLA-B*14:02	Missense	c.421G>A	p.A141T	9.6
CHOP 638.01	0	n/a		1	HLA-B*14:02	Nonsense	c.19C>T	p.R7*	1.7
Baylor 15481	0	n/a		2	HLA-B*14:02	Nonsense	c.19C>T	p.R7*	10.0
					HLA-B*14:02	Frameshift	c.417delG	p.Q139fs	3.8
BCH-03	0	n/a		1	HLA-B*14:02	Nonsense	c.19C>T	p.R7*	3.3
BCH-04	0	n/a		2	HLA-B*14:02	Nonsense	c.19C>T	p.R7*	19.7
					HLA-B*14:02	Nonsense	c.69G>A	p.W23*	41.1
CHOP 54.01*	2	6pterp21.31	A*33:01, **B*14:02**, C*08:02	2	HLA-B*14:02	Nonsense	c.19C>T	p.R7*	6.6
		6pterp21.1			HLA-B*14:02	Frameshift	c.880delT	p.L294fs	20.1
BCH-05	1	6pterp21.32	A*24:02, **B*14:02**, C*08:02	1	HLA-B*14:02	Nonsense	c.19C>T	p.R7*	0.7
ST-00147936	0	n/a		1	HLA-B*18:01	Nonsense	c.19C>T	p.R7*	3.2
ST-00191796	1	6pterp12.1	A*30:02, **B*27:05**, C*02:02	4	HLA-B*27:05	Frameshift	c.7_23delGGTCAC-GGCGCCCGAA	p.V3fs	1.3
					HLA-B*27:05	Frameshift	c.16delC	p.R7fs	6.9
					HLA-B*27:05	Nonsense	c.19C>T	p.R7*	3.7
					HLA-B*27:05	Frameshift	c.937delG	p.A313fs	15.6
BCH-06	0	n/a		4	HLA-B*27:05	Start Loss	c.1A>G	p.M1?	30.6
					HLA-B*27:05	Nonsense	c.19C>T	p.R7*	4.0
					HLA-B*27:05	Frameshift	c.46dupG	p.A16fs	9.9
					HLA-B*27:05	Missense	c.626G>A	p.P209Q	12.0
ST-00115171	0	n/a		1	HLA-B*38:02	Nonsense	c.19C>T	p.R7*	7.8
CHOP 29.01*	0	n/a		5	HLA-B*40:02	Start Loss	c.1A>G	p.M1?	7.0
					HLA-B*40:02	Nonsense	c.19C>T	p.R7*	12.5
					HLA-B*40:02	Nonsense	c.742C>T	p.Q248*	3.9
					HLA-B*40:02	Nonsense	c.862G>T	p.E288*	4.8

					HLA-B*40:02	Frameshift	c.870delG	p.L290fs	1.6
CHOP 435.01*	0	n/a		3	HLA-B*40:02	Frameshift	c.15delC	p.A5fs	17.3
					HLA-B*40:02	Frameshift	c.60insGA	p.T20fs	19.2
					HLA-B*40:02	Frameshift	c.286delAG	p.Q96fs	5.1
ST-00191967	1	6pterp12.1	A*29:02, **B*41:02**, C*17:03	3	HLA-B*41:02	Nonsense	c.19C>T	p.R7*	6.0
					HLA-B*41:02	Frameshift	c.116_119delCCGG	p.G40fs	25.4
					HLA-B*41:02	Del	c.124_147delGGG-GAGCCCCGCTTCATCACCGTG	p.G42_V49del	0.8
ST-00157774	0	n/a		1	HLA-B*49:01	Start Loss	c.1A>G	p.M1?	34.4
ST-00168320	0	n/a		3	HLA-B*49:01	Nonsense	c.19C>T	p.R7*	9.4
					HLA-B*49:01	Frameshift	c.565delG	p.V189fs	16.4
					HLA-B*49:01	Missense	c.842A>G	p. Y281C	16.2
ST-00192407	0	n/a		1	HLA-B*49:01	Start Loss	c.1A>T	p.M1?	6.9
ST-00207773	1	n/a	A*01:01, **B*49:01**, C*07:01	2	HLA-B*49:01	Nonsense	c.19C>T	p.R7*	3.9
					HLA-B*49:01	Missense	c.351C>G	p.H117Q	22.1
ST-00205927	1	6pterp12.1	A*02:01, **B*50:02**, C*06:02	4	HLA-B*50:02	Frameshift	c.16delC	p.R7fs	2.2
					HLA-B*50:02	Frameshift	c.115delC	p.P39fs	10.6
					HLA-B*50:02	Nonsense	c.232C>T	p.Q78*	5.1
					HLA-B*50:02	Missense	c.325T>A	p.Y109N	12.6
ST-00195220	2	6pterp21.1 6pterp21.32	A*68:02, **B*53:01**, C*04:01	3	HLA-B*53:01	Frameshift	c.16delC	p.R7fs	23.7
					HLA-B*53:01	Nonsense	c.19C>T	p.R7*	3.5

					HLA-B*53:01	Missense	c.694T>G	p.F232V	32.2
ST-00180757	0	n/a		1	HLA-B*56:01	Nonsense	c.19C>T	p.R7*	8.0
ST-00178922	1	6pterp21.32	**A*02:01†**	0	n/a				
ST-00145113	1	6pterp21.32	**A*02:06†**	0	n/a				
CHOP 56.01*	4	6pterp21.31<br>6pterp21.1<br>6pterp12.1<br>6pterp11.1	**B*14:02,** C*08:02‡	0	n/a				
CHOP 284.01*	2	6pterp21.32<br>6pterp11.1	**A*02:01,** B*35:03, C*12:03	0	n/a				
CHOP 348.01*	1	6pterp21.2	A*29:02, **B*13:02,** C*06:02	0	n/a				
CHOP 390.01*	1	6WC	^^	0	n/a				
CHOP 471.01*	4	6pterp21.32<br>6pterp21.31<br>6pterp21.2<br>6pterp12.1	A*11:01, B*35:01, C*04:01	0	n/a				
CHOP 564.01	1	6p	^^	0	n/a				
CHOP 575.01	1	6pterp21.31	^^	0	n/a				
Baylor 12533	1	6pterp21.2	A*31:01, **B*40:02**, C 03:04	0	n/a				
Baylor 14545	1	6pterp21.1	A*03:01, B*47:01, C*06:02	0	n/a				
BCH-07	1	6pterp21.32	A*03:01, **B*49:01**, C*07:01	0	n/a				
ST-00137635	1	6pterp21.32	^^	0	n/a				
ST-00140300	1	6pterp21.31	**A*02:01**, B*51:01, C*01:02	0	n/a				
ST-00150545	1	6pterp21.32	**A*02:01, B*08:01**, C*07:01	0	n/a				

ST-00160762	1	6pterp21.32	**A*68:01**, B*44:02, C*07:04	0	n/a
ST-00162664	2	6pterp21.32<br>6pterp21.33	^^	0	n/a
ST-00187774	2	6pterp21.1<br>6pterp21.32	**A*02:01, B*18:01‡**	0	n/a
ST-00191385	1	n/a	A*01:01, **B*49:01**, C*07:01	0	n/a
ST-00191682	1	n/a	**A*02:06,** B*48:01, C*08:01	0	n/a
ST-00202213	1	6pterp21.32	**A*02:01, B*07:02**, C*07:02	0	n/a
ST-00205930	1	6pterp21.2	A*26:01, B*38:01, C*12:03	0	n/a
ST-00207098	1	6pterp21.32	A*03:01, **B*07:02**, C*07:02	0	n/a
ST-00219193	2	6pterp21.2<br>6pterp21.32	**A*02:01, B*27:05**, C*01:02	0	n/a
ST-00285358	2	6pterp12.1<br>6pterp21.32	**A*02:01**, B*39:01, C*07:02	0	n/a
ST-00291066	1	6pterp21.31	**A*02:01**, B*57:01, C*06:02	0	n/a
ST-00291929	2	6pterp12.1<br>6pterp21.32	A*31:01, B*35:01, C*04:01	0	n/a
ST-00296246	2	6pterp21.1<br>6pterp21.31	**A*02:01**, B*15:01, C*03:03	0	n/a
ST-00300085	1	6pterp21.2	A*34:02, B*47:03, C*17:01	0	n/a

† Only HLA-A lost in 6pLOH event; ‡ Homozygous third allele; ^SNP-A not available, CN-LOH status determined from targeted HLA NGS; ^^ Lost haplotype could not be definitively determined due to small 6p CN-LOH clone size. In patients with CN-LOH events, known HLA risk alleles are shown in bold.
The data from this table are summarized in the main manuscript in the following figures: the summary of genetic mechanisms of HLA loss is presented in Figure 1A. The locations and number of mutations are shown in Figure 1B, with the breakdown of the mutation types summarized in Figure 1C. The breakdown of HLA alleles targeted by mutations are shown in Figure 3.

Supplemental Table S3: Rates of Pathogenic vs. Bystander Roles for High, High-Intermediate, Low-Intermediate, and Low-Risk HLA class I Alleles.

Supplemental Table S3A: Individual Risk Allele Contribution to Autoimmune Recognition in Patients with AA with Multiple Risk Alleles.

Patient ID	Number of Known Risk Alleles, n	Risk Allele 1	Pathogenicity Category 1	Risk Allele 2	Pathogenicity Category 2	Risk Allele 3	Pathogenicity Category 3	Risk Category of Pathogenic > Bystander Allele
ST-00191796	2	A*02:01	Low	**B*27:05**	**High-Int**			High-Int > Low
ST-00191967	2	B*08:01	Low-Int	**B*41:02**	**High**			High > Low-Int
ST-00192407	3	A*02:01	Low	A*02:01	Low	**B*49:01**	**High**	High > Low
ST-00153763	2	A*02:01	Low	**B*13:02**	**High-Int**			High-Int > Low
ST-00204077	2	**A*33:03**	**High**	B*53:01	Low-Int			High > Low-Int
ST-00157774	2	B*08:01	Low-Int	**B*49:01**	**High**			High > Low-Int
ST-00195220	2	A*74:01	High-Int	**B*53:01**	**Low-Int**			Low-Int > High-Int
ST-00197331	2	**A*02:01**	**Low**	B*08:01	Low-Int			Low > Low-Int
ST-00208002	2	**A*02:01**	**Low**	A*68:01	Low-Int			Low > Low-Int
ST-00205927	2	A*02:01	Low	**B*50:02**	**Inderminate**			Indeterminate > Low
ST-00168320	2	A*02:01	Low	**B*49:01**	**High**			High > Low
ST-00201926	2	A*02:01	Low	**B*07:02**	**Low**			Low > Low
ST-00140088	3	A*02:01	Low	B*07:02	Low	**B*08:01**	**Low-Int**	Low-Int > 2 Low-Risk alleles
ST-00150946	2	**A*02:01**	**Low**	B*07:02	Low			Low > Low
ST-00207773	2	**B*49:01**	**High**	B*56:01	High-Int			High > High-Int
BAYLOR 15481	2	**B*14:02**	**High**	B*41:02	High			High > High
BAYLOR 23158	2	A*02:01	Low	**B*13:02**	**High-Int**			High-Int > Low
BCH-02	2	A*02:01	Low	**B*14:01**	**High**			High > Low
CCI-000008	2	**A*02:01**	**Low**	B*07:02	Low			Low > Low
CHOP 029.01	3	A*02:01	Low	A*02:01	Low	**B*40:02**	**High**	High > Low
CHOP 435.01	2	A*02:01	Low	**B*40:02**	**High**			High > Low
CHOP 505.01	3	**A*68:01**	**Low-Int**	B*07:02	Low	B*14:02	High	Low-Int > High and Low
CHOP 506.01	2	A*02:01	Low	**B*14:02**	**High**			High > Low
CHOP 555.01	2	A*02:01	Low	**B*14:02**	**High**			High > Low
CHOP 609.1	2	B*08:01	Low-Int	**B*14:02**	**High**			High > Low-Int
CHOP 638.01	2	B*08:01	Low-Int	**B*14:02**	**High**			High > Low-Int
BCH-06	3	A*02:01	Low	A*02:01	Low	**B*27:05**	**High-Int**	High-Int > Low
BCH-05	2	B*08:01	Low-Int	**B*14:02**	**High**			High > Low-Int

The table lists patients with two or more risk alleles of defined pathogenicity categories, with the pathogenic risk allele (as identified by somatic HLA mutation) shown in bold with gray shading.

Supplemental Table S3B Summary Statistics for Rates of Pathogenic vs. Bystander Roles for High, High-Intermediate, Low-Intermediate, and Low-Risk HLA class I Alleles in AA.

Pathogenicity Category of Risk Allele	Number of Patients with 2 or more Risk Alleles from Different Pathogenicity Groups, n	Number of times allele was pathogenic, n	Number of times allele was bystander, n	Frequency of allele being pathogenic, %	Frequency of allele being bystander, %
**High**	13	12	1	92.31%	7.69%
**High-Int**	12	10	2	83.33%	16.67%
**Low-Int**	13	5	8	38.46%	61.54%
**Low**	23	2	21	8.70%	91.30%

The table shows summary statistics for data in Supplemental Table 3A.

Supplemental Table S4. Demographic Characteristics of the NMDP-Association Cohort.

NMDP Group	Asian and Pacific Islander		Black		Hispanic		Native American		White	
Cohort	Case	Control	Case	Control	Case	Control	Case	Control	Case	Control
Subjects, n	463	50,000	1,030	50,000	840	50,000	40	31,057	3,950	49,908
Age, median (range), years	20 (0-67)	21.5 (18-60)	17 (0-69)	22.5 (18-60)	14 (0-70)	21.5 (18-60)	12.5 (0-56)	32 (18-60)	20 (0-70)	21 (18-60)
Male / Female, %	49.5% / 50.5%	53% / 47%	53.8% / 46.1%	54% / 46%	59.4% / 40.6%	59% / 41%	40% / 60%	35% / 65%	53.5% / 46.4%	55% / 45%

Supplemental Table S5: Association analysis of HLA risk and non-risk alleles with AA in NMDP-Association Populations of Different Ethnicities

Supplemental Table S5A. Association analysis of HLA risk and non-risk alleles with AA in NMDP-Association Cohort (White, cases n= 3,950, controls n=49,908)

Risk Allele	Number of Alleles in Cases	Allele Frequency in Cases	Number of Alleles in Controls	Allele Frequency in Controls	OR (95% CI)	P value	P adj
A*02:01	2333	0.295	25732	0.258	1.216 (1.14 - 1.297)	**0.000**	**0.000**
A*02:06	27	0.003	377	0.004	0.836 (0.541 - 1.231)	0.405	1.000
A*33:03	53	0.007	654	0.007	1.023 (0.762 - 1.342)	0.739	1.000
A*68:01	261	0.033	3192	0.032	1.048 (0.917 - 1.193)	0.483	1.000
A*74:01	6	0.001	151	0.002	0.504 (0.199 - 1.042)	0.110	1.000
B*07:02	1121	0.142	11946	0.120	1.223 (1.135 - 1.316)	**0.000**	**0.000**
B*08:01	949	0.120	10289	0.103	1.140 (1.053 - 1.233)	**0.001**	**0.025**
B*13:02	212	0.027	2290	0.023	1.162 (1.001 - 1.341)	**0.044**	0.420
B*14:01	92	0.012	991	0.010	1.194 (0.956 - 1.473)	0.123	0.745
B*14:02	454	0.057	2847	0.029	2.045 (1.835 - 2.275)	**0.000**	**0.000**
B*18:01	313	0.040	4481	0.045	0.868 (0.768 - 0.978)	**0.022**	0.266
B*27:05	291	0.037	3384	0.034	1.089 (0.958 - 1.232)	0.192	0.836
B*38:02	0	0.000	110	0.001	0 (0 - 0)	0.887	0.966
B*40:02	225	0.029	1373	0.014	2.107 (1.816 - 2.433)	**0.000**	**0.000**
B*41:02	35	0.004	490	0.005	0.903 (0.628 - 1.254)	0.563	0.966
B*49:01	149	0.019	1883	0.019	1.007 (0.846 - 1.190)	0.937	0.971
B*50:02	3	0.000	48	0.000	0.787 (0.226 - 2.109)	0.361	0.966
B*53:01	38	0.005	721	0.007	0.639 (0.448 - 0.881)	**0.009**	0.169
B*56:01	44	0.006	575	0.006	0.968 (0.701 - 1.301)	0.834	0.966

Non-Risk Allele	Number of Alleles in Cases	Allele Frequency in Cases	Number of Alleles in Controls	Allele Frequency in Controls	OR (95% CI)	P value	P adj
A*01:01	1192	0.151	15336	0.154	0.956 (0.889 - 1.028)	0.229	1.000
A*03:01	1028	0.130	13064	0.131	0.992 (0.920 - 1.070)	0.843	1.000
A*11:01	453	0.057	6233	0.062	0.911 (0.821 - 1.009)	0.076	1.000
A*23:01	124	0.016	2272	0.023	0.685 (0.566 - 0.819)	**0.000**	**0.002**
A*24:02	621	0.079	8947	0.090	0.850 (0.776 - 0.930)	**0.000**	**0.014**
B*44:02	698	0.088	8518	0.085	1.047 (0.960 - 1.141)	0.296	0.966
B*44:03	248	0.031	4817	0.048	0.613 (0.534 - 0.700)	**0.000**	**0.000**

Risk allele: 19 AA risk alleles identified in this study; Non-Risk Alleles: 7 alleles of HLA-A and B genes significantly less likely to be targeted by mutations (A*01:01, A*03:01, A*24:02, B*44:03); or sharing the same peptide-binding pocket residues with a non-risk allele (A*11:01, A*23:01, B*44:02). Cntrl, control. OR, Odds Ratio. CI, confidence interval; P, P-value; Padj, P value adjusted for multiple comparisons. Statistically significant P-value (<0.05) are **bolded**. For statistically significant comparisons, OR >1 for risk alleles are colored in red, and for non-risk alleles OR <1 are colored in blue.

Supplemental Table S5B. Association analysis of HLA risk and non-risk alleles with AA in NMDP-Association Cohort (Black, cases n= 1,030, controls n=50,000)

Risk Allele	Number of Alleles in Cases	Allele Frequency in Cases	Number of Alleles in Controls	Allele Frequency in Controls	OR (95% CI)	P value	P adj
A*02:01	279	0.135	11811	0.118	1.225 (1.062 - 1.408)	**0.006**	0.368
A*02:06	2	0.001	114	0.001	1.024 (0.211 - 2.968)	0.749	1.000
A*33:03	101	0.049	5208	0.052	0.927 (0.746 - 1.138)	0.488	1.000
A*68:01	65	0.032	3705	0.037	0.83 (0.636 - 1.064)	0.176	1.000
A*74:01	116	0.056	5378	0.054	1.048 (0.855 - 1.272)	0.643	1.000
B*07:02	151	0.074	7303	0.073	1.02 (0.852 - 1.213)	0.772	1.000
B*08:01	94	0.046	3512	0.035	1.312 (1.048 - 1.622)	**0.015**	0.397
B*13:02	11	0.005	822	0.008	0.650 (0.335 - 1.122)	0.157	1.000
B*14:01	28	0.014	752	0.008	1.865 (1.247 - 2.673)	**0.003**	0.167
B*14:02	103	0.050	2211	0.022	2.400 (1.937 - 2.943)	**0.000**	**0.000**
B*18:01	74	0.036	3076	0.031	1.184 (0.923 - 1.494)	0.170	1.000
B*27:05	17	0.008	813	0.008	1.026 (0.608 - 1.611)	0.887	1.000
B*38:02	0	0.000	11	0.000	0.000 (0-7.431)	0.951	1.000
B*40:02	8	0.004	388	0.004	1.002 (0.455 - 1.887)	0.779	1.000
B*41:02	7	0.003	583	0.006	0.601 (0.261 - 1.164)	0.179	1.000
B*49:01	76	0.037	2922	0.029	1.253 (0.977 - 1.583)	0.066	0.775
B*50:02	2	0.001	9	0.000	10.007 (1.534 - 40.555)	**0.045**	0.652
B*53:01	273	0.133	11842	0.118	1.105 (0.955 - 1.274)	0.176	1.000
B*56:01	3	0.002	252	0.003	0.654 (0.18 - 1.632)	0.448	1.000
**Non-Risk Allele**	**Number of Alleles in Cases**	**Allele Frequency in Cases**	**Number of Alleles in Controls**	**Allele Frequency in Controls**	**OR (95% CI)**	**P value**	**P adj**
A*01:01	96	0.047	4289	0.043	1.072 (0.858 - 1.324)	0.527	1.000
A*03:01	136	0.066	8193	0.082	0.776 (0.642 - 0.931)	**0.007**	0.368
A*11:01	27	0.013	1294	0.013	0.984 (0.648 - 1.426)	0.936	1.000
A*23:01	197	0.096	10948	0.109	0.880 (0.749 - 1.029)	0.115	1.000
A*24:02	50	0.024	2554	0.026	0.937 (0.692 - 1.239)	0.663	1.000
B*44:02	85	0.041	4946	0.049	1.147 (0.810 - 1.572)	0.483	1.000
B*44:03	38	0.019	1598	0.016	0.801 (0.632 - 1.000)	0.063	0.775

Risk allele: 19 AA risk alleles identified in this study; Non-Risk Alleles: 7 alleles of HLA-A and B genes significantly less likely to be targeted by mutations (A*01:01, A*03:01, A*24:02, B*44:03); or sharing the same peptide-binding pocket residues with a non-risk allele (A*11:01, A*23:01, B*44:02). Cntrl, control. OR, Odds Ratio. CI, confidence interval; P, P-value; Padj, P value adjusted for multiple comparisons. Statistically significant P-value (<0.05) are **bolded**. For statistically significant comparisons, OR >1 for risk alleles are colored in red, and for non-risk alleles OR <1 are colored in blue.

Supplemental Table S5C. Association analysis of HLA risk and non-risk alleles with AA in NMDP-Association Cohort (Hispanic, cases n= 840, controls n=50,000)

Risk Allele	Number of Alleles in Cases	Allele Frequency in Cases	Number of Alleles in Controls	Allele Frequency in Controls	OR (95% CI)	P value	P adj
A*02:01	385	0.229	20588	0.206	1.163 (1.012 - 1.336)	**0.036**	0.679
A*02:06	97	0.058	3892	0.039	1.562 (1.249 - 1.929)	**0.000**	**0.035**
A*33:03	22	0.013	830	0.008	1.600 (1.011 - 2.395)	**0.037**	0.679
A*68:01	84	0.050	4804	0.048	1.025 (0.808 - 1.283)	0.640	1.000
A*74:01	10	0.006	683	0.007	0.873 (0.435 - 1.547)	0.672	1.000
B*07:02	115	0.068	5698	0.057	1.225 (0.996 - 1.492)	**0.049**	0.724
B*08:01	82	0.049	4287	0.043	1.152 (0.906 - 1.444)	0.234	1.000
B*13:02	25	0.015	1287	0.013	1.172 (0.764 - 1.712)	0.439	1.000
B*14:01	20	0.012	806	0.008	1.462 (0.899 - 2.234)	0.133	1.000
B*14:02	118	0.070	4236	0.042	1.632 (1.323 - 1.993)	**0.000**	**0.000**
B*18:01	58	0.035	4002	0.040	0.823 (0.618 - 1.073)	0.166	1.000
B*27:05	40	0.024	1877	0.019	1.307 (0.935 - 1.774)	0.102	1.000
B*38:02	15	0.009	1841	0.018	0 (0 - 0.225)	0.951	1.000
B*40:02	149	0.089	4829	0.048	1.919 (1.590 - 2.299)	**0.000**	**0.000**
B*41:02	8	0.005	567	0.006	0.843 (0.383 - 1.585)	0.641	1.000
B*49:01	43	0.026	2427	0.024	1.047 (0.754 - 1.412)	0.775	1.000
B*50:02	6	0.004	211	0.002	1.752 (0.703 - 3.577)	0.204	1.000
B*53:01	40	0.024	1610	0.016	1.394 (0.982 - 1.916)	0.051	0.724
B*56:01	10	0.006	440	0.004	1.364 (0.677 - 2.424)	0.335	1.000
**Non-Risk Allele**	**Number of Alleles in Cases**	**Allele Frequency in Cases**	**Number of Alleles in Controls**	**Allele Frequency in Controls**	**OR (95% CI)**	**P value**	**P adj**
A*01:01	109	0.065	7132	0.071	0.909 (0.737 - 1.111)	0.364	1.000
A*03:01	100	0.060	8086	0.081	0.703 (0.564 - 0.866)	**0.001**	0.131
A*11:01	63	0.037	4584	0.046	0.806 (0.614 - 1.037)	0.106	1.000
A*23:01	47	0.028	3270	0.033	0.821 (0.597 - 1.098)	0.204	1.000
A*24:02	195	0.116	12261	0.123	0.948 (0.802 - 1.115)	0.526	1.000
B*44:02	54	0.032	3551	0.036	0.927 (0.695 - 1.210)	0.586	1.000
B*44:03	65	0.039	5872	0.059	0.613 (0.467 - 0.789)	**0.000**	**0.022**

Risk allele: 19 AA risk alleles identified in this study; Non-Risk Alleles: 7 alleles of HLA-A and B genes significantly less likely to be targeted by mutations (A*01:01, A*03:01, A*24:02, B*44:03); or sharing the same peptide-binding pocket residues with a non-risk allele (A*11:01, A*23:01, B*44:02). Cntrl, control. OR, Odds Ratio. CI, confidence interval; P, P-value; Padj, P value adjusted for multiple comparisons. Statistically significant P-value (<0.05) are **bolded**. For statistically significant comparisons, OR >1 for risk alleles are colored in red, and for non-risk alleles OR <1 are colored in blue.

Supplemental Table S5D. Association analysis of HLA risk and non-risk alleles with AA in NMDP-Association Cohort (Asian and Pacific Islander, cases n= 463, controls n=50,000)

Risk Allele	Number of Alleles in Cases	Allele Frequency in Cases	Number of Alleles in Controls	Allele Frequency in Controls	OR (95% CI)	P value	P adj
A*02:01	92	0.100	8087	0.081	1.328 (1.047 - 1.667)	**0.025**	0.551
A*02:06	64	0.069	3835	0.038	1.930 (1.462 - 2.505)	**0.000**	**0.005**
A*33:03	65	0.070	10150	0.102	0.665 (0.504 - 0.861)	**0.003**	0.106
A*68:01	13	0.014	2666	0.027	0.490 (0.261 - 0.831)	**0.015**	0.376
A*74:01	4	0.004	155	0.002	2.119 (0.522 - 5.608)	0.199	1.000
B*07:02	28	0.030	2711	0.027	1.032 (0.672 - 1.511)	0.878	1.000
B*08:01	6	0.006	1738	0.017	0.315 (0.112 - 0.682)	**0.010**	0.171
B*13:02	22	0.024	1857	0.019	1.216 (0.753 - 1.848)	0.400	1.000
B*14:01	2	0.002	159	0.002	1.224 (0.187 - 4.023)	0.673	1.000
B*14:02	2	0.002	173	0.002	1.374 (0.257 - 4.124)	0.661	1.000
B*18:01	3	0.003	1391	0.014	0.233 (0.059 - 0.606)	**0.013**	0.177
B*27:05	10	0.010	725	0.007	1.450 (0.710 - 2.604)	0.294	1.000
B*38:02	22	0.023	3318	0.033	0.719 (0.452 - 1.080)	0.137	1.000
B*40:02	48	0.052	2228	0.022	2.536 (1.852 - 3.395)	**0.000**	**0.000**
B*41:02	0	0.000	33	0.000	0 (0 - 3.621)	0.966	1.000
B*49:01	6	0.006	299	0.003	2.203 (0.867 - 4.540)	0.057	0.496
B*50:02	0	0.000	3	0.000	7.215 (NA-1.27E+07)	0.774	1.000
B*53:01	2	0.002	88	0.001	2.461 (0.405 - 7.811)	0.209	1.000
B*56:01	6	0.006	648	0.006	1.001 (0.399 - 2.049)	0.756	1.000
Non-Risk Allele	Number of Alleles in Cases	Allele Frequency in Cases	Number of Alleles in Controls	Allele Frequency in Controls	OR (95% CI)	P value	P adj
A*01:01	41	0.044	6655	0.067	0.601 (0.420 - 0.832)	**0.003**	0.114
A*03:01	24	0.026	3125	0.031	0.849 (0.547 - 1.252)	0.446	1.000
A*11:01	161	0.174	18138	0.181	0.920 (0.752 - 1.119)	0.412	1.000
A*23:01	4	0.004	386	0.004	1.125 (0.347 - 2.647)	0.815	1.000
A*24:02	171	0.184	16216	0.162	1.208 (0.993 - 1.463)	0.058	1.000
B*44:02	26	0.028	4861	0.049	1.450 (0.691 - 2.648)	0.273	1.000
B*44:03	9	0.010	681	0.007	0.554 (0.360 - 0.811)	**0.004**	0.126

Risk allele: 19 AA risk alleles identified in this study; Non-Risk Alleles: 7 alleles of HLA-A and B genes significantly less likely to be targeted by mutations (A*01:01, A*03:01, A*24:02, B*44:03); or sharing the same peptide-binding pocket residues with a non-risk allele (A*11:01, A*23:01, B*44:02). Cntrl, control. OR, Odds Ratio. CI, confidence interval; P, P-value; Padj, P value adjusted for multiple comparisons. Statistically significant P-value (<0.05) are **bolded**. For statistically significant comparisons, OR >1 for risk alleles are colored in red, and for non-risk alleles OR <1 are colored in blue.

Supplemental Table S5E. Association analysis of HLA risk and non-risk alleles with AA in NMDP-Association Cohort (Native American, cases n=40, controls n=31,057)

Risk Allele	Number of Alleles in Cases	Allele Frequency in Cases	Number of Alleles in Controls	Allele Frequency in Controls	OR (95% CI)	P value	P adj
A*02:01	23	0.293	12206	0.197	2.039 (1.093 - 3.825)	**0.029**	1.000
A*02:06	7	0.093	1155	0.019	4.069 (1.439 - 9.369)	**0.010**	0.490
A*33:03	1	0.013	1054	0.017	0.747 (0.042 - 3.442)	0.774	1.000
A*68:01	1	0.010	2605	0.042	0.230 (0.013 - 1.19E+05)	0.371	1.000
A*74:01	0	0.000	618	0.010	0 (0 - 1.49E+09)	0.984	1.000
B*07:02	5	0.063	4666	0.075	0.665 (0.199 - 1.662)	0.439	1.000
B*08:01	2	0.025	3601	0.058	0.421 (0.068 - 1.372)	0.233	1.000
B*13:02	1	0.013	732	0.012	1.072 (0.060 - 4.938)	0.946	1.000
B*14:01	1	0.018	341	0.005	3.284 (0.386 - 12.624)	0.259	1.000
B*14:02	2	0.020	1670	0.027	0.747 (0.102 - 2.683)	0.735	1.000
B*18:01	3	0.038	2140	0.034	1.122 (0.271 - 3.104)	0.848	1.000
B*27:05	5	0.063	2045	0.033	2.110 (0.723 - 4.92)	0.119	1.000
B*38:02	0	0.000	27	0.000	0 (0 - 1.08E+11)	0.984	1.000
B*40:02	6	0.075	1938	0.031	2.746 (1.036 - 6.085)	**0.023**	1.000
B*41:02	0	0.000	389	0.006	0 (0 - 1.03E+06)	0.981	1.000
B*49:01	1	0.013	1153	0.019	0.674 (0.038 - 3.104)	0.697	1.000
B*50:02	0	0.000	45	0.001	0 (0 - 6.68E+07)	0.985	1.000
B*53:01	0	0.000	1202	0.019	0 (0 - 8.68E+04)	0.979	1.000
B*56:01	2	0.023	409	0.007	3.548 (0.535 - 12.140)	0.151	1.000

Non-Risk Allele	Number of Alleles in Cases	Allele Frequency in Cases	Number of Alleles in Controls	Allele Frequency in Controls	OR (95% CI)	P value	P adj
A*01:01	5	0.063	5575	0.090	0.549 (0.164 - 1.372)	0.256	1.000
A*03:01	9	0.113	5758	0.093	1.359 (0.609 - 2.740)	0.418	1.000
A*11:01	4	0.050	2824	0.045	1.144 (0.342 - 2.860)	0.798	1.000
A*23:01	1	0.013	2409	0.039	0.316 (0.018 - 1.455)	0.256	1.000
A*24:02	11	0.135	6996	0.113	1.222 (0.563 - 2.423)	0.592	1.000
B*44:02	6	0.075	2973	0.048	1.728 (0.653 - 3.828)	0.217	1.000
B*44:03	3	0.038	4480	0.072	0.510 (0.123 - 1.411)	0.262	1.000

Risk allele: 19 AA risk alleles identified in this study; Non-Risk Alleles: 7 alleles of HLA-A and B genes significantly less likely to be targeted by mutations (A*01:01, A*03:01, A*24:02, B*44:03); or sharing the same peptide-binding pocket residues with a non-risk allele (A*11:01, A*23:01, B*44:02). Cntrl, control. OR, Odds Ratio. CI, confidence interval; P, P-value; Padj, P value adjusted for multiple comparisons. Statistically significant P-value (<0.05) are **bolded**. For statistically significant comparisons, OR >1 for risk alleles are colored in red, and for non-risk alleles OR <1 are colored in blue.

Supplemental Table S6 Association Analysis for All Evaluated HLA Class I Alleles with AA Per Population

Supplemental Table S6A: Association of HLA class I alleles in Asian and Pacific Islander NMDP Population

HLA-A Allele	OR	LCI	UCI	P value	adjusted P value	adj LCI	adj UCI
A*26:03	5.845	2.756	10.844	**0.000**	**0.000**	2.069	16.515
A*02:06g	1.930	1.462	2.505	**0.000**	**0.000**	1.316	2.832
A*33:03g	0.665	0.504	0.861	**0.003**	**0.016**	0.463	0.954
A*01:01g	0.601	0.420	0.832	**0.003**	**0.016**	0.385	0.938
A*68:01g	0.490	0.261	0.831	**0.015**	0.057	0.237	1.014
A*02:01g	1.328	1.047	1.667	**0.025**	0.078	0.998	1.768
A*24:02g	1.208	0.993	1.463	0.058	0.157	0.957	1.525
A*02:07g	1.279	0.935	1.708	0.119	0.283	0.898	1.822
A*32:01g	0.656	0.326	1.162	0.188	0.390	0.317	1.354
A*02:03g	1.284	0.899	1.778	0.205	0.390	0.872	1.890
A*26:01g	1.165	0.808	1.624	0.409	0.605	0.790	1.717
A*11:01g	0.920	0.752	1.119	0.412	0.605	0.740	1.144
A*02:11g	0.782	0.423	1.311	0.429	0.605	0.425	1.441
A*03:01g	0.849	0.547	1.252	0.446	0.605	0.547	1.317
A*34:01	1.158	0.684	1.823	0.555	0.702	0.694	1.932
A*31:01g	1.099	0.732	1.582	0.630	0.748	0.738	1.637
A*30:01g	1.096	0.658	1.706	0.703	0.786	0.675	1.781
A*24:07	0.939	0.584	1.424	0.765	0.808	0.599	1.472
A*11:02g	0.944	0.474	1.664	0.856	0.856	0.507	1.758
**HLA-B Allele**	**OR**	**LCI**	**UCI**	**P value**	**adjusted P value**	**adj LCI**	**adj UCI**
B*40:02g	2.536	1.852	3.395	**0.000**	**0.000**	1.541	4.175
B*13:01g	1.881	1.389	2.492	**0.000**	**0.001**	1.199	2.949
B*55:02g	2.104	1.391	3.048	**0.000**	**0.003**	1.180	3.752
B*15:13	3.059	1.396	5.764	**0.002**	**0.021**	1.121	8.344
B*48:01g	1.878	1.208	2.777	**0.003**	**0.023**	1.054	3.347
B*44:03	0.554	0.360	0.811	**0.004**	**0.028**	0.319	0.961
B*46:01g	1.432	1.096	1.841	**0.007**	**0.038**	1.013	2.024
B*58:01g	0.623	0.428	0.874	**0.009**	**0.043**	0.390	0.994
B*35:03g	0.448	0.240	0.755	**0.010**	**0.043**	0.212	0.944
B*57:01g	0.511	0.285	0.836	**0.013**	0.054	0.259	1.007
B*07:05g	0.504	0.260	0.871	**0.026**	0.095	0.237	1.071
B*52:01g	0.621	0.392	0.930	**0.030**	0.099	0.364	1.058
B*15:01g	1.473	1.041	2.024	**0.035**	0.108	0.980	2.213
B*49:01	2.203	0.867	4.540	0.057	0.163	0.822	5.906
B*37:01g	0.600	0.286	1.092	0.130	0.325	0.271	1.325
B*38:02g	0.719	0.452	1.080	0.137	0.325	0.430	1.202
B*56:04	1.960	0.696	4.282	0.138	0.325	0.689	5.572
B*27:06	1.771	0.714	3.599	0.212	0.471	0.693	4.524
B*39:01g	1.375	0.781	2.229	0.251	0.528	0.753	2.511
B*44:02g	1.450	0.691	2.648	0.273	0.546	0.678	3.099
B*27:05g	1.450	0.710	2.604	0.294	0.552	0.697	3.018

B*35:02g	1.576	0.560	3.436	0.315	0.552	0.581	4.276
B*15:35	1.435	0.725	2.523	0.318	0.552	0.716	2.876
B*15:18g	0.703	0.286	1.416	0.387	0.640	0.294	1.684
B*13:02g	1.216	0.753	1.848	0.400	0.640	0.744	1.986
B*54:01g	0.812	0.463	1.310	0.428	0.653	0.462	1.426
B*50:01g	0.709	0.253	1.539	0.446	0.653	0.272	1.847
B*35:01g	1.152	0.823	1.568	0.457	0.653	0.815	1.629
B*51:01g	0.897	0.653	1.202	0.489	0.663	0.648	1.242
B*15:02g	0.888	0.619	1.232	0.497	0.663	0.617	1.278
B*51:02	1.279	0.545	2.505	0.521	0.672	0.579	2.823
B*51:06	1.267	0.451	2.759	0.600	0.750	0.501	3.209
B*15:11g	1.200	0.474	2.462	0.659	0.756	0.516	2.790
B*40:06	1.061	0.760	1.443	0.661	0.756	0.762	1.479
B*35:05	1.129	0.628	1.857	0.662	0.756	0.648	1.969
B*15:25g	1.103	0.557	1.938	0.717	0.796	0.585	2.078
B*55:01	0.909	0.377	1.808	0.811	0.856	0.414	1.995
B*40:01g	1.028	0.778	1.334	0.813	0.856	0.783	1.349
B*56:01g	1.085	0.447	2.170	0.839	0.861	0.495	2.376
B*07:02g	1.032	0.672	1.511	0.878	0.878	0.690	1.545

OR, Odds Ratio; LCI, Lower Confidence Interval; UCI, Upper Confidence Interval; adjusted P, P value adjusted for False Discovery Rate; adj LCI and UCI for OR were calculated with adjustment for False Discovery Rate. P values <0.05 are bolded.

Supplemental Table S6B: Association of HLA class I alleles in Black NMDP Population

HLA-A Allele	OR	LCI	UCI	P value	adjusted P value	adj LCI	adj UCI
A*69:01	7.871	2.682	18.562	**0.000**	**0.001**	1.732	35.758
A*02:01g	1.225	1.062	1.408	**0.006**	0.072	0.992	1.512
A*03:01g	0.776	0.642	0.931	**0.007**	0.072	0.596	1.011
A*36:01	1.335	1.044	1.682	**0.017**	0.126	0.962	1.854
A*02:60	2.641	0.953	5.789	**0.036**	0.207	0.805	8.663
A*66:02	1.447	0.962	2.084	0.067	0.322	0.874	2.396
A*23:01g	0.880	0.749	1.029	0.115	0.404	0.719	1.078
A*31:01g	1.342	0.888	1.939	0.139	0.404	0.824	2.188
A*80:01	0.641	0.319	1.134	0.164	0.404	0.296	1.389
A*68:02	0.868	0.708	1.053	0.171	0.404	0.682	1.104
A*02:02	0.837	0.650	1.060	0.173	0.404	0.624	1.121
A*68:01g	0.830	0.636	1.064	0.176	0.404	0.613	1.124
A*33:01	1.242	0.907	1.657	0.181	0.404	0.875	1.763
A*30:01g	1.109	0.926	1.318	0.283	0.585	0.905	1.358
A*32:01g	1.159	0.805	1.610	0.404	0.764	0.782	1.717
A*33:03g	0.927	0.746	1.138	0.488	0.764	0.731	1.174
A*26:01g	1.136	0.779	1.595	0.497	0.764	0.764	1.691
A*01:02	1.214	0.644	2.061	0.510	0.764	0.644	2.288

A*02:05g	1.132	0.790	1.566	0.514	0.764	0.780	1.642
A*01:01g	1.072	0.858	1.324	0.527	0.764	0.849	1.355
A*66:03	1.291	0.486	2.734	0.571	0.769	0.520	3.208
A*66:01g	0.893	0.594	1.285	0.583	0.769	0.594	1.343
A*74:01g	1.048	0.855	1.272	0.643	0.781	0.851	1.291
A*24:02g	0.937	0.692	1.239	0.663	0.781	0.693	1.268
A*34:02	1.054	0.819	1.334	0.673	0.781	0.820	1.355
A*30:04g	0.867	0.309	1.888	0.752	0.804	0.350	2.148
A*29:02g	0.962	0.736	1.234	0.769	0.804	0.740	1.250
A*30:02g	0.985	0.816	1.180	0.776	0.804	0.818	1.186
A*11:01g	0.984	0.648	1.426	0.936	0.936	0.664	1.458

**HLA-B Allele**	**OR**	**LCI**	**UCI**	**P value**	**adjusted P value**	**adj LCI**	**adj UCI**
B*14:02	2.400	1.937	2.943	**0.000**	**0.000**	1.699	3.391
B*15:03g	0.685	0.546	0.848	**0.001**	**0.017**	0.487	0.963
B*14:01	1.865	1.247	2.673	**0.003**	**0.040**	1.059	3.283
B*15:10	0.672	0.499	0.885	**0.007**	0.073	0.446	1.015
B*08:01g	1.312	1.048	1.622	**0.015**	0.122	0.966	1.782
B*39:10	1.526	1.007	2.209	**0.035**	0.240	0.892	2.610
B*07:05g	0.475	0.220	0.884	**0.045**	0.240	0.185	1.219
B*15:01g	1.485	0.987	2.138	**0.046**	0.240	0.892	2.471
B*44:03	0.801	0.632	1.000	0.063	0.279	0.594	1.078
B*49:01	1.253	0.977	1.583	0.066	0.279	0.920	1.707
B*58:02	0.823	0.634	1.050	0.139	0.359	0.598	1.133
B*39:06	1.964	0.694	4.331	0.140	0.359	0.641	6.021
B*82:01	1.686	0.779	3.152	0.145	0.359	0.718	3.962
B*35:01g	0.868	0.714	1.047	0.150	0.359	0.687	1.097
B*13:02g	0.650	0.335	1.122	0.157	0.359	0.316	1.338
B*57:03	0.819	0.614	1.067	0.160	0.359	0.589	1.138
B*57:01g	0.585	0.254	1.131	0.162	0.359	0.243	1.405
B*18:01g	1.184	0.923	1.494	0.170	0.359	0.892	1.570
B*51:01g	0.788	0.550	1.088	0.171	0.359	0.530	1.169
B*53:01g	1.105	0.955	1.274	0.176	0.359	0.935	1.305
B*41:02	0.601	0.261	1.164	0.179	0.359	0.259	1.398
B*81:01g	0.774	0.528	1.091	0.193	0.368	0.513	1.167
B*42:01	0.897	0.720	1.104	0.320	0.561	0.705	1.140
B*37:01g	0.692	0.296	1.352	0.336	0.561	0.300	1.597
B*40:01g	0.788	0.467	1.235	0.338	0.561	0.461	1.347
B*57:02	1.335	0.704	2.277	0.347	0.561	0.703	2.534
B*78:01	1.202	0.769	1.782	0.388	0.603	0.761	1.900
B*44:02g	1.147	0.810	1.572	0.483	0.716	0.800	1.644
B*14:03	1.334	0.553	2.667	0.494	0.716	0.575	3.093
B*15:16	0.901	0.625	1.252	0.560	0.784	0.622	1.305
B*55:01	1.197	0.570	2.186	0.596	0.808	0.590	2.427
B*42:02	0.878	0.492	1.435	0.646	0.848	0.499	1.544
B*15:17	0.870	0.415	1.585	0.686	0.873	0.433	1.748

B*50:01g	1.082	0.669	1.645	0.732	0.891	0.678	1.728
B*45:01g	1.036	0.836	1.269	0.743	0.891	0.834	1.287
B*07:02g	1.020	0.852	1.213	0.772	0.901	0.850	1.225
B*40:02g	1.085	0.510	2.001	0.810	0.915	0.542	2.175
B*58:01g	0.980	0.767	1.233	0.828	0.915	0.769	1.249
B*27:05g	1.026	0.608	1.611	0.887	0.920	0.627	1.679
B*52:01g	1.024	0.716	1.414	0.893	0.920	0.727	1.442
B*27:03	0.990	0.432	1.913	0.898	0.920	0.473	2.072
B*39:01g	1.034	0.368	2.256	0.941	0.941	0.426	2.513

OR, Odds Ratio; LCI, Lower Confidence Interval; UCI, Upper Confidence Interval; adjusted P, P value adjusted for False Discovery Rate; adj LCI and UCI for OR were calculated with adjustment for False Discovery Rate. P values <0.05 are bolded.

Supplemental Table S6B: Association of HLA class I alleles in Hispanic NMDP Population

HLA-A Allele	OR	LCI	UCI	P value	adjusted P value	adj LCI	adj UCI
A*02:06g	1.562	1.249	1.929	**0.000**	**0.004**	1.102	2.212
A*66:02	5.538	1.926	12.583	**0.000**	**0.004**	1.399	21.925
A*03:01g	0.703	0.564	0.866	**0.001**	**0.013**	0.517	0.955
A*33:01	1.561	1.158	2.057	**0.003**	**0.020**	1.050	2.322
A*29:02g	0.651	0.477	0.867	**0.005**	**0.029**	0.436	0.973
A*02:01g	1.163	1.012	1.336	**0.036**	0.160	0.969	1.397
A*33:03g	1.600	1.011	2.395	**0.037**	0.160	0.921	2.780
A*26:01g	0.700	0.483	0.978	**0.047**	0.177	0.450	1.091
A*11:01g	0.806	0.614	1.037	0.106	0.352	0.583	1.114
A*68:02	1.271	0.944	1.673	0.132	0.396	0.896	1.802
A*02:11g	1.627	0.792	2.933	0.163	0.407	0.744	3.556
A*02:05g	1.290	0.877	1.824	0.194	0.407	0.836	1.990
A*32:01g	1.205	0.894	1.588	0.202	0.407	0.862	1.686
A*30:02g	1.223	0.905	1.614	0.203	0.407	0.875	1.708
A*23:01g	0.821	0.597	1.098	0.204	0.407	0.580	1.162
A*66:01g	0.653	0.280	1.274	0.273	0.513	0.280	1.526
A*68:03	0.798	0.509	1.185	0.315	0.556	0.498	1.279
A*01:01g	0.909	0.737	1.111	0.364	0.606	0.725	1.141
A*25:01g	0.783	0.428	1.301	0.384	0.606	0.428	1.433
A*30:01g	1.136	0.794	1.571	0.495	0.735	0.784	1.645
A*24:02g	0.948	0.802	1.115	0.526	0.735	0.794	1.132
A*31:01g	1.063	0.835	1.334	0.612	0.735	0.828	1.364
A*02:02	0.891	0.455	1.553	0.632	0.735	0.464	1.709
A*29:01	1.236	0.440	2.698	0.640	0.735	0.487	3.136
A*68:01g	1.025	0.808	1.283	0.640	0.735	0.806	1.304
A*02:17	1.212	0.471	2.516	0.668	0.735	0.515	2.852
A*74:01g	0.873	0.435	1.547	0.672	0.735	0.459	1.660
A*30:04g	1.179	0.485	2.362	0.686	0.735	0.534	2.606
A*02:04	1.168	0.415	2.548	0.732	0.757	0.478	2.857
A*34:02	1.061	0.452	2.076	0.878	0.878	0.501	2.247

HLA-B Allele	OR	LCI	UCI	P value	adjusted P value	adj LCI	adj UCI
B*40:02g	1.919	1.590	2.299	**0.000**	**0.000**	1.409	2.613
B*14:02	1.632	1.323	1.993	**0.000**	**0.000**	1.183	2.252
B*44:03	0.613	0.467	0.789	**0.000**	**0.005**	0.413	0.910
B*35:03g	0.425	0.227	0.718	**0.004**	0.052	0.183	0.987
B*38:01	0.482	0.276	0.775	**0.005**	0.052	0.232	1.003
B*35:02g	0.569	0.311	0.944	**0.046**	0.310	0.263	1.229
B*07:02g	1.225	0.996	1.492	**0.049**	0.310	0.929	1.616
B*53:01g	1.394	0.982	1.916	0.051	0.310	0.889	2.186
B*27:05g	1.307	0.935	1.774	0.102	0.555	0.855	1.998
B*40:05	1.590	0.867	2.653	0.127	0.592	0.767	3.299
B*14:01	1.462	0.899	2.234	0.133	0.592	0.812	2.631
B*18:01g	0.823	0.618	1.073	0.166	0.644	0.579	1.170
B*15:17	0.564	0.206	1.208	0.199	0.644	0.188	1.690
B*39:08	1.584	0.782	2.832	0.201	0.644	0.711	3.533
B*15:15	0.661	0.324	1.182	0.209	0.644	0.299	1.460
B*58:01g	0.720	0.417	1.149	0.219	0.644	0.388	1.338
B*08:01g	1.152	0.906	1.444	0.234	0.644	0.868	1.528
B*15:03g	0.739	0.424	1.186	0.248	0.644	0.400	1.367
B*57:01g	0.757	0.452	1.181	0.256	0.644	0.428	1.339
B*42:01	0.647	0.265	1.296	0.280	0.644	0.256	1.634
B*15:01g	0.832	0.591	1.136	0.285	0.644	0.567	1.221
B*51:02	0.630	0.234	1.337	0.297	0.644	0.232	1.711
B*07:05g	0.638	0.241	1.341	0.304	0.644	0.240	1.696
B*41:01	0.751	0.400	1.272	0.334	0.644	0.389	1.452
B*56:01g	1.364	0.677	2.424	0.335	0.644	0.665	2.798
B*35:01g	0.896	0.716	1.109	0.343	0.644	0.700	1.148
B*39:06	1.169	0.828	1.600	0.363	0.644	0.808	1.692
B*39:01g	0.753	0.378	1.326	0.380	0.644	0.376	1.509
B*35:43g	0.737	0.363	1.318	0.384	0.644	0.361	1.508
B*51:01g	0.904	0.716	1.127	0.394	0.644	0.705	1.161
B*35:12	0.836	0.552	1.210	0.418	0.660	0.544	1.287
B*13:02g	1.172	0.764	1.712	0.439	0.668	0.756	1.816
B*45:01g	0.856	0.557	1.251	0.450	0.668	0.553	1.324
B*40:01g	0.857	0.539	1.286	0.490	0.707	0.538	1.366
B*15:30	0.771	0.283	1.646	0.560	0.771	0.305	1.948
B*15:10	1.219	0.576	2.239	0.566	0.771	0.596	2.494
B*44:02g	0.927	0.695	1.210	0.586	0.776	0.691	1.244
B*39:02	0.811	0.343	1.598	0.602	0.776	0.363	1.814
B*41:02	0.843	0.383	1.585	0.641	0.800	0.403	1.762
B*52:01g	0.927	0.657	1.267	0.653	0.800	0.659	1.305
B*37:01g	0.887	0.458	1.535	0.696	0.825	0.476	1.653
B*35:08	1.105	0.574	1.904	0.743	0.825	0.596	2.051
B*39:05	0.952	0.691	1.277	0.754	0.825	0.695	1.305
B*57:03	1.025	0.571	1.682	0.771	0.825	0.591	1.779
B*35:17	0.935	0.590	1.400	0.773	0.825	0.603	1.451

B*49:01	1.047	0.754	1.412	0.775	0.825	0.762	1.438
B*40:08	0.906	0.358	1.856	0.811	0.845	0.400	2.049
B*50:01g	1.024	0.664	1.501	0.857	0.874	0.681	1.540
B*48:01g	1.003	0.686	1.411	0.933	0.933	0.700	1.436

OR, Odds Ratio; LCI, Lower Confidence Interval; UCI, Upper Confidence Interval; adjusted P, P value adjusted for False Discovery Rate; adj LCI and UCI for OR were calculated with adjustment for False Discovery Rate. P values <0.05 are bolded.

Supplemental Table S6D: Association of HLA class I alleles in Native American NMDP Population

HLA-A Allele	OR	LCI	UCI	P value	adjusted P value	adj LCI	adj UCI
A*02:06g	4.340	1.576	9.844	**0.004**	**0.018**	1.315	14.326
A*31:01g	2.730	1.222	5.506	**0.008**	**0.020**	1.130	6.596
A*02:01g	2.039	1.093	3.825	**0.029**	**0.049**	1.025	4.056
A*03:01g	1.359	0.609	2.740	0.418	0.522	0.624	2.960
A*24:02g	1.222	0.563	2.423	0.592	0.592	0.594	2.513
HLA-B Allele	OR	LCI	UCI	P value	adjusted P value	adj LCI	adj UCI
B*39:01g	3.561	1.220	8.310	**0.008**	**0.040**	1.037	12.228
B*35:01g	2.100	0.948	4.216	0.052	0.130	0.875	5.041
B*27:05g	2.110	0.723	4.920	0.119	0.198	0.747	5.961
B*44:02g	1.728	0.653	3.828	0.217	0.254	0.695	4.295
B*15:01g	1.726	0.592	4.024	0.254	0.254	0.675	4.409

OR, Odds Ratio; LCI, Lower Confidence Interval; UCI, Upper Confidence Interval; adjusted P, P value adjusted for False Discovery Rate; adj LCI and UCI for OR were calculated with adjustment for False Discovery Rate. P values <0.05 are bolded.

Supplemental Table S6E: Association of HLA class I alleles in White NMDP Population

**HLA-A Allele**	**OR**	**LCI**	**UCI**	**P value**	**adjusted P value**	**adj LCI**	**adj UCI**
A*02:01g	1.216	1.140	1.297	**0.000**	**0.000**	1.096	1.350
A*33:01	1.790	1.471	2.159	**0.000**	**0.000**	1.342	2.388
A*68:02	1.545	1.274	1.858	**0.000**	**0.000**	1.179	2.027
A*29:02g	0.722	0.619	0.837	**0.000**	**0.000**	0.586	0.891
A*23:01g	0.685	0.566	0.819	**0.000**	**0.000**	0.533	0.878
A*30:02g	0.580	0.430	0.765	**0.000**	**0.001**	0.397	0.848
A*24:02g	0.850	0.776	0.930	**0.000**	**0.002**	0.756	0.956
A*26:01g	0.797	0.686	0.922	**0.003**	**0.012**	0.661	0.962
A*29:01	0.571	0.323	0.929	0.054	0.186	0.295	1.104
A*11:01g	0.911	0.821	1.009	0.076	0.236	0.803	1.034
A*74:01g	0.504	0.199	1.042	0.110	0.310	0.186	1.364
A*32:01g	1.098	0.969	1.239	0.137	0.353	0.948	1.271
A*02:17	1.764	0.812	3.381	0.150	0.357	0.765	4.067
A*68:03	1.783	0.738	3.682	0.181	0.401	0.705	4.510
A*66:01g	0.763	0.502	1.108	0.200	0.413	0.484	1.201
A*01:01g	0.956	0.889	1.028	0.229	0.443	0.881	1.039
A*02:05g	0.866	0.676	1.091	0.266	0.447	0.661	1.133
A*30:04g	0.691	0.371	1.171	0.273	0.447	0.361	1.323
A*34:02	1.316	0.779	2.084	0.274	0.447	0.767	2.257
A*24:03g	0.760	0.441	1.217	0.352	0.546	0.435	1.328
A*02:02	0.776	0.429	1.285	0.381	0.562	0.430	1.401
A*02:06g	0.836	0.541	1.231	0.405	0.563	0.539	1.297
A*25:01g	0.931	0.780	1.102	0.417	0.563	0.775	1.119
A*68:01g	1.048	0.917	1.193	0.483	0.623	0.913	1.204
A*26:08	1.215	0.575	2.261	0.587	0.691	0.597	2.472
A*03:02g	0.901	0.585	1.323	0.590	0.691	0.589	1.377
A*31:01g	1.039	0.899	1.194	0.602	0.691	0.897	1.202
A*30:01g	1.042	0.864	1.245	0.664	0.735	0.865	1.256

A*33:03g	1.023	0.762	1.342	0.739	0.790	0.768	1.362
A*69:01	0.960	0.548	1.557	0.808	0.835	0.568	1.620
A*03:01g	0.992	0.920	1.070	0.843	0.843	0.920	1.070
**HLA-B Allele**	**OR**	**LCI**	**UCI**	**P value**	**adjusted P value**	**adj LCI**	**adj UCI**
B*14:02	2.045	1.835	2.275	**0.000**	**0.000**	1.710	2.447
B*40:02g	2.107	1.816	2.433	**0.000**	**0.000**	1.676	2.650
B*44:03	0.613	0.534	0.700	**0.000**	**0.000**	0.501	0.751
B*57:01g	0.622	0.531	0.725	**0.000**	**0.000**	0.496	0.781
B*35:01g	0.703	0.623	0.790	**0.000**	**0.000**	0.594	0.833
B*07:02g	1.223	1.135	1.316	**0.000**	**0.000**	1.103	1.355
B*38:01	0.602	0.490	0.732	**0.000**	**0.000**	0.458	0.792
B*15:01g	0.826	0.737	0.924	**0.001**	**0.006**	0.710	0.962
B*08:01g	1.140	1.053	1.233	**0.001**	**0.006**	1.027	1.265
B*35:02g	0.708	0.543	0.906	**0.008**	**0.039**	0.507	0.988
B*53:01g	0.639	0.448	0.881	**0.009**	**0.040**	0.414	0.986
B*15:03g	0.496	0.277	0.813	**0.011**	**0.044**	0.251	0.979
B*41:01	0.643	0.441	0.901	**0.015**	0.055	0.411	1.004
B*39:06	1.379	1.059	1.765	**0.022**	0.066	1.004	1.893
B*18:01g	0.868	0.768	0.978	**0.022**	0.066	0.748	1.008
B*40:01g	0.874	0.777	0.980	**0.023**	0.066	0.758	1.006
B*57:03	0.422	0.170	0.861	**0.040**	0.109	0.159	1.118
B*15:17	0.635	0.399	0.957	**0.043**	0.109	0.378	1.070
B*13:02g	1.162	1.001	1.341	**0.044**	0.109	0.977	1.382
B*39:05	1.799	0.927	3.180	0.061	0.139	0.878	3.688
B*50:01g	0.791	0.612	1.003	0.062	0.139	0.593	1.054
B*58:01g	0.781	0.593	1.009	0.069	0.147	0.575	1.062
B*51:01g	1.096	0.982	1.219	0.098	0.200	0.968	1.240
B*15:10	0.517	0.206	1.059	0.109	0.213	0.207	1.292
B*14:01	1.194	0.956	1.473	0.123	0.231	0.935	1.525
B*07:05g	0.682	0.416	1.048	0.140	0.253	0.405	1.147
B*52:01g	0.844	0.668	1.052	0.145	0.253	0.655	1.088
B*27:05g	1.089	0.958	1.232	0.192	0.323	0.947	1.251
B*42:01	0.655	0.310	1.206	0.216	0.351	0.313	1.371
B*48:01g	0.696	0.330	1.284	0.291	0.450	0.334	1.453
B*44:02g	1.047	0.960	1.141	0.296	0.450	0.953	1.151
B*45:01g	1.146	0.877	1.472	0.308	0.452	0.867	1.516
B*47:01	0.781	0.452	1.251	0.337	0.481	0.453	1.345
B*27:02	0.843	0.560	1.216	0.424	0.586	0.557	1.276
B*41:02	0.903	0.628	1.254	0.563	0.723	0.626	1.302
B*44:05	0.869	0.529	1.343	0.570	0.723	0.532	1.420
B*35:08	1.037	0.748	1.399	0.570	0.723	0.746	1.443
B*51:08	1.189	0.607	2.096	0.585	0.723	0.626	2.258
B*40:06	0.860	0.400	1.611	0.672	0.810	0.420	1.763
B*39:01g	0.994	0.787	1.238	0.720	0.827	0.786	1.256
B*15:16	1.106	0.495	2.137	0.722	0.827	0.523	2.339

B*35:03g	0.973	0.806	1.163	0.763	0.854	0.806	1.173
B*15:18g	0.939	0.569	1.457	0.795	0.869	0.583	1.513
B*56:01g	0.968	0.701	1.301	0.834	0.870	0.708	1.323
B*55:01	1.017	0.849	1.208	0.851	0.870	0.851	1.215
B*37:01g	0.981	0.799	1.191	0.851	0.870	0.803	1.199
B*49:01	1.007	0.846	1.190	0.937	0.937	0.849	1.194

OR, Odds Ratio; LCI, Lower Confidence Interval; UCI, Upper Confidence Interval; adjusted P, P value adjusted for False Discovery Rate; adj LCI and UCI for OR were calculated with adjustment for False Discovery Rate. P values <0.05 are bolded.

Supplemental Table 7. Association Analysis of AA with HLA allele groups based on peptide-binding pocket structure

Supplemental Table 7A. Groups of AA Risk-Like HLA Class I Alleles Based on Peptide-Binding Pocket Identity Used in Group-Based Association Analysis

Risk-PPSM1 Group (B*14:01 & B*14:02-like)	Risk-PPSM2 Group (B*40:02 & B*41:02-like)	Risk-PPSM3 Group (B*56:01, B*55:02-like)	Risk-PPSM4 Group (A*02:01 & A*02:06-like)	A*33:03-like Alleles	A*68:01-like Alleles	A*74:01-like Alleles	B*07:02-like Alleles	B*08:01-like Alleles	B*13:02-like Alleles	B*18:01-like Alleles	B*27:05-like Alleles	B*38:02-like Alleles	B*49:01-like Alleles	B*50:02-like Alleles	B*53:01-like Alleles
**B*14:01**	**B*40:02**	B*55:01	**A*02:01**	A*33:01	**A*68:01**	**A*74:01**	**B*07:02**	**B*08:01**	**B*13:02**	**B*18:01**	**B*27:05**	**B*38:02**	B*44:18	B*45:01	**B*53:01**
**B*14:02**	B*40:05	**B*55:02**	A*02:03	**A*33:03**	A*68:08	A*74:02	B*07:04	B*08:11	B*1303	B*18:05	B*27:10	B*38:08	**B*49:01**	B*45:03	B*53:02
B*14:03	B*40:15	B*55:03	A*02:09	A*33:04	A*68:09	A*74:03	B*07:15	B*08:13	B*1308	B*18:06	B*27:13			B*45:04	B*53:06
B*14:07	B*40:16	B*55:05	A*02:12	A*33:05	A*68:12	A*74:05	B*07:19	B*08:15		B*18:10	B*27:17			B*45:05	B*53:08
	B*40:29	B*55:09	A*02:13	A*33:06	A*68:16	A*74:07	B*07:20	B*08:18		B*18:11	B*27:28			B*45:07	B*53:10
	B*40:35	B*55:15	A*02:16	A*33:07	A*68:19	A*74:08	B*07:21	B*08:19		B*18:13				B*50:01	
	B*40:39	B*55:19	A*02:19		A*68:21	A*74:09	B*07:22	B*08:20		B*18:15				**B*50:02**	
	B*40:56	**B*56:01**	A*02:22		A*68:22	A*74:11	B*07:24	B*08:21		B*18:19				B*50:04	
	B*40:57	B*56:13	A*02:24		A*68:24		B*07:25	B*08:22		B*18:20					
	**B*41:02**		A*02:25		A*68:25		B*07:26	B*08:24							
			A*02:26		A*68:26		B*07:30	B*08:25							
			A*02:27				B*07:31								
			A*02:30				B*07:33								
			A*02:31				B*07:35								
			A*02:36				B*07:39								
			A*02:37				B*07:41								
			A*02:38				B*07:42								
			A*02:40				B*07:43								
			A*02:43												
			A*02:45												
			A*02:46												
			A*02:49												
			A*02:59												
			A*02:66												
			A*02:67												
			A*02:68												
			A*02:70												
			A*02:71												
			A*02:74												
			A*02:75												
			A*02:77												
			A*02:82												
			A*02:83												
			A*02:85												
			A*02:86												
			**A*02:06**												
			A*02:21												
			A*02:28												
			A*02:44												
			A*02:51												
			A*02:54												
			A*02:61												
			A*02:72												
			A*02:79												

Supplemental Table 7B. Groups of Non-Risk-Like HLA Class I Alleles Based on Peptide-Binding Pocket Identity Used in Group-Based Association Analysis

NR-PPSM1 Group (A*03:01 & A*11:01-like)	NR-PPSM2 Group (A*23:01 & A*24:02-like)	NR-PPSM3 (B*44:02 & B*44:03-like)	A*01:01-like Alleles
**A*03:01**	**A*23:01**	**B*44:02**	**A*01:01**
A*03:02	A*23:02	**B*44:03**	A*01:04
A*03:04	A*23:03	B*44:04	A*01:06
A*03:05	A*23:04	B*44:07	A*01:08
A*03:06	A*23:06	B*44:13	A*01:09
A*03:07	A*23:07	B*44:16	A*01:10
A*03:10	A*23:08	B*44:21	A*01:11
A*03:13	A*23:10	B*44:22	A*01:12
A*03:14	**A*24:02**	B*44:24	A*01:14
A*03:16	A*24:03	B*44:26	A*01:15
A*03:17	A*24:05	B*44:27	A*36:01
A*03:12	A*24:06	B*44:28	A*36:02
**A*11:01**	A*24:08	B*44:29	A*36:04
A*11:02	A*24:09	B*44:30	
A*11:03	A*24:10	B*44:32	
A*11:04	A*24:11	B*44:33	
A*11:05	A*24:13	B*44:35	
A*11:07	A*24:18	B*44:36	
A*11:08	A*24:20	B*44:37	
A*11:09	A*24:21	B*44:38	
A*11:12	A*24:22		
A*11:13	A*24:23		
A*11:14	A*24:26		
A*11:15	A*24:27		
A*11:16	A*24:29		
A*11:20	A*24:33		
A*11:21	A*24:34		
A*11:23	A*24:35		
	A*24:37		
	A*24:38		
	A*24:39		
	A*24:40		
	A*24:43		
	A*24:46		
	A*24:47		
	A*24:48		
	A*24:49		

Supplemental Table 7C. List of Other Alleles Analyzed in the Other HLA-A and Other HLA-B Allele Groups

A*01:02	A*02:186	A*03:33	A*24:07	A*26:01g	A*31:20	A*66:12	B*07:46	B*15:13	B*18:07	B*35:116	B*39:12	B*40:82	B*51:03	B*55:44
A*01:03	A*02:189	A*03:35	A*24:100	A*26:02	A*31:25	A*68:02	B*07:62	B*15:132	B*18:14	B*35:119	B*39:13	B*40:84	B*51:04	B*55:47
A*01:105	A*02:197	A*03:44	A*24:102	A*26:03	A*31:26	A*68:03	B*07:68	B*15:134	B*18:18	B*35:12	B*39:14	B*40:87	B*51:05	B*55:56
A*01:110	A*02:20	A*03:46	A*24:104	A*26:07	A*31:28	A*68:05	B*07:73	B*15:135	B*18:26	B*35:13	B*39:15	B*40:89	B*51:06	B*56:02
A*01:112	A*02:200	A*03:50	A*24:105	A*26:08	A*31:29	A*68:06	B*07:80	B*15:14	B*18:28	B*35:137g	B*39:16	B*40:94	B*51:07	B*56:03
A*01:171	A*02:201	A*03:51	A*24:107	A*26:09	A*31:30	A*68:07	B*07:81	B*15:15	B*18:31	B*35:14	B*39:20	B*41:01	B*51:08	B*56:04
A*01:25	A*02:203	A*03:60	A*24:112	A*26:10	A*31:35	A*68:111	B*07:97	B*15:154	B*18:33	B*35:158	B*39:22	B*41:03	B*51:09	B*56:09
A*01:26	A*02:207	A*03:63	A*24:120	A*26:13	A*31:39	A*68:125	B*08:04	B*15:16	B*18:34	B*35:16	B*39:24	B*41:16	B*51:10	B*56:15
A*01:29	A*02:211	A*03:65	A*24:121	A*26:14	A*31:41	A*68:13	B*08:09	B*15:162	B*18:39	B*35:169	B*39:29	B*41:21	B*51:100	B*56:29
A*01:30	A*02:219	A*11:103	A*24:135	A*26:15	A*31:43	A*68:15	B*08:104	B*15:169	B*18:40	B*35:17	B*39:31	B*42:01	B*51:105	B*56:30
A*01:38	A*02:221	A*11:104	A*24:136	A*26:16	A*31:66	A*68:17	B*08:12	B*15:17	B*18:41	B*35:172	B*39:35	B*42:02	B*51:109	B*57:01g
A*01:41	A*02:230	A*11:112	A*24:14	A*26:17	A*31:68	A*68:31	B*08:14	B*15:175	B*18:46	B*35:189	B*39:44	B*42:05	B*51:119	B*57:02
A*01:42	A*02:258	A*11:114	A*24:15	A*26:23	A*31:70	A*68:32	B*08:33	B*15:183	B*18:47	B*35:19	B*39:45	B*42:16	B*51:12	B*57:03
A*01:43	A*02:259	A*11:116	A*24:156	A*26:34	A*31:84	A*68:34	B*08:36	B*15:184	B*18:59	B*35:199	B*39:47	B*44:05	B*51:18	B*57:04
A*01:50	A*02:271	A*11:117	A*24:167	A*26:41	A*32:01g	A*68:36	B*08:58	B*15:18g	B*18:68	B*35:20	B*39:60	B*44:06	B*51:19	B*57:06
A*01:58	A*02:283	A*11:119	A*24:17	A*26:70	A*32:03	A*68:37	B*08:59	B*15:20	B*27:01	B*35:21	B*39:62	B*44:08	B*51:21	B*57:15
A*01:59	A*02:29	A*11:19	A*24:175	A*29:01	A*32:04	A*68:55	B*08:63	B*15:21	B*27:02	B*35:24	B*39:67	B*44:09	B*51:22	B*57:17
A*01:67	A*02:302	A*11:22	A*24:178	A*29:02g	A*32:06	A*68:67	B*08:71	B*15:215	B*27:04g	B*35:26	B*40:01g	B*44:10	B*51:24	B*57:19
A*01:88	A*02:307	A*11:24	A*24:179	A*29:06	A*32:07	A*68:71	B*13:01g	B*15:219	B*27:06	B*35:27	B*40:03g	B*44:109	B*51:29	B*57:24
A*01:89	A*02:316	A*11:27	A*24:182	A*29:09	A*32:08	A*68:74	B*13:10	B*15:23	B*27:07	B*35:28	B*40:04	B*44:121	B*51:43	B*58:01g
A*01:92	A*02:317	A*11:29	A*24:186	A*29:10	A*32:11	A*68:75	B*13:14	B*15:237	B*27:08	B*35:29	B*40:06	B*44:125	B*51:46	B*58:02
A*01:93	A*02:330	A*11:30	A*24:192	A*29:32	A*32:29	A*68:79	B*13:18	B*15:24	B*27:09	B*35:30	B*40:08	B*44:17	B*51:49	B*58:16
A*01:94	A*02:333	A*11:32	A*24:193	A*29:34	A*32:31	A*68:84	B*13:22	B*15:252	B*27:12	B*35:31	B*40:09	B*44:39	B*51:69	B*58:19
A*02:02	A*02:334	A*11:33	A*24:194	A*29:37	A*32:39	A*68:88	B*13:25	B*15:256	B*27:14	B*35:36	B*40:10	B*44:50	B*51:71	B*58:22
A*02:04	A*02:335	A*11:36	A*24:195	A*29:44	A*33:08	A*68:91	B*13:29	B*15:25g	B*27:15	B*35:37	B*40:102	B*44:65	B*51:75	B*58:25
A*02:05g	A*02:340	A*11:40	A*24:25	A*30:01g	A*33:18	A*69:01	B*13:30	B*15:27	B*27:19	B*35:41	B*40:104	B*44:68	B*51:78	B*59:01g
A*02:07g	A*02:341	A*11:41	A*24:28	A*30:02g	A*33:20	A*74:04	B*13:43	B*15:28	B*27:21	B*35:43g	B*40:107	B*44:71	B*51:80	B*67:01g
A*02:08	A*02:346	A*11:42	A*24:292	A*30:03	A*33:22	A*74:13	B*13:50	B*15:29	B*27:23	B*35:48	B*40:11	B*44:72	B*51:84	B*73:01
A*02:106	A*02:347	A*11:43	A*24:30	A*30:04g	A*33:23	A*80:01	B*13:54	B*15:30	B*27:45	B*35:55	B*40:111	B*44:73	B*51:95	B*78:01
A*02:10g	A*02:35	A*11:51	A*24:31	A*30:06	A*33:27	B*07:03	B*13:60	B*15:31	B*27:70	B*35:64	B*40:116	B*44:76	B*51:96	B*78:02
A*02:11g	A*02:403	A*11:55	A*24:32	A*30:08	A*33:29	B*07:05g	B*14:05	B*15:33	B*27:71	B*35:65Q	B*40:12	B*44:84	B*52:01g	B*81:01g
A*02:122	A*02:483	A*11:56	A*24:50	A*30:10	A*33:45	B*07:07	B*14:18	B*15:34	B*27:72	B*35:72	B*40:122	B*44:86	B*52:04	B*82:01
A*02:123	A*02:58	A*11:62	A*24:53	A*30:18	A*33:51	B*07:09	B*14:20	B*15:35	B*27:79	B*35:77	B*40:134	B*44:91	B*52:08	B*82:02
A*02:127	A*02:60	A*11:88	A*24:56	A*30:19	A*33:53	B*07:10	B*14:26	B*15:37	B*35:01g	B*35:89	B*40:138	B*46:01g	B*52:11	
A*02:136	A*02:64	A*11:97	A*24:57	A*30:20	A*33:56	B*07:101	B*15:01g	B*15:39	B*35:02g	B*37:01g	B*40:145	B*46:10	B*52:17	

A*02:137	A*02:95	A*23:13	A*24:67	A*30:25	A*33:60	B*07:103	B*15:02g	B*15:40	B*35:03g	B*37:02	B*40:152	B*46:12	B*52:22
A*02:139	A*02:96	A*23:15	A*24:69	A*30:29	A*33:66	B*07:104	B*15:03g	B*15:43	B*35:04	B*38:01	B*40:18	B*47:01	B*52:23
A*02:144	A*03:08	A*23:21	A*24:78	A*30:32	A*34:01	B*07:105	B*15:04	B*15:45	B*35:05	B*38:06	B*40:19	B*47:02	B*53:04
A*02:148	A*03:103	A*23:22	A*24:81	A*30:35	A*34:02	B*07:106	B*15:05	B*15:48	B*35:06	B*38:09	B*40:198	B*47:03	B*53:05
A*02:154	A*03:119	A*23:24	A*24:87	A*30:38	A*34:03	B*07:108	B*15:06	B*15:56	B*35:07	B*38:12	B*40:21	B*48:01g	B*53:23
A*02:157	A*03:120	A*23:26	A*24:93	A*30:39	A*34:04	B*07:112	B*15:07	B*15:63	B*35:08	B*39:01g	B*40:217	B*48:02	B*53:26
A*02:158	A*03:121	A*23:33	A*24:98	A*30:54	A*34:05	B*07:12	B*15:08	B*15:65	B*35:09	B*39:02	B*40:27	B*48:03	B*54:01g
A*02:162	A*03:124	A*23:34	A*25:01g	A*31:01g	A*34:06	B*07:121	B*15:09	B*15:71	B*35:10	B*39:03	B*40:32	B*48:04	B*54:03
A*02:17	A*03:133	A*23:37	A*25:02	A*31:02	A*34:08	B*07:127	B*15:10	B*15:73	B*35:101	B*39:04	B*40:33	B*48:07	B*55:04
A*02:171	A*03:137	A*23:39	A*25:03	A*31:06	A*34:09	B*07:14	B*15:101	B*15:78	B*35:103	B*39:05	B*40:40g	B*48:11	B*55:20
A*02:177	A*03:138	A*23:43	A*25:04	A*31:08	A*36:03	B*07:23	B*15:118	B*15:82	B*35:104	B*39:06	B*40:48	B*48:21	B*55:25
A*02:181	A*03:143	A*23:48	A*25:05	A*31:09	A*66:01g	B*07:29	B*15:11g	B*15:90	B*35:107	B*39:08	B*40:49	B*49:13	B*55:39
A*02:182	A*03:15	A*23:53	A*25:09	A*31:11	A*66:02	B*07:37	B*15:128	B*18:02	B*35:108	B*39:09	B*40:50	B*49:16	B*55:40
A*02:186	A*03:163	A*23:54	A*25:14	A*31:13	A*66:03	B*07:38	B*15:129	B*18:03	B*35:11	B*39:10	B*40:64	B*51:01g	B*55:42
A*02:189	A*03:31	A*23:56	A*25:15	A*31:19	A*66:04	B*07:45	B*15:12g	B*18:04	B*35:111	B*39:11	B*40:81	B*51:02	B*55:43

Supplemental Table 7D. Association Analysis of AA with HLA allele groups in Asian and Pacific Islander and Black NMDP Populations

	Asian and Pacific Islander							Black						
	Cases	Case Al-lele Freq	Cntrl	Control Allele Freq	OR (95% CI)	P	Padj	Cases	Case Allele Freq	Cntrl	Control Allele Freq	OR (95% CI)	P	Padj
**Risk Allele -Like Group**														
Risk Pocket 1 Group (B*14:01 & B*14:02-like)	4	0.004	332	0.003	n/a			138	0.067	3203	0.032	2.254 (1.868-2.7)	**0.000**	**0.000**
Risk Pocket 2 Group (B*40:02 & B*41:02-like)	48	0.052	2272	0.023	2.486 (1.815-3.326)	**0.000**	**0.000**	18	0.009	1087	0.011	0.814 (0.492-1.259)	0.402	0.581
Risk Pocket 3 Group (B*56:01, B*54:01, B*55:02-like)	41	0.044	2911	0.029	1.551 (1.101-2.122)	**0.010**	**0.050**	12	0.006	625	0.006	0.966 (0.52-1.625)	0.877	0.950
Risk Pocket 4 Group (A*02:01 & A*02:06-like)	193	0.209	15289	0.153	1.554 (1.284-1.875)	**0.000**	**0.000**	282	0.137	11976	0.120	1.221 (1.059-1.403)	**0.007**	**0.029**
A*33:03-like Alleles	66	0.071	10253	0.103	0.669 (0.508-0.864)	**0.003**	**0.008**	148	0.072	7028	0.070	1.022 (0.852-1.218)	0.808	0.808
A*68:01-like Alleles	13	0.014	2682	0.027	0.487 (0.259-0.826)	**0.014**	**0.025**	65	0.032	3713	0.037	0.828 (0.634-1.061)	0.171	0.273
A*74:01-like Alleles	4	0.004	159	0.002	n/a			117	0.057	5482	0.055	1.041 (0.85-1.262)	0.693	0.792
B*07:02-like Alleles	28	0.030	2714	0.027	1.031 (0.671-1.51)	0.882	0.882	152	0.074	7310	0.073	1.027 (0.858-1.221)	0.739	0.873
B*08:01 like Alleles	6	0.006	1739	0.017	n/a			94	0.046	3513	0.035	1.312 (1.048-1.621)	**0.015**	0.064
B*13:02-like Alleles	22	0.024	1857	0.019	1.215 (0.753-1.848)	0.400	0.505	11	0.005	826	0.008	0.646 (0.334-1.117)	0.153	0.300
B*18:01-like Alleles	3	0.003	1395	0.014	n/a			74	0.036	3079	0.031	1.182 (0.921-1.493)	0.173	0.300
B*27:05-like Alleles	10	0.010	729	0.007	1.441 (0.706-2.587)	0.302	0.503	24	0.012	1178	0.012	1 (0.646-1.47)	0.999	0.999
B*38:02-like Alleles	22	0.023	3318	0.033	0.719 (0.452-1.08)	0.137	0.274	0	0.000	11	0.000	n/a		
B*49:01-like Alleles	6	0.006	299	0.003	2.203 (0.867-4.54)	0.057	0.143	76	0.037	2923	0.029	1.253 (0.976-1.582)	0.067	0.217
B*50:02-like Alleles	7	0.008	926	0.009	0.709 (0.28-1.451)	0.404	0.505	128	0.062	5745	0.057	1.062 (0.872-1.281)	0.545	0.708
B*53:01-like Alleles	2	0.002	89	0.001	n/a			273	0.133	11844	0.118	1.105 (0.955-1.274)	0.176	0.300
**Non Risk Allele -Like Group**														
NR Pocket 1 Group (A*03:01 & A*11:01-like)	199	0.215	23047	0.230	0.85 (0.701-1.026)	0.094	0.111	163	0.079	9570	0.096	0.785 (0.658-0.93)	**0.006**	**0.029**
NR Pocket 2 Group (A*23:01 & A*24:02-like)	181	0.196	17525	0.175	1.179 (0.972-1.425)	0.095	0.111	247	0.120	13609	0.136	0.889 (0.767-1.028)	0.116	0.233
NR Pocket 3 (B*44:02 & B*44:03-like)	35	0.038	5548	0.055	0.667 (0.462-0.932)	**0.024**	0.079	124	0.060	6591	0.066	0.876 (0.718-1.06)	0.185	0.300
A*01:01-like Alleles	41	0.044	6673	0.067	0.599 (0.419-0.83)	**0.003**	**0.008**	176	0.086	7116	0.071	1.177 (0.99-1.39)	0.060	0.161
**Other Alleles**														

**Other A Alleles**	228	0.246	24371	0.244	1.015 (0.843-1.22)	0.869	0.869	860	0.418	41505	0.415	1.045 (0.918-1.192)	0.510	0.680
**Other B Alleles**	693	0.748	75868	0.759	0.949 (0.673-1.387)	0.737	0.818	934	0.454	52064	0.521	0.726 (0.635-0.833)	**0.000**	**0.000**

Risk allele groups: Groups of HLA-A and HLA-B alleles with the same peptide binding pocket residues as 19 AA risk alleles identified in this study. Non-Risk allele groups: groups of alleles with same peptide binding pocket structures as the identified HLA-A and HLA-B Non-Risk alleles. In cases where insufficient number of patients had alleles from a particular allele group for statistically robust comparison, OR and p values could not be calculated (marked as not available, "n/a"). Cntrl, control. OR, Odds Ratio. CI, confidence interval; P, P-value; Padj, P value adjusted for multiple comparisons. Statistically significant P-value (<0.05) are **bolded**. For statistically significant comparisons, OR >1 are colored in red, and <1 are colored in blue. OR where the comparison showed significant differences with an OR discrepant to risk allele status are in purple.

Supplemental Table 7E. Association Analysis of AA with HLA allele groups in Hispanic and Native American NMDP Populations

	**Hispanic**							**Native American**						
	**Cases**	**Case Allele Freq**	**Cntrl**	**Control Allele Freq**	**OR (95% CI)**	**P**	**Padj**	**Cases**	**Case Allele Freq**	**Cntrl**	**Control Allele Freq**	**OR (95% CI)**	**P**	**Padj**
**Risk Allele -Like Group**														
Risk Pocket 1 Group (B*14:01 & B*14:02-like)	140	0.083	5078	0.051	1.667 (1.374-2.006)	**0.000**	**0.000**	3	0.038	2042	0.033	n/a		
Risk Pocket 2 Group (B*40:02 & B*41:02-like)	170	0.101	5907	0.059	1.809 (1.514-2.148)	**0.000**	**0.000**	6	0.075	2414	0.039	n/a		
Risk Pocket 3 Group (B*56:01, B*54:01, B*55:02-like)	20	0.012	1302	0.013	0.921 (0.57-1.399)	0.717	0.775	2	0.023	1023	0.016	n/a		
Risk Pocket 4 Group (A*02:01 & A*02:06-like)	485	0.288	24814	0.248	1.266 (1.105-1.451)	**0.001**	**0.002**	32	0.398	13481	0.217	2.93 (1.557-5.739)	**0.001**	**0.005**
A*33:03-like Alleles	73	0.043	2859	0.029	1.576 (1.224-1.997)	**0.000**	**0.002**	2	0.025	1999	0.032	n/a		
A*68:01-like Alleles	84	0.050	4819	0.048	1.024 (0.807-1.282)	0.652	0.704	1	0.010	2610	0.042	n/a		
A*74:01-like Alleles	10	0.006	700	0.007	0.852 (0.424-1.51)	0.618	0.704	0	0.000	626	0.010	n/a		
B*07:02-like Alleles	115	0.068	5705	0.057	1.223 (0.995-1.49)	0.050	0.109	5	0.063	4674	0.075	n/a		
B*08:01 like Alleles	82	0.049	4288	0.043	1.152 (0.906-1.444)	0.234	0.338	2	0.025	3604	0.058	n/a		
B*13:02-like Alleles	25	0.015	1289	0.013	1.171 (0.763-1.71)	0.442	0.574	1	0.013	732	0.012	n/a		
B*18:01-like Alleles	58	0.035	4016	0.040	0.82 (0.616-1.069)	0.158	0.258	3	0.038	2140	0.034	n/a		
B*27:05-like Alleles	42	0.025	1941	0.019	1.33 (0.959-1.793)	0.075	0.139	5	0.063	2053	0.033	2.1 (0.72-4.896)	0.121	0.364
B*38:02-like Alleles	0	0.000	27	0.000	n/a			0	0.000	27	0.000	n/a		
B*49:01-like Alleles	43	0.026	2427	0.024	1.047 (0.754-1.412)	0.775	0.775	1	0.013	1153	0.019	n/a		
B*50:02-like Alleles	57	0.034	3379	0.034	0.944 (0.705-1.237)	0.690	0.775	0	0.000	1930	0.031	n/a		
B*53:01-like Alleles	41	0.024	1610	0.016	1.434 (1.015-1.963)	**0.032**	0.083	0	0.000	1202	0.019	n/a		
**Non Risk Allele -Like Group**														

NR Pocket 1 Group (A*03:01 & A*11:01-like)	165	0.098	12905	0.129	0.735 (0.616-0.873)	**0.001**	**0.002**	14	0.175	8727	0.140	1.231 (0.602-2.364)	0.548	0.730
NR Pocket 2 Group (A*23:01 & A*24:02-like)	246	0.146	15972	0.160	0.897 (0.768-1.044)	0.167	0.334	12	0.150	9871	0.159	0.926 (0.443-1.802)	0.829	0.829
NR Pocket 3 (B*44:02 & B*44:03-like)	119	0.071	9455	0.095	0.705 (0.575-0.857)	**0.001**	**0.002**	9	0.113	7470	0.120	1.01 (0.452-2.035)	0.979	0.979
A*01:01-like Alleles	112	0.067	7338	0.073	0.91 (0.739-1.11)	0.363	0.581	5	0.063	5794	0.093	n/a		
**Other Alleles**														
Other A Alleles	505	0.301	30593	0.306	1.028 (0.897-1.179)	0.704	0.704	14	0.180	19006	0.306	0.487 (0.245-0.922)	**0.039**	0.078
Other B Alleles	767	0.457	53578	0.536	0.686 (0.591-0.798)	**0.000**	**0.000**	43	0.540	31650	0.510	1.19 (0.587-2.683)	0.659	0.979

Risk allele groups: Groups of HLA-A and HLA-B alleles with the same peptide binding pocket residues as 19 AA risk alleles identified in this study. Non-Risk allele groups: groups of alleles with same peptide binding pocket structures as the identified HLA-A and HLA-B Non-Risk alleles. In cases where insufficient number of patients had alleles from a particular allele group for statistically robust comparison, OR and p values could not be calculated (marked as not available, "n/a"). Cntrl, control. OR, Odds Ratio. CI, confidence interval; P, P-value; Padj, P value adjusted for multiple comparisons. Statistically significant P-value (<0.05) are **bolded**. For statistically significant comparisons, OR >1 are colored in red, and <1 are colored in blue. OR where the comparison showed significant differences with an OR discrepant to risk allele status are in purple.

Supplemental Table 7F. Association Analysis of AA with HLA allele groups in White NMDP Population

	White						
	Cases	Case Allele Freq	Cntrl	Control Allele Freq	OR (95% CI)	P	Padj
**Risk Allele -Like Group**							
Risk Pocket 1 Group (B*14:01 & B*14:02-like)	548	0.069	3845	0.039	1.832 (1.658-2.02)	**0.000**	**0.000**
Risk Pocket 2 Group (B*40:02 & B*41:02-like)	263	0.033	1887	0.019	1.791 (1.563-2.045)	**0.000**	**0.000**
Risk Pocket 3 Group (B*56:01, B*54:01, B*55:02-like)	188	0.024	2366	0.024	1.009 (0.863-1.172)	0.913	0.937
Risk Pocket 4 Group (A*02:01 & A*02:06-like)	2365	0.299	26219	0.263	1.205 (1.129-1.285)	**0.000**	**0.000**
A*33:03-like Alleles	180	0.023	1572	0.016	1.462 (1.243-1.709)	**0.000**	**0.000**
A*68:01-like Alleles	261	0.033	3198	0.032	1.047 (0.916-1.191)	0.498	0.498
A*74:01-like Alleles	9	0.001	190	0.002	0.602 (0.286-1.108)	0.138	0.220
B*07:02-like Alleles	1127	0.143	11982	0.120	1.225 (1.138-1.319)	**0.000**	**0.000**
B*08:01 like Alleles	949	0.120	10293	0.103	1.14 (1.053-1.233)	**0.001**	**0.003**
B*13:02-like Alleles	212	0.027	2292	0.023	1.161 (1-1.34)	0.045	0.066
B*18:01-like Alleles	314	0.040	4486	0.045	0.869 (0.768-0.979)	**0.023**	**0.037**
B*27:05-like Alleles	292	0.037	3424	0.034	1.08 (0.951-1.222)	0.238	0.310
B*38:02-like Alleles	0	0.000	110	0.001	n/a		
B*49:01-like Alleles	149	0.019	1883	0.019	1.007 (0.846-1.19)	0.937	0.937
B*50:02-like Alleles	136	0.017	1864	0.019	0.909 (0.756-1.083)	0.296	0.350
B*53:01-like Alleles	38	0.005	721	0.007	0.639 (0.448-0.881)	**0.009**	**0.017**
**Non Risk Allele -Like Group**							
NR Pocket 1 Group (A*03:01 & A*11:01-like)	1515	0.192	19694	0.197	0.963 (0.899-1.031)	0.277	0.317
NR Pocket 2 Group (A*23:01 & A*24:02-like)	762	0.097	11519	0.115	0.795 (0.73-0.864)	**0.000**	**0.000**
NR Pocket 3 (B*44:02 & B*44:03-like)	950	0.120	13397	0.134	0.874 (0.808-0.943)	**0.001**	**0.002**
A*01:01-like Alleles	1194	0.151	15416	0.154	0.952 (0.885-1.023)	0.185	0.246
**Other Alleles**							
Other A Alleles	1611	0.204	22008	0.220	0.895 (0.837-0.957)	**0.001**	**0.003**
Other B Alleles	2730	0.346	41264	0.413	0.688 (0.644-0.734)	**0.000**	**0.000**

Risk allele groups: Groups of HLA-A and HLA-B alleles with the same peptide binding pocket residues as 19 AA risk alleles identified in this study. Non-Risk allele groups: groups of alleles with same peptide binding pocket structures as the identified HLA-A and HLA-B Non-Risk alleles. In cases where insufficient number of patients had alleles from a particular allele group for statistically robust comparison, OR and p values could not be calculated (marked as not available, "n/a"). Cntrl, control. OR, Odds Ratio. CI, confidence interval; P, P-value; Padj, P value adjusted for multiple comparisons. Statistically significant P-value (<0.05) are **bolded**. For statistically significant comparisons, OR >1 are colored in red, and <1 are colored in blue. OR where the comparison showed significant differences with an OR discrepant to risk allele status are in purple.

Supplemental Table S8. Clinical characteristics of AA patients with and without Higher Pathogenicity Alleles (HPA).

Clinical characteristics			HPA Present (n=62)	No HPA (n=94)	OR	p
Demographics and Characteristics at Diagnosis	Age at diagnosis	Pediatric, n (%)	47 (79.7%)	74 (87.1%)	0.5845	0.2546
		Adult, n (%)	12 (20.3%)	11 (12.9%)		
		Not evaluable, n	3	9		
	AA severity at diagnosis	NSAA, n (%)	8 (15.1%)	12 (14.6%)	0.9645	1
		SAA, n (%)	21 (39.6%)	42 (51.2%)		
		VSAA, n (%)	24 (45.3%)	28 (34.1%)		
		Not evaluable, n	9	12		
	Cytogenetic abnormalities at diagnosis	Yes, n (%)	4 (7.0%)	2 (2.4%)	2.9946	0.2274
		No, n (%)	53 (93.0%)	80 (97.6%)		
		Not evaluable, n	5	12		
	Cellularity at diagnosis	Less than or equal to 5%, n (%)	22 (44.0%)	25 (31.6%)	1.6901	0.1897
		Greater than 5%, n (%)	28 (56.0%)	54 (68.4%)		
		Not evaluable, n	12	15		
Frontline Treatment	Frontline therapy Received	Frontline IST (hATG + CSA), n (%)	44 (74.6%)	69 (81.2%)		
		Frontline transplant, n	10 (17.0%)	10 (11.8%)		
		Other therapy (e.g., cyclosporine monotherapy), n	1 (1.7%)	1 (1.2%)		
		No therapy, n	4 (6.8%)	5 (5.9%)		
		Not evaluable, n	3	9		
	Time to frontline IST	<30 days, n (%)	21 (48.8%)	29 (42.6%)		0.6977
		30-90 days, n (%)	18 (41.9%)	34 (50.0%)		
		>90 days, n (%)	4 (9.3%)	5 (7.4%)		
		N/A (not IST or not evaluable), n	19	26		
Duration of follow-up for patients treated with frontline IST		<2 years, n (%)	4 (8.9%)	8 (11.3%)		0.9048
		2-5 years, n (%)	13 (28.9%)	21 (29.6%)		
		>5 years, n (%)	28 (62.2%)	42 (59.2%)		
		Not evaluable (frontline transplant or no data), n	17	23		

HPA (higher pathogenicity alleles): HLA-A*33:03, B*13:02, B*14:01, B*14:02, B*27:05, B*40:02, B*41:02, B*49:01 or B*56:01. IST, immunosuppressive therapy. SAA, severe aplastic anemia, VSAA, very severe aplastic anemia, NSAA, non-severe aplastic anemia; hATG, horse anti thymocyte globulin; CSA, cyclosporine A.

Supplemental Table S9: Clinical characteristics of pediatric and adult AA patients with and without Higher Pathogenicity Alleles (HPA)

Clinical Characteristic		Pediatric-onset AA					Adult-onset AA				
		**Total pediatric (n= 121)**	**HPA Present (n=47)**	**No HPA (n=74)**	**OR**	**p**	**Total adult (n=23)**	**HPA Present (n=12)**	**No HPA (n=11)**	**OR**	**p**
Age, median (range), years		9.1 (1.5-20.2)	8 (1.5-19.6)	9.6 (1.7-20.2)		0.327	46.9 (27.1-78.7)	42.1 (27.1-78.7)	48.8 (27.7-75)		0.779
**AA severity at diagnosis**	NSAA, n (%)	14 (12.4%)	7 (16.7%)	7 (9.9%)	0.5469	0.377	6 (27.3%)	1 (9.1%)	5 (45.5%)	8.33*	0.149
	SAA, n (%)	54 (47.8%)	15 (35.7%)	39 (54.9%)			9 (40.9%)	6 (54.5%)	3 (27.3%)		
	VSAA, n (%)	45 (39.8%)	20 (47.6%)	25 (35.2%)			7 (31.8%)	4 (36.4%)	3 (27.3%)		
	Not evaluable, n	8	5	3			1	1	0		
**Cellularity at diagnosis**	Less than or equal to 5%, n (%)	42 (38.2%)	18 (43.9%)	24 (34.8%)	1.467	0.418	5 (26.3%)	4 (44.4%)	1 (10.0%)	7.20	0.141
	Greater than 5%, n (%)	68 (61.2%)	23 (56.1%)	45 (65.2%)			14 (73.7%)	5 (55.6%)	9 (90.0%)		
	Not evaluable, n	11	6	5			4	3	1		
**Frontline therapy Re-ceived**	Frontline IST (hATG + CSA), n (%)	93 (76.9%)	33 (70.2%)	60 (81.1%)			20 (87.0%)	11 (91.7%)	9 (81.8%)		
	Frontline transplant, n	20 (16.5%)	10 (21.3%)	10 (13.5%)			0 (0.0%)	0 (0.0%)	0 (0.0%)		
	Other therapy (e.g., CSA mono-therapy), n	2 (1.7%)	1 (2.1%)	1 (1.4%)			0 (0.0%)	0 (0.0%)	0 (0.0%)		
	No therapy, n	6 (5.0%)	3 (6.4%)	3 (4.1%)			3 (13.0%)	1 (8.3%)	2 (18.2%)		
	Not evaluable, n	0	0	0			0	0	0		
**Time to front-line IST**	<30 days, n (%)	42 (45.7%)	14 (42.4%)	28 (47.5%)		0.551	8 (42.1%)	7 (70.0%)	1 (11.1%)		**0.031**
	30-90 days, n (%)	45 (48.9%)	16 (48.5%)	29 (49.2%)			7 (36.8%)	2 (20.0%)	5 (55.6%)		
	>90 days, n (%)	5 (5.4%)	3 (9.1%)	2 (3.4%)			4 (21.1%)	1 (10.0%)	3 (33.3%)		
	N/A (not IST or not evaluable), n	29	14	15			4	2	2		
**Duration of follow-up for patients treated**	<2 years, n (%)	8 (8.6%)	2 (6.1%)	6 (10.0%)		0.894	4 (17.4%)	2 (16.7%)	2 (18.2%)		0.845
	2-5 years, n (%)	29 (31.2%)	11 (33.3%)	18 (30.0%)			5 (21.7%)	2 (16.7%)	3 (27.3%)		
	>5 years, n (%)	56 (60.2%)	20 (60.6%)	36 (60.0%)			14 (60.9%)	9 (66.7%)	6 (54.5%)		
	Not evaluable (frontline trans-plant or no data), n	28	14	14			0	0	0		
	Duration of follow-up (median (range)), years	5.6 (0.1-113.9)	5.9 (1.4-16.3)	5.5 (0.1-113.9)			5.8 (0.1-112.7)	6.7 (0.5-112.7)	5.1 (0.1-107.4)		

HPA (higher pathogenicity alleles): HLA-A*33:03, B*13:02, B*14:01, B*14:02, B*27:05, B*40:02, B*41:02, B*49:01 or B*56:01. IST, immunosuppressive therapy. SAA, severe aplastic anemia, VSAA, very severe aplastic anemia, NSAA, non-severe aplastic anemia; hATG, horse anti thymocyte globulin; CSA, cyclosporine A.

Supplemental Table S10. Characteristics of AA patients in CIBMTR-Outcomes Cohort

Characteristic	Total Cohort	High risk alleles	Other HLA alleles
**No. of patients**	484	166	318
**No. of centers**	135	93	107
**Patient age, years - median (min-max)**	21 (0-73)	22 (2-73)	21 (0-71)
**Patient age at HSCT, years - no. (%)**			
<=10	98 (20)	31 (19)	67 (21)
11-17	87 (18)	32 (19)	55 (17)
18-29	126 (26)	46 (28)	80 (25)
30-39	59 (12)	18 (11)	41 (13)
40-49	37 (8)	14 (8)	23 (7)
50-59	47 (10)	18 (11)	29 (9)
60-69	28 (6)	6 (4)	22 (7)
70+	2 (0)	1 (1)	1 (0)
**Sex - no. (%)**			
Male	268 (55)	100 (60)	168 (53)
Female	216 (45)	66 (40)	150 (47)
**Race - no. (%)**			
Caucasian	350 (72)	101 (61)	249 (78)
Hispanic	41 (8)	23 (14)	18 (6)
African-American	31 (6)	16 (10)	15 (5)
Native American	4 (1)	1 (1)	3 (1)
Asian/Pacific Islander	27 (6)	12 (7)	15 (5)
Other	8 (2)	3 (2)	5 (2)
Missing	23 (5)	10 (6)	13 (4)
**Donor group - no. (%)**			
HLA-identical sibling	79 (16)	30 (18)	49 (15)
8/8 plus well matched unrelated	405 (84)	136 (82)	269 (85)
**HCT-CI - no. (%)**			
0	178 (37)	59 (36)	119 (37)
1-2	93 (19)	32 (19)	61 (19)
3+	98 (20)	40 (24)	58 (18)
NA (data not collected prior to 2007)	115 (24)	35 (21)	80 (25)
**Karnofsky score - no. (%)**			

<90	115 (24)	38 (23)	77 (24)
>=90	351 (73)	122 (73)	229 (72)
Missing	18 (4)	6 (4)	12 (4)
**Conditioning regimen intensity - no. (%)**			
MAC	7 (1)	1 (1)	6 (2)
RIC	1 (0)	0 (0)	1 (0)
NMA	476 (98)	165 (99)	311 (98)
**Conditioning regimen - no. (%)**			
TBI/Cy	98 (20)	27 (16)	71 (22)
TBI/Cy/Flu	178 (37)	68 (41)	110 (35)
Cy/Flu	100 (21)	35 (21)	65 (20)
Cy alone	108 (22)	36 (22)	72 (23)
**PNH - no. (%)**			
Negative	233 (48)	79 (48)	154 (48)
Positive	69 (14)	34 (20)	35 (11)
Missing	182 (38)	53 (32)	129 (41)
**Blood counts at diagnosis—median (range)**			
WBC ($x10^9$/L)	3 (0-10)	2 (0-10)	3 (0-9)
Hemoglobin (g/dL)	8 (2-17)	8 (3-14)	8 (2-17)
Platelet count ($x10^9$/L)	13 (0-197)	14 (0-170)	13 (0-197)
Absolute neutrophil count ($x10^9$/L)	1 (0-6)	1 (0-6)	1 (0-6)
Absolute reticulocyte count ($x10^9$/L)	11 (0-210)	10 (0-67)	16 (0-210)
**Graft type - no. (%)**			
Peripheral blood	54 (11)	22 (13)	32 (10)
Bone marrow	430 (89)	144 (87)	286 (90)
**Immunosuppressive therapy prior to HSCT - no. (%)**			
Yes	382 (79)	132 (80)	250 (79)
Missing	102 (21)	34 (20)	68 (21)
**ATG immunosuppression - no. (%)**			
No	35 (7)	14 (8)	21 (7)
Yes	358 (74)	123 (74)	235 (74)
Missing	91 (19)	29 (17)	62 (19)
**CSA immunosuppression - no. (%)**			
No	42 (9)	15 (9)	27 (8)
Yes	254 (52)	94 (57)	160 (50)

Missing	188 (39)	57 (34)	131 (41)
**GVHD prophylaxis - no. (%)**			
Ex-vivo T-cell depletion	3 (1)	0 (0)	3 (1)
Post-CY + other(s)	5 (1)	0 (0)	5 (2)
CSA/TAC + MMF ± other(s) (except post-CY)	65 (13)	24 (14)	41 (13)
CSA/TAC + MTX ± other(s) (except post-CY)	340 (70)	114 (69)	226 (71)
CSA/TAC + other(s) (except MMF, MTX, post-CY)	25 (5)	12 (7)	13 (4)
CSA/TAC alone	35 (7)	11 (7)	24 (8)
Other	1 (0)	1 (1)	0 (0)
Missing	10 (2)	4 (2)	6 (2)
**Year of transplant - no. (%)**			
1988-1999	10 (2)	2 (1)	8 (3)
2000-2009	183 (38)	49 (30)	134 (42)
2010-2018	291 (60)	115 (69)	176 (55)
**Unrelated donor age, years - median (range)**	31 (19-53)	30 (19-49)	32 (20-53)
**Follow-up, months, median (range)**	58 (3-241)	55 (3-198)	59 (3-241)

no., number. HSCT, hematopoietic stem cell transplantation, ATG, anti-thymocyte globulin, CSA, cyclosporine A, GVHD, graft-versus-host disease; CY, cyclophosphamide; TAC, tacrolimus; MMF, mycophenolate mofetil; MTX, methotrexate.

Supplemental Table S11: Multivariate analysis of alloHSCT outcomes of AA patients with and without Higher Pathogenicity Alleles (HPA).

	**Graft failure**			**OS**			**Grade 2-4 aGVHD**			**cGVHD**			**Neutrophil En-graftment**			**Platelet en-graftment**		
	**N**	**HR (95 % CI)**	**P**	**N**	**HR (95% CI)**	**P**	**N**	**HR (95 % CI)**	**P**	**N**	**HR (95 % CI)**	**P**	**N**	**HR (95 % CI)**	**P**	**N**	**HR (95 % CI)**	**P**
**AA HLA Risk Allele Group**																		
No HPA Alleles	309	1.00	0.115	318	1.00	0.569	317	1.00	0.350	310	1.00	0.338	316	1.00	0.454	312	1.00	0.148
**HPA alleles**	164	0.57 (0.22-1.43)	0.115	166	0.88 (0.56-1.38)	0.569	163	0.82 (0.55-1.24)	0.350	165	1.18 (0.84-1.66)	0.338	166	0.93 (0.76-1.13)	0.454	166	1.16 (0.89-1.50)	0.148
**Age group, years**																		
<=17	179	1.00	**0.031**	185	1.00	**0.001**				181	1.00	**0.042**						
18-39	183	1.73 (0.62-4.84)	**0.168**	185	1.77 (0.93-3.37)	**0.081**				181	1.71 (1.16-2.51)	**0.006**						
40-59	81	3.15 (1.05-9.51)	**0.007**	84	2.84 (1.38-5.84)	**0.005**				83	1.69 (1.03-2.78)	**0.040**						
>=60	30	3.35 (0.81-13.78)	**0.028**	30	5.11 (2.09-12.50)	**<.001**				30	1.91 (0.91-4.00)	0.088						
**Donor group**																		
MSD				79	1.00	**0.004**	78	1.00	0.010							79	1.00	**0.025**
≥8/8 MUD				405	7.63 (1.92-30.30)	**0.004**	402	2.60 (1.26-5.34)	0.010							399	0.75 (0.55-1.04)	**0.025**

**Karnofsky score**																		
≥90				351	1.00	**0.008**												
<90				115	1.27 (0.69-2.31)	**0.443**												
Missing				18	3.74 (1.62-8.65)	**0.002**												
**Conditioning regimens**																		
Flu/CY/TBI + (ATG or Camp)							175	1.00	**<.001**									
Cy/TBI/ATG + 200 cGy TBI							98	1.81 (1.33-2.46)	**<.001**									
Flu/Cy + (ATG or Camp)							100	0.59 (0.34-1.04)	0.068									
CY + (ATG or Camp)							107	0.88 (0.49-1.57)	0.663									

HPA (higher pathogenicity alleles): HLA-A*33:03, B*13:02, B*14:01, B*14:02, B*27:05, B*40:02, B*41:02, B*49:01 or B*56:01. aGVHD, acute graft-versus-host disease; cGVHD, chronic GVHD; HR, hazard ratio. ATG, anti-thymocyte globulin; CY, cyclophosphamide; TBI, total body irradiation; Flu, fludarabine; Camp, Campath.

Supplemental Table S12. AA patient characteristics for exploratory analysis of the effect of Higher Pathogenicity Allele concordance in donor-recipient pairs on outcomes of haploidentical alloSCT for AA.

Clinical Characteristic	Total Cohort	HPA Concordant donor-recipient HSCT	HPA Discordant donor-recipient HSCT
**No. of patients**	29	15	14
**No. of centers**	20	12	12
**Patient age, years - median (range)**	21 (2-65)	18 (2-65)	22 (7-38)
**Patient age at HSCT, years - no. (%)**			
<=10	7 (24)	5 (33)	2 (14)
11-17	6 (21)	3 (20)	3 (21)
18-29	9 (31)	3 (20)	6 (43)
30-39	4 (14)	1 (7)	3 (21)
40-49	1 (3)	1 (7)	0 (0)
50-59	0 (0)	0 (0)	0 (0)
60-69	2 (7)	2 (13)	0 (0)
**Sex - no. (%)**			
Male	23 (79)	12 (80)	11 (79)
Female	6 (21)	3 (20)	3 (21)
**Race - no. (%)**			
Caucasian	7 (24)	4 (27)	3 (21)
Hispanic	5 (17)	3 (20)	2 (14)
African-American	8 (28)	4 (27)	4 (29)
Native American	1 (3)	0 (0)	1 (7)
Asian/Pacific Islander	0 (0)	0 (0)	0 (0)
Other	2 (7)	1 (7)	1 (7)
Missing	6 (21)	3 (20)	3 (21)
**HCT-CI - no. (%)**			
0	11 (38)	6 (40)	5 (36)
1-2	12 (41)	6 (40)	6 (43)

3+	6 (21)	3 (20)	3 (21)
**Karnofsky score - no. (%)**			
<90	12 (41)	5 (33)	7 (50)
≥90	17 (59)	10 (67)	7 (50)
**Conditioning regimen intensity - no. (%)**			
MAC	0 (0)	0 (0)	0 (0)
RIC	9 (31)	5 (33)	4 (29)
NMA	20 (69)	10 (67)	10 (71)
**Conditioning regimen - no. (%)**			
TBI/Cy/Flu	27 (93)	14 (93)	13 (93)
Cy/Flu	1 (3)	1 (7)	0 (0)
Others	1 (3)	0 (0)	1 (7)
**PNH - no. (%)**			
Negative	11 (38)	6 (40)	5 (36)
Positive	7 (24)	3 (20)	4 (29)
Missing	11 (38)	6 (40)	5 (36)
**Blood counts at diagnosis—median (range)**			
WBC ($x10^9$/L)	2 (0-5)	2 (1-5)	2 (0-4)
Hemoglobin (g/dL)	7 (3-13)	6 (4-11)	9 (3-13)
Platelet count ($x10^9$/L)	25 (1-50)	25 (1-50)	25 (1-45)
Absolute neutrophil count ($x10^9$/L)	1 (0-5)	0 (0-5)	1 (0-1)
Absolute reticulocyte count ($x10^9$/L)	8 (0-22)	11 (0-22)	6 (0-16)
**Graft type - no. (%)**			
Peripheral blood	11 (38)	5 (33)	6 (43)
Bone marrow	18 (62)	10 (67)	8 (57)
**Immunosuppressive therapy prior to HSCT - no. (%)**			
Yes	25 (86)	13 (87)	12 (86)
Missing	4 (14)	2 (13)	2 (14)

**ATG immunosuppression - no. (%)**			
No	2 (7)	2 (13)	0 (0)
Yes	24 (83)	12 (80)	12 (86)
Missing	3 (10)	1 (7)	2 (14)
**CSA immunosuppression - no. (%)**			
No	5 (17)	3 (20)	2 (14)
Yes	21 (72)	11 (73)	10 (71)
Missing	3 (10)	1 (7)	2 (14)
**GVHD prophylaxis - no. (%)**			
Ex-vivo T-cell depletion	1 (3)	1 (7)	0 (0)
Post-CY + other(s)	21 (72)	11 (73)	10 (71)
CSA/TAC + MMF ± other(s) (except post-CY)	6 (21)	3 (20)	3 (21)
CSA/TAC + MTX ± other(s) (except post-CY)	0 (0)	0 (0)	0 (0)
Missing	1 (3)	0 (0)	1 (7)
**Year of transplant - no. (%)**			
2010-2018	29 (100)	15 (100)	14 (100)
**Follow-up, months - median (range)**	30 (6-67)	33 (6-67)	29 (17-52)

HPA (higher pathogenicity alleles): HLA-A*33:03, B*13:02, B*14:01, B*14:02, B*27:05, B*40:02, B*41:02, B*49:01 or B*56:01.

Supplemental Table S13: Univariate analysis of the effects of High Pathogenicity Alleles (HPA) concordance in donor-recipient pairs on outcomes after haploidentical alloSCT for AA.

Outcomes	HPA-concordant* HSCT (N = 15)	HPA-discordant HSCT (N = 14)	P Value
	Probability, % (95% CI)	Probability, % (95% CI)	
**Neutrophil engraftment**			0.258
1 months	86.7 (61.9-99.3)	71.4 (43.8-92.4)	
**Platelet recovery**			0.095
1 months	46.7 (21.9-72.4)	21.4 (4.3-46.9)	
6 months	100	71.7 (26.9-99.1)	
**Graft failure**			0.331
1-year	21.3 (4.1-47.1)	35.7 (12.8-62.8)	
2-year	21.3 (4.1-47.1)	35.7 (12.8-62.8)	

* Patients were stratified by risk allele concordant or risk allele discordant status for the 9 Higher Pathogenicity Alleles (HPA) (HLA-A*33:03, B*13:02, B*14:01, B*14:02, B*27:05, B*40:02, B*41:02, B*49:01 or B*56:01) in donor-recipient pairs.

Supplemental Table S14: AA patient characteristics for exploratory analysis of the effect of HLA risk allele concordance in donor-recipient pairs on outcomes after haploidentical alloSCT for AA.

Clinical Characteristic	Total Cohort	Matched for all Risk Alleles	Mismatched for All Risk Alleles
**No. of patients**	41	25	16
**No. of centers**	26	17	14
**Patient age, years - median (range)**	21 (2-74)	23 (2-74)	18 (2-46)
**Patient age at HSCT, years - no. (%)**			
<=10	8 (20)	4 (16)	4 (25)
11-17	11 (27)	6 (24)	5 (31)
18-29	13 (32)	10 (40)	3 (19)
30-39	3 (7)	0 (0)	3 (19)
40-49	2 (5)	1 (4)	1 (6)
50-59	1 (2)	1 (4)	0 (0)
60-69	1 (2)	1 (4)	0 (0)
70+	2 (5)	2 (8)	0 (0)
**Sex - no. (%)**			
Male	29 (71)	18 (72)	11 (69)
Female	12 (29)	7 (28)	5 (31)
**Race - no. (%)**			
Caucasian	10 (24)	7 (28)	3 (19)
Hispanic	5 (12)	1 (4)	4 (25)
African-American	15 (37)	10 (40)	5 (31)
Native American	0 (0)	0 (0)	0 (0)
Asian/Pacific Islander	2 (5)	1 (4)	1 (6)
Other	2 (5)	1 (4)	1 (6)
Missing	7 (17)	5 (20)	2 (13)
**HCT-CI - no. (%)**			
0	15 (37)	7 (28)	8 (50)
1-2	11 (27)	6 (24)	5 (31)

3+	15 (37)	12 (48)	3 (19)
**Karnofsky score - no. (%)**			
<90	14 (34)	9 (36)	5 (31)
≥90	27 (66)	16 (64)	11 (69)
**Conditioning regimen intensity - no. (%)**			
MAC	3 (7)	3 (12)	0 (0)
RIC	9 (22)	6 (24)	3 (19)
NMA	28 (68)	15 (60)	13 (81)
TBD	1 (2)	1 (4)	0 (0)
**Conditioning regimen - no. (%)**			
TBI/Cy/Flu	34 (83)	19 (76)	15 (94)
Cy/Flu	1 (2)	1 (4)	0 (0)
Others	6 (15)	5 (20)	1 (6)
**PNH - no. (%)**			
Negative	20 (49)	11 (44)	9 (56)
Positive	10 (24)	7 (28)	3 (19)
Missing	11 (27)	7 (28)	4 (25)
**Blood counts at diagnosis—median (range)**			
WBC ($x10^9$/L)	2 (0-7)	2 (0-7)	2 (0-5)
Hemoglobin (g/dL)	8 (3-13)	8 (3-13)	8 (3-13)
Platelet count ($x10^9$/L)	17 (1-542)	16 (1-542)	17 (1-52)
Absolute neutrophil count ($x10^9$/L)	1 (0-5)	0 (0-5)	1 (0-1)
Absolute reticulocyte count ($x10^9$/L)	15 (0-217)	14 (0-217)	15 (1-84)
**Graft type - no. (%)**			
Peripheral blood	14 (34)	10 (40)	4 (25)
Bone marrow	27 (66)	15 (60)	12 (75)
**Immunosuppressive therapy prior to HSCT - no. (%)**			
Yes	35 (85)	23 (92)	12 (75)
Missing	6 (15)	2 (8)	4 (25)

**ATG immunosuppression - no. (%)**			
No	6 (15)	6 (24)	0 (0)
Yes	30 (73)	18 (72)	12 (75)
Missing	5 (12)	1 (4)	4 (25)
**CSA immunosuppression - no. (%)**			
No	5 (12)	3 (12)	2 (13)
Yes	31 (76)	21 (84)	10 (63)
Missing	5 (12)	1 (4)	4 (25)
**GVHD prophylaxis - no. (%)**			
Ex-vivo T-cell depletion	3 (7)	3 (12)	0 (0)
Post-CY + other(s)	31 (76)	20 (80)	11 (69)
CSA/TAC + MMF ± other(s) (except post-CY)	6 (15)	2 (8)	4 (25)
CSA/TAC + MTX ± other(s) (except post-CY)	0 (0)	0 (0)	0 (0)
Missing	1 (2)	0 (0)	1 (6)
**Year of transplant - no. (%)**			
2010-2018	41 (100)	25 (100)	16 (100)
**Follow-up, months - median (range)**	31 (6-60)	30 (6-60)	35 (17-53)

Supplemental Table S15: Univariate analysis of the effects of HLA risk allele concordance in donor-recipient pairs on outcomes after haploidentical alloSCT for AA.

Outcomes	Risk Allele* Concordant HSCT (N = 25)	Risk Allele* Discordant HSCT (N = 16)	P Value
	Probability, % (95% CI)	Probability, % (95% CI)	
**Neutrophil engraftment**			0.257
1 months	80 (61.5-93.5)	75 (49.8-93.4)	
**Platelet recovery**			0.51
1 months	52 (32.2-71.5)	31.3 (11-56.2)	
6 months	80 (51.1-97.7)	77.1 (45-97.4)	
**Graft failure**			0.676
1-year	32.5 (15.4-52.4)	25 (7.1-49.2)	
2-year	32.5 (15.4-52.4)	25 (7.1-49.2)	

* Patients were stratified by risk allele concordant or risk allele discordant status for any of the 19 risk alleles in donor-recipient pairs.

Supplemental References

1. Babushok DV, Duke JL, Xie HM, et al. Somatic HLA Mutations Expose the Role of Class I-Mediated Autoimmunity in Aplastic Anemia and its Clonal Complications. *Blood Adv*. 2017;1(22):1900-1910.
2. Shimano KA, Narla A, Rose MJ, et al. Diagnostic work-up for severe aplastic anemia in children: Consensus of the North American Pediatric Aplastic Anemia Consortium. *Am J Hematol*. 2021;96(11):1491-1504.
3. Peslak SA, Olson T, Babushok DV. Diagnosis and Treatment of Aplastic Anemia. *Curr Treat Options Oncol*. 2017;18(12):70.
4. Camitta BM. Pathogenesis and treatment of aplastic anemia. *Rinsho Ketsueki*. 1984;25(4):459-469.
5. American Academy of Pediatrics Council on Child and Adolescent Health: Age limits of pediatrics. *Pediatrics*. 1988;81(5):736.
6. Babushok DV, Xie HM, Roth JJ, et al. Single nucleotide polymorphism array analysis of bone marrow failure patients reveals characteristic patterns of genetic changes. *Br J Haematol*. 2014;164(1):73-82.
7. Duke JL, Lind C, Mackiewicz K, et al. Determining performance characteristics of an NGS-based HLA typing method for clinical applications. *HLA*. 2016;87(3):141-152.
8. McKenna A, Hanna M, Banks E, et al. The Genome Analysis Toolkit: a MapReduce framework for analyzing next-generation DNA sequencing data. *Genome Res*. 2010;20(9):1297-1303.
9. Shah YB, Priore SF, Li Y, et al. The predictive value of PNH clones, 6p CN-LOH, and clonal TCR gene rearrangement for aplastic anemia diagnosis. *Blood Adv*. 2021;5(16):3216-3226.
10. Sarkizova S, Klaeger S, Le PM, et al. A large peptidome dataset improves HLA class I epitope prediction across most of the human population. *Nat Biotechnol*. 2020;38(2):199-209.
11. Carreno BM, Becker-Hapak M, Chan M, et al. Amino-terminal extended peptide single-chain trimers are potent synthetic agonists for memory human CD8+ T cells. *J Immunol*. 2012;188(12):5839-5849.
12. Garson D, Dokhelar MC, Wakasugi H, Mishal Z, Tursz T. HLA class-I and class-II antigen expression by human leukemic K562 cells and by Burkitt-K562 hybrids: modulation by differentiation inducers and interferon. *Exp Hematol*. 1985;13(9):885-890.
13. Silver ML, Guo HC, Strominger JL, Wiley DC. Atomic structure of a human MHC molecule presenting an influenza virus peptide. *Nature*. 1992;360(6402):367-369.
14. Gorga JC, Madden DR, Prendergast JK, Wiley DC, Strominger JL. Crystallization and preliminary X-ray diffraction studies of the human major histocompatibility antigen HLA-B27. *Proteins*. 1992;12(1):87-90.
15. Madden DR. The three-dimensional structure of peptide-MHC complexes. *Annu Rev Immunol*. 1995;13:587-622.
16. Guo HC, Jardetzky TS, Garrett TP, Lane WS, Strominger JL, Wiley DC. Different length peptides bind to HLA-Aw68 similarly at their ends but bulge out in the middle. *Nature*. 1992;360(6402):364-366.
17. Sidney J, Peters B, Frahm N, Brander C, Sette A. HLA class I supertypes: a revised and updated classification. *BMC Immunol*. 2008;9:1.
18. Wagih O. ggseqlogo: a versatile R package for drawing sequence logos. *Bioinformatics*. 2017;33(22):3645-3647.
19. Madbouly A, Gragert L, Freeman J, et al. Validation of statistical imputation of allele-level multilocus phased genotypes from ambiguous HLA assignments. *Tissue Antigens*. 2014;84(3):285-292.
20. Gragert L, Fingerson S, Albrecht M, Maiers M, Kalaycio M, Hill BT. Fine-mapping of HLA associations with chronic lymphocytic leukemia in US populations. *Blood*. 2014;124(17):2657-2665.
21. Gragert L, Madbouly A, Freeman J, Maiers M. Six-locus high resolution HLA haplotype frequencies derived from mixed-resolution DNA typing for the entire US donor registry. *Hum Immunol*. 2013;74(10):1313-1320.
22. Benjamini Y, Hochberg Y. Controlling The False Discovery Rate - A Practical And Powerful Approach To Multiple Testing. *J Royal Statist Soc, Series B*. 1995;57:289-300.
23. Mayor NP, Wang T, Lee SJ, et al. Impact of Previously Unrecognized HLA Mismatches Using Ultrahigh Resolution Typing in Unrelated Donor Hematopoietic Cell Transplantation. *J Clin Oncol*. 2021;39(21):2397-2409.
24. Spellman S, Setterholm M, Maiers M, et al. Advances in the selection of HLA-compatible donors: refinements in HLA typing and matching over the first 20 years of the National Marrow Donor Program Registry. *Biol Blood Marrow Transplant*. 2008;14(9 Suppl):37-44.
25. Zaimoku Y, Patel BA, Adams SD, et al. HLA associations, somatic loss of HLA expression, and clinical outcomes in immune aplastic anemia. *Blood*. 2021.
